# The Tensile Behaviour of Highly Filled High-Density Polyethylene Quaternary Composites: Weld-Line Effects, DIC Curiosities and Shifted Deformation Mechanisms

**DOI:** 10.3390/polym13040527

**Published:** 2021-02-10

**Authors:** David Viljoen, Matthieu Fischer, Ines Kühnert, Johan Labuschagné

**Affiliations:** 1 Department of Chemical Engineering, Institute of Applied Materials, University of Pretoria, Hatfield, Pretoria 0002, South Africa; johan.labuschagne@up.ac.za; 2 Polymer Processing Department, Leibniz-Institut für Polymerforschung Dresden e.V., 01069 Dresden, Germany; fischer-matthieu@ipfdd.de (M.F.); kuehnert@ipfdd.de (I.K.)

**Keywords:** experimental design, high-density polyethylene, composite, digital image correlation, tensile testing, mixture model, deformation mechanics, calcium carbonate, carbon black, stabiliser, weld line

## Abstract

The interactive effects between additives and weld lines, which are frequent injection-moulding defects, were studied in high-density polyethylene (HDPE) and compared to weld-line-free reference samples. These materials were formulated around a D- and I-optimal experimental design, based on a quadratic Scheffé polynomial model, with up to 60 wt% calcium carbonate, masterbatched carbon black and a stabiliser package. Where reasonable and appropriate, the behaviours of the systems were modelled using statistical techniques, for a better understanding of the underlying trends. The characterisations were performed through the use of conventional tensile testing, digital image correlation (DIC) and scanning electron microscopy (SEM). A range of complex interactive effects were found during conventional tensile testing, with DIC used to better understand and explain these effects. SEM is used to better understand the failure mechanics of some of these systems through fractography, particularly regarding particle effects. A measure is introduced to quantify the deviation of the pre-yield deformation curve from the ideal elastic case. Novel analysis of DIC results is proposed, through the use of combined time-series plots and measures quantifying the extent and localisation of peak deformation. Through this, it could be found that strong shifts in the deformation mechanisms occur as a function of formulation and the presence/absence of weld lines. Primarily, changes are noted in the onset of continuous inter- and intralamellar slip and cavitation/fibrillation, seen through the onset of localised deformation and stress-whitening.

## 1. Introduction

HDPE is among the rare commodity polymers that may be modified—through the selective addition of fillers—to be useful in engineering-type applications [1]. This has become all-the-more common in recent times as a result of innovations in the compatibilisation of mineral fillers—such as calcium carbonate—that have dramatically improved the mechanical properties that may be achieved in composites containing these materials [2]. Given the substantial cost benefits that these filled materials may offer [3] and the drive to reduce the dependency of plastics on materials derived from fossil fuels [1], this is a very promising development.

A key concern in these materials, however, is the effect that common manufacturing defects may have on their mechanical properties. For use in engineering-type applications, injection-moulding is a common manufacturing process. Perhaps the most important, most common and the best-studied defect that may be encountered in this process is the weld line [4,5,6,7,8,9]. Weld lines result from the meeting of multiple melt fronts: head-on (“stagnation”) or at an angle (“continuous flow”) [10]. These defects have been studied across a wide range of materials, ranging from virgin polymers [10] to those highly filled with fibrous reinforcements [11,12]. Weld lines, however, are rarely studied in the materials described in the prior paragraph, and more rarely still across a wide range of formulations.

V-notches—surface grooves circumscribing the weld-area, resulting from, for example, anisotropic shrinkage and air trapped by the convergence of melt fronts—frequently accompany weld lines [11,13,14]. In addition to their obvious effects as surface blemishes, these defects may have substantial deleterious effects on the mechanical performance of specimens: acting as stress concentrators, in addition to bisecting the highly oriented skin layer of a moulding [10,14].

Tensile testing is one of the most common techniques used in the characterisation of composite materials. In its conventional form, this technique allows for the straightforward determination of the effects of weld lines on the mechanical performance of specimens, usually viewed in comparison with the performance of weld-line-free (henceforth denoted REF, for “reference”) specimens. Frequently, this comparison is presented as a “weld-line factor” (WLF) [10,15], expressed as the mean performance of weld-line (henceforth denoted WL) specimens of a material divided by the mean performance of REF specimens of that same material. Typically, filled materials exhibit slightly to substantially smaller WLFs than their unfilled equivalents [15]. In the case of isotropic fillers, this results from the unfavourable orientation of the fillers parallel to the weld line, interrupting reinforcement [5,8,10]. A similar trend is seen in highly crystalline polymers, with these typically exhibiting smaller WLFs than that seen in amorphous polymers [10].

Over the years, many have worked to develop a mechanistic understanding of the way in which semi-crystalline polymers—here with the focus on HDPE—are deformed under uniaxial load. Great progress was made by the Strobl group [16,17,18,19,20,21] building on the works of Bowden and coworkers [22,23], Fotheringham and Cherry [24], and Lohse and Gaylord [25], among many others. Further progress has been made in recent times, notably by Farge and coworkers on the fibrillation/cavitation process and its effects [26]. The established mechanism may be summarised as (chronologically) [16,17,18,19,20,21,25,26]:

1. The elongation and orientation of the amorphous regions between the lamellae;
2. The onset of nucleated inter- and intralamellar slips;
3. The collective inter- and intralamellar slips;
4. The onset of fibrillation; and
5. The onset of chain disentanglement.

While conventional tensile testing has its place, certainly in terms of the ready comparison of a range of materials, it may be supplemented with techniques that offer more detailed information on the deformation of the specimens. While a variety of techniques exist that may allow such study [27], digital image correlation (DIC) is among the most facile. It is performed by depositing a fine spray of contrasting paint on a specimen, with said specimen subjected to mechanical characterisation under the surveillance of a suitable and suitably positioned camera. The footage of the deformation may be analysed algorithmically: essentially tracking the areas circumscribed by the speckles through space and time [28]. This allows the deformation of the sample to be tracked and digitised in multiple dimensions, with fairly high spacial resolution. Care must be taken, however, to balance spacial resolution and accuracy—through the careful calibration of the size and spacing/density of the speckles on the specimen [29]. The resultant data may be flattened—for example by determining the deformation along a line across the sample—for ready comparison. This is, perhaps, the most common application of the DIC technique in comparative work [11,12,30]. Godara et al. [31] built on this to show the deformation profile of a specimen at four distinct global strains—enabling the chronological development of the strain distribution to be better understood.

This technique has been applied to the study of a range of composite materials, with and without weld lines. This includes HDPE filled with calcium carbonate, albeit only at comparatively low loadings and in the absence of weld lines [32]. Notable in the prior study is the use of three-dimensional DIC, allowing the volumetric deformation of the system to be estimated. This suggested the presence of substantial particle debonding—resulting in the increase of volumetric strain with an increase in filler loading—although such suggestions must be taken with care, given the dangers of assuming homogeneous distributions of strain through a tensile sample [33]. It has been established that, at least in the case of uncompatibilised materials, a substantial portion of the late-stage deformation of a composite specimen may be ascribed to debonding. For example, Sudár et al. [34] found that around 80 percent of the particles in an HDPE/calcium carbonate composite could be subject to debonding. This may, of course, differ when additional materials are added to the matrix and filler, as shown by Yang et al. [35]. These authors found that a surface-treated calcium carbonate filler exhibited the same volume strain/debonding behaviour as that found by Sudár et al. [34], but that this behaviour was reduced dramatically through the addition of a compatibilised elastomer. These findings were confirmed through the use of X-ray densitometry.

Fractography is a versatile technique—applied over a range of scales, from those of visual inspection and optical microscopy to that of scanning electron microscopy—for the study of the cause and mechanism of material failure [36]. It is particularly useful in the study of composite materials, as the effects of the fillers may be better understood through the identification of agglomerates and areas affected by cavitation, debonding and fibrillation, among others. Deductions may be made on the type of failure—in the range between brittle and ductile—as a function of location, based on the morphology of the failure surfaces, and the origin/s of failure may be identified [36,37].

Given the above-mentioned benefits of mineral fillers, it is tempting to increase the loadings of these fillers as far as possible. In the simplest terms, this would be limited in terms of the packing density of the material. However, the viscosity of the material tends to infinity as the maximum packing density is approached [38]. Within the processing limits, there are also limits in terms of practical use. Notably, it is well-established that higher loadings of mineral fillers increase the stiffness of composites [38]. This, usually, is accompanied by the failure mode of the materials tending towards being that of brittle failure [39]. Incidentally, the prior study explores the highest loading of calcium carbonate yet seen in studies investigating the solid properties of HDPE by the authors, at a claimed 65 wt%. Through careful tailoring of the materials and loadings involved, the failure mechanism can be altered to progress from ductile to brittle to ductile and back to brittle with increasing filler loading, through the formation (in this second ductile region) of continuous highly oriented sections of matrix material around the filler particles (effectively overlapping shells around the particles). When deformed, these sections partially debond from the filler particles and form ligaments [40]. This allows for dramatic improvements in the toughness of the material, when the criteria governing this behaviour are met. This toughening mechanism is not dissimilar to that brought about by the inclusion a dispersed rubber phase in the matrix [40] and is likely to result in a similar temperature shift of the deformation mechanics as that seen through the addition of the dispersed rubber phase [41].

While the simple composites of HDPE and calcium carbonate are already compelling, more interesting are the composites of these materials with additional additives—and the interactions that result. A previous work by the authors showed the substantial improvements in thermal stability that may be achieved in these systems through interactive effects [42], addressing the traditional concern over the stability of highly filled systems in melt processing. The effects seen in that study may influence the mechanical performance of these materials, for example by reducing/eliminating the presence of cross-linked degradation products, in addition to other effects not related to thermal stability. The stabilisers used here are among the most common additives to make use of synergistic interactions, so much so that some (secondary antioxidants with phosphite chemistry, for example) offer no benefit on their own, but greatly enhance the performance of others (phenolic primary antioxidants, to continue the example) [43]. Carbon black, traditionally used as only a pigment, interacts with these stabilisers (with the interaction dependent on its surface groups) in the thermal and photo-stability of materials [43], and may mirror calcium carbonate in acting as a reinforcing particulate filler—more so when added in the presence of a compatibiliser [44].

From the above, it is clear that the behaviour of weld lines—particularly in the case of highly filled materials and those with numerous additives—is not yet fully studied or understood. The interactions to be found in these systems are complex, and the statistical techniques that may aid in their understanding are rarely used. Further, the distribution/localisation of strain as a function of time/global strain is rarely reported in anything more than large steps, obfuscating the analysis of the deformation of a specimen leading up to its failure. Finally, little to none of the literature discussing the changes in the deformation mechanism of composites over a range of formulations may be found, particularly coupled with the effects of weld lines.

In this work, it is sought to gather data on the tensile behaviour of an HDPE/calcium carbonate/masterbatched carbon black/stabiliser composite system in specimens with and without weld lines. These data are to be used for the conventional tensile analysis of the system (including WLFs) as a function of formulation, for the deeper analysis of the system in terms of the deformation mechanisms seen via DIC and SEM, and for the statistical modelling of various metrics in an effort to better understand the interactions between the matrix and the additives. The DIC work and related measures were considered necessary, as a result of complex behaviour observed as a function of formulation and the presence/absence of weld lines in a number of the conventional tensile testing measures. A modification in the way that axial DIC data are presented is suggested, in addition to a number of related metrics, as a means to better understanding the development of local strain as a function of time/global strain. Finally, novel measures are presented to quantify deviation from ideal elastic deformation prior to yield (in conventional tensile testing) and the strength and localisation of the local deformation peaks that may be observed through DIC analysis.

The effects of changes to the processing parameters of the materials were not studied in this work, to place some constraint on the number of variables. It was not possible to determine whether any changes in filler loading occurred on the weld line interface.

## 2. Materials and Methods

### 2.1. Materials

A range of commercial materials were used in this study. The matrix was selected as an injection-moulding grade of HDPE (Dow HDPE 25055E, Midland, MI, USA), while the primary filler was selected as an ultra-fine, surface-coated calcium carbonate with a median particle size of 0.8 μm [45] (OMYA Hydrocarb^®^ 95 T-OG, Oftringen, Switzerland). The further additives consisted of a stabiliser package of BASF (Rhineland-Palatinate, Germany) Chimassorb 944 and BASF Irgafos 168, held at a constant 3:2 ratio, and a carbon black masterbatch (Caparol NEFA MB F 21504 Schwarz, Ober-Ramstadt, Germany). The carbon black masterbatch consists of a 28% loading of a furnace black with an average particle size of 16 nm [46] (Orion Engineered Carbons Printex^®^ F 80, Luxembourg) in a styrene-ethylene/butylene-styrene (SEBS) copolymer matrix. This matrix is a thermoplastic elastomer. Henceforth, these components will be referred to as HDPE, CC, CB and SP, with SP referring to the combined stabiliser pack. The fillers have very low aspect ratios, although the calcium carbonate particles are not uniform or homogeneous. More detailed descriptions of the physical parameters of the fillers are included in Supplementary Materials: Tables S1 and S2. This study follows on the work of Viljoen and Labuschagné [42] and makes use of the same materials and experimental design. With the exception of the CC, the upper limits of the additive loadings were set based on manufacturer recommendations. The upper limit of the CC loading was specified to the maximum that the authors felt would result in functional materials across the range of loadings, based on the existing literature. Even at its maximum loading of 60 wt% or 36 vol%, the CC falls well below even conservative estimates for maximum packing density, which fall in the range of 60 vol%. [47].

### 2.2. Formulation and Processing Methods

**Figure 1:**
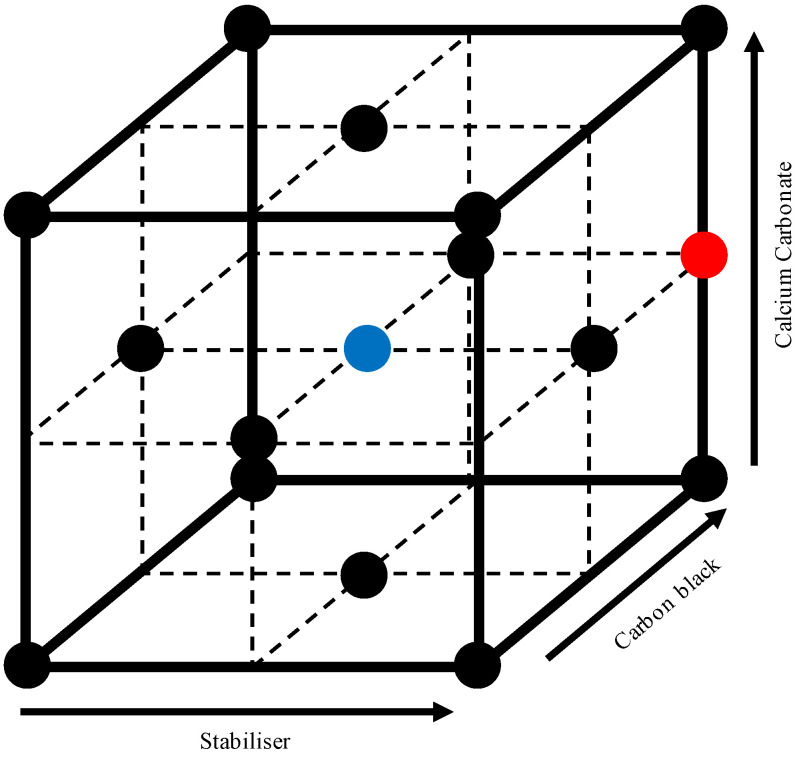
Slack-variable representation of experimental design. The blue dot is the duplicate centroid, while the red dot is the feed-varied duplicate set. Reproduced with permission from Viljoen and Labuschagné, Polymer Testing; published by Elsevier, 2020 [42].

The materials were formulated according to an I- and D-optimal experimental design with 18 points, with the design function a quadratic Scheffé-polynomial model. Three levels were used for each additive, with the extreme values being 0% and 3.6% for CB, 0% and 60% for CC and 0% and 0.5% for SP. Mid points were also used. Duplicate centroids were included in the experimental design, as were two points of a validation formulation—outside of the optimal design parameters. The only difference between the validation points was the CC side-feed used. These formulations are elucidated in Table 1. This is graphically illustrated in Figure 1, through the use of the slack-variable form [48].

The materials were compounded using a KraussMaffei Berstorff ZE 25-Cl with a suitably configured screw ( 1200 mm, 48 D) and a temperature ramp from 220 ${}^{\circ }$C to 235 ${}^{\circ }$C at the die. The HDPE, CB and SP were introduced through the main feed, while CC was introduced through one or both of the available side feeds. This is denoted in Table 1. In the case of the 60% CC materials, equal amounts of CC were introduced through each side feed. The side feeds were positioned at approximately 14D and 25D. After extrusion, the materials were cooled in a water bath, strand pelletised and allowed to cool. Prior to injection moulding, the granules were held overnight (at least) in vacuum ovens at 80 ${}^{\circ }$C to drive off as much moisture as possible.

Injection moulding was performed on an Arburg Allrounder 420 C 1000-250, with a melt temperature of 220 ${}^{\circ }$C, a mould temperature of 50 ${}^{\circ }$C and a cycle time of 30 s. Small adjustments were made to the pressure profiles of the formulations to ensure the production of high-quality specimens. These standard injection moulding conditions were used as a way of constraining the number of variables, while ensuring the production of high-quality specimens, with optimisation of processing conditions possible in a later work.

Tensile bars conforming to ISO 527-2 1BA [49] were prepared, with and without weld lines, with the lengthwise injection of the polymer melt. Drawings of the injection-moulding forms used are included in Supplementary Materials, Figure S1. Naturally, in the case of the WL samples, the melt was introduced from both sides of the specimens. Linear-decay packing was used, with an average nozzle pressure of about 700 bar.

### 2.3. Characterisation

#### 2.3.1. Tensile Testing

With the samples prepared, they were subjected to tensile testing in accordance with ISO 527 [49,50] on an Zwick/Roell UTS, equipped with a MultiXtens contact extensometer. A test speed of 1 mm/min was used to determine the modulus of the material (up to a strain of 0.25%), switching to a test speed of 50 mm/min until failure.

The DIC measurements were performed on the same machine, with the addition of an GOM ARAMIS 2D DIC system. As some of the specimens were white and some black, paints of the inverse colours were used for optimal contrast. Data analysis was performed in GOM ARAMIS v6.3.0. Only the two largest dimensions of the tensile bars were surveilled, with the x-axis in line with the direction of extension. A test speed of 5 mm/min was used throughout, as a result of the imaging frequency of 1 Hz.

Weld-line factors were calculated for each specimen, according to Equations (1) and (2). The data for WL and REF specimens were combined to allow for the retention of data points and, thereby, a better understanding to be gained over the distribution of behaviour and the presence of outliers, if any.

(1) $$\mathrm{WLF}\left(y_{i,j,k,n}^{WL}\right)=\frac{y_{p,q,r,n}^{WL}}{{\bar{y}}_{p,q,r}^{REF}}$$

(2) $$\mathrm{WLF}\left(y_{i,j,k,n}^{REF}\right)=\frac{{\bar{y}}_{p,q,r}^{WL}}{y_{p,q,r,n}^{REF}}$$

where *y* is the measurement of interest, *p* is the CB level, *q* is the CC level, *r* is the SP level and *n* is the specimen number.

Where relevant and applicable, a symbolic shorthand is used to ease identification of data series. A circle represents CB, a triangle CC and a square SP. The colour of the symbol indicates the level of the additive, with yellow a zero loading, indigo a maximum loading and green an intermediate loading. A filled symbol represents a REF specimen while a hollow symbol represents a WL specimen. Thereby, 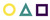 represents a WL specimen of a 0-CB/30-CC/0.5-SP material.

#### 2.3.2. Scanning Electron Microscopy

A Thermo Fischer Scientific Phenom XL was used for the analysis of the morphology of the failed specimens, through the use of its back-scattered electron detector and secondary-electron detector for material contrast and topography, respectively. Energy-dispersive X-ray spectroscopy analyses were trialled, but the failure surfaces were too uneven to yield accurate results. The specimens were prepared for analysis by slicing with a razor blade at ambient temperature, approximately 3 mm from the fracture surface. The specimens were mounted on the sample holder using adhesive carbon tape. Thereafter, the specimens were coated with a 3 nm platinum layer by sputter coating.

### 2.4. Fitting

After appropriate processing of the raw and output data, Scheffé polynomial models of up to quadratic degree (Equation (3)) [48] were fitted through an automated algorithm. Logarithmic (Equation (4)) variations on the standard Scheffé models were also trialled, as, in some cases, behaviour over multiple orders of magnitude may be observed. This type of behaviour is not well described by conventional Scheffé models operating in the linear space. These equations are subject to the limitation set out in Equation (5).

(3) $$\hat{y}=\sum_{i=1}^{p}{\hat{\beta }}_{i}x_{i}+\sum_{i=1}^{p}\sum_{j>i}^{p}{\hat{\beta }}_{ij}x_{ij}$$

(4) $$\log\hat{y}=\sum_{i=1}^{p}{\hat{\beta }}_{i}x_{i}+\sum_{i=1}^{p}\sum_{j>i}^{p}{\hat{\beta }}_{ij}x_{ij}$$

(5) $$1=\sum_{i=1}^{p}x_{i}$$

A major risk encountered in the fitting of models containing many terms to smaller datasets is that of overfitting. To screen—qualitatively—for this, two datasets were used for fitting. The first contains only the ideal points, with Formulation 1 and Formulation 2 excluded. The second contained the full experimental dataset. Through this, overfitting may be identified as major deviations between the predictions of the models fitted to the two datasets.

For the purpose of model selection, a number of metrics are used to assess the quality-of-fit of the models. These include their normalised mean absolute errors (nMAE, Equation (6)) and adjusted $R^{2}$ (Equation (10)) values [48]. Further, the F-statistics (Equation (11)) [48] and t-statistics (Equation (12)) (and the relevant *p*-values) of the models and their parameters were investigated. These may be used to judge the significance of the model and its parameters, and are important factors in the further discussion of the findings. It is important to note that the null hypothesis that many of these metrics are based on differs from what is used in conventional linear models, with the result that the equations differ somewhat from their normal form. This is due to the lack of a constant in the mixture model. The new null hypothesis, as proposed by Marquardt and Snee [48], can be structured as shown in Equations (13) and (14). This proposes the null model to be a constant model, with the best fit that of ${\hat{\beta }}_{i}=\beta_{0}=\bar{y}$.

(6) $$\mathrm{nMAE}=\left(\frac{\sum_{i=1}^{n}\left|y_{i}-{\hat{y}}_{i}\right|}{n}\right)\left(\frac{1}{\max y-\min y}\right)$$

(7) $$\mathrm{SSE}=\sum_{i=1}^{n}{\left(y_{i}-{\hat{y}}_{i}\right)}^{2}$$

(8) $$\mathrm{SSR}=\sum_{i=1}^{n}{\left({\hat{y}}_{i}-\bar{y}\right)}^{2}$$

(9) $$\mathrm{SST}=\sum_{i=1}^{n}{\left(y_{i}-\bar{y}\right)}^{2}$$

(10) $$\mathrm{adj}.R^{2}=1-\frac{\mathrm{SSE}}{\mathrm{SST}}\frac{n-1}{n-p}$$

(11) $$F=\frac{\mathrm{SSR}}{\mathrm{SSE}}\frac{n-p}{p-1}$$

(12) $$t_{{\hat{\beta }}_{i}}=\frac{{\hat{\beta }}_{i}-\beta_{0}}{s.e.({\hat{\beta }}_{i})}\;\mathrm{and}\;t_{{\hat{\beta }}_{ij}}=\frac{{\hat{\beta }}_{ij}-0}{s.e.({\hat{\beta }}_{ij})}$$

(13) $$\beta_{i}=\beta_{0}\;\mathrm{for}\;i=1,2,...,q$$

(14) $$\beta_{ij}=0\;\mathrm{for}\;i=1,2,...,q\;\mathrm{and}\;j=1,2,...,q$$

where ${\hat{y}}_{i}$ is the predicted value for $y_{i}$, $\bar{y}$ is the mean of *y*, β is a constant, *n* is the number of observations, *p* is the number of mixture components, SSE is the sum of squares error, SSR is the sum of squares regression and SST is the sum of squares total. $\beta_{0}$ is a constant—based on the null hypothesis—and s.e. is the standard error of a term.

From the work of McCuen, Leahy and Johnson [51], among many others, it is known that logarithmic transforms prior to the fitting of models results in—potentially substantial—bias in the predictions in the linear space. The more archaic methods of addressing this bias involve the alteration of fitted constants by some factor, typically with the aim of adjusting the intercept (in the case of power-law models). The method by McCuen, Leahy and Johnson, applied here, is the use of numerical fitting (nonlinear regression) to fit what is a nonlinear model in the linear space to the data, effectively minimising error and bias in that space. Where applicable, here, this is addressed by the fitting of equations of the form given in Equation (15) to the data in the linear space. This—as is shown in Supplementary Materials, Figure S6—effectively reduces bias, albeit not in all cases. Bias, here, is illustrated in a normalised form, as given by Equation (16).

(15) $$\hat{y}=10^{\sum_{i=1}^{p}{\hat{\beta }}_{i}x_{i}+\sum_{i=1}^{p}\sum_{j>i}^{p}{\hat{\beta }}_{ij}x_{ij}}$$

(16) $$\mathrm{nBias}=\frac{\sum_{i=1}^{n}{\hat{y}}_{i}-y_{i}}{\sum_{i=1}^{n}\left|{\hat{y}}_{i}-y_{i}\right|}$$

In the cases where nonlinear models are used—either though explicit fitting in the linear space or through fitting in the logarithmic space with conversion back to the linear space—it is well-established that measures such as adjusted $R^{2}$ fail due to the breach of their underlying assumptions [52,53]. The key assumption violated is given in Equation (17). This is investigated in more detail in Supplementary Materials, Figure S4, showing that low-error models approach the ideal behaviour needed to satisfy Equation (17).

(17) SST=SSE+SSR

As a large number of measures were available, the fitted results were evaluated for quality of fit and suitability. Through this, only models that offer significant and useful insights are further reported. In many cases, however, useful insights may be gleaned simply from the judicious use of data visualisation techniques.

## 3. Results and Discussion

### 3.1. Conventional Tensile Testing

#### 3.1.1. Young’s Modulus

**Figure 2:**
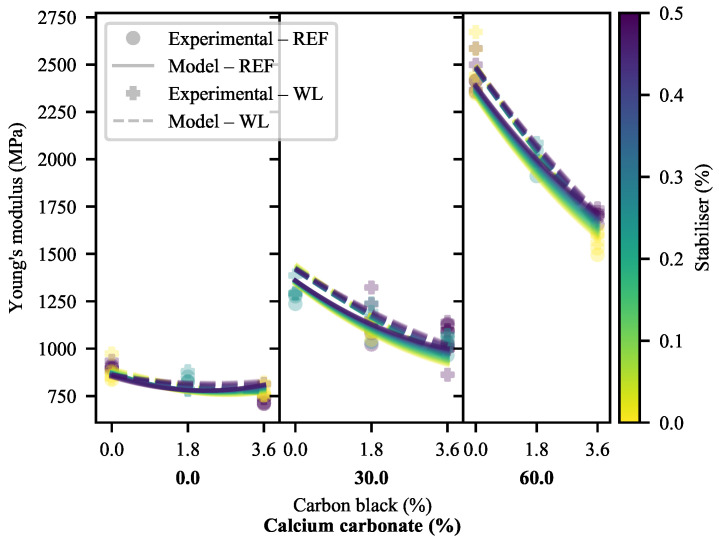
Influence of the specimen type and loadings of calcium carbonate, carbon black and the stabiliser package on the experimentally determined stiffness of the system with fitted models. Specific attention is drawn to the stepped increases in YM and the increasingly negative slopes as a function of CB with increasing CC.

The comparison of bulk material properties—Young’s modulus (YM), yield strength ($\sigma {}_{Y}$), elongation at yield ($\epsilon {}_{Y}$) and elongation at break ($\epsilon {}_{B}$)—offers a strong base upon which further evaluations can be based, and is thus the logical starting point. The raw experimental data are provided in Supplementary Materials, Table S3. From the experimental data for YM in Figure 2, it is clear that the most prominent effects on the stiffness of the system are those of CC and CB; CC with a positive and increasing effect and CB with a steady negative effect. Antagonism may be seen between the CC and the CB, based on the increasingly negative slope with increasing CC and CB. The presence/absence of stabiliser has very little direct effect. In this measure, it appears as though weld lines have a fairly insubstantial effect. However, the exact deviation is difficult to quantify based on the visual inspection of the raw data.

As is typical in this system, a quadratic model offers the best quality of fit. Here, specifically, the difference between a model of the form given in Equation (3) and that given by Equation (15) is marginal. By Occam’s razor, the linear-space model is preferred where the difference in quality of fit is marginal (adjusted $R^{2}$ values of 0.986 and 0.982 and nMAE values of 0.027 and 0.029 for the WL and REF models). The quality of fit can be verified visually, using the model predictions in Figure 2. At a significance level of 5%, typical in the modern scientific literature, the model fitted to the REF data possesses six statistically significant variables. In contrast, the model fitted to WL data only possesses three statistically significant variables. Common between them are the primary effects of HDPE and CC, and an antagonistic interaction between HDPE and CC (this results in the increasing slope with increasing CC loading). These data, along with the relevant fitted parameters and other metrics for all of the models used are available in Tables S4 and S5 of Supplementary Materials.

**Figure 3:**
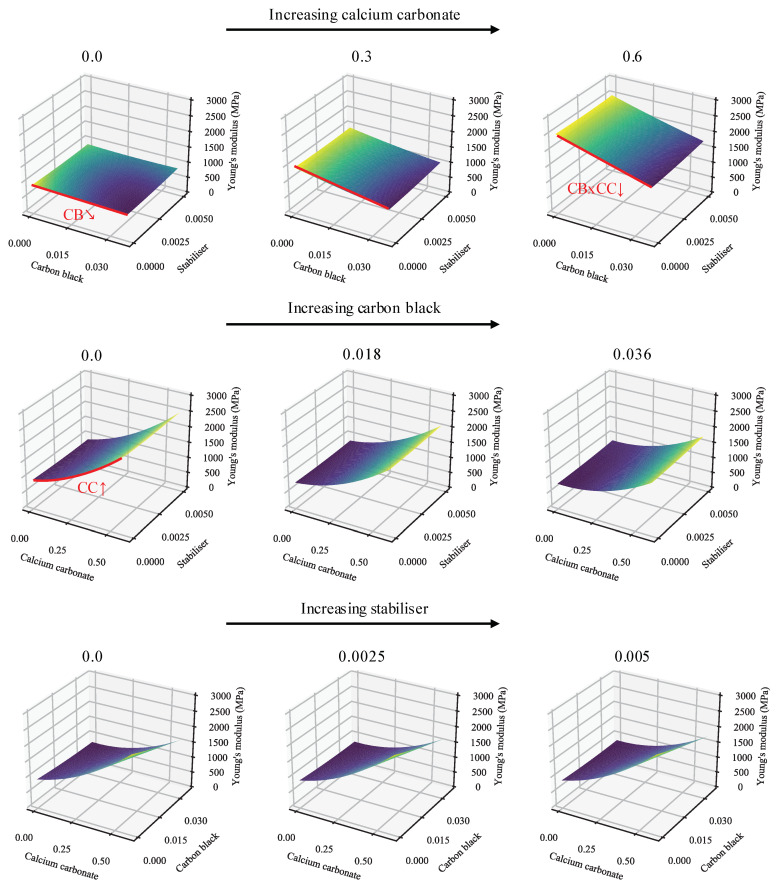
Weld-line Young’s modulus model predictions. Here, note particularly the very strong positive effect of increasing CC loadings, increasing with increasing CC loading, and the slightly negative primary effect of increasing CB loadings at 0-CC, becoming increasingly negative with greater CC loadings.

This is curious, as the primary and interactive effects of CB appear clear and significant, even from the raw data. Indeed, this can be explained. In systems of this sort, collinearity is rife and many of the variables are confounded (stemming from the effects of Equation (5)). To circumvent these problems and allow for the plain-language discussion of the primary and interactive effects, it is useful to make use of surface plots of the models. The full-effect surface plots for the WL and REF models are given in Figure 3 and Supplementary Materials, Figure S8. For the sake of brevity, only one example of each of the model plots is shown, with the rest relegated to Supplementary Materials. Most notable, here, is the similarity between the model predictions, also shown in Figure 2, despite the models having disparate fitted parameters. Further, it is clear that the effects and interactions discussed in the prior paragraphs are present. The minimal effect of variations in SP is also apparent. This, at least, can also be seen from the significance data—with the primary and interactive effects of SP consistently having the lowest significance values in the system. A comparison of the model plots in Figure 2 confirms the increase in stiffness brought about by the presence of weld lines.

**Figure 4:**
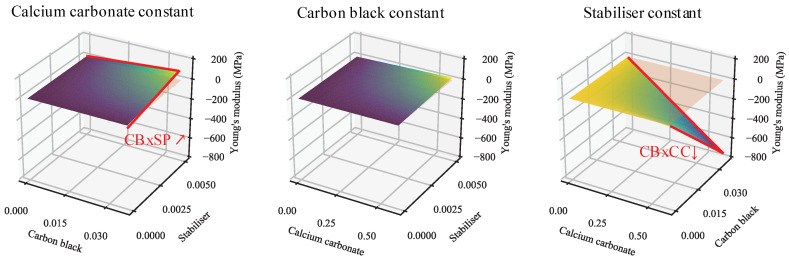
Weld-line Young’s modulus model predictions for interactive effects. Note the very strong antagonistic interaction between CB and CC, as well as the slightly positive interaction between CB and SP.

Next, interactive effects may be studied through the use of plots omitting the primary effects of variables (Figure 4 and Supplementary Materials, Figure S9). Again, the plots are startlingly similar, indicating minimal deviation in behaviour owing to the presence/absence of weld lines. Clear in both plots is the antagonism between CB and CC. Furthermore, visible—albeit far weaker—are CB/SP and CC/SP synergisms. These—as mentioned in the prior paragraph—possess very low significance. Their presence in both models, however, may serve in their favour and suggest that there are truly small interactive effects between these additives. This is more likely for CB/SP than CC/SP, as CB/SP is a stronger effect and is consistent across both models. Again notable is the difference in model terms in contrast to the very similar predictions offered.

Generally, the primary effects are to be expected. It is well known that increasing filler loadings (especially of high-stiffness fillers) generally increase the YM of a material as a result of increasing crystallinity [40,54,55] and stress concentration resulting from the presence of the heterogeneities [38].

Slightly more complex behaviour can be seen with the addition of CB. Here, it must be remembered that relatively little CB is introduced in an SEBS matrix, with the result that the effects of the SEBS dominate any stiffening effect that the CB may have. Keeping this in mind, even the addition of elastomers may result in complex effects—particularly when fillers are present. In a pure HDPE system, the addition of the softer SEBS to the matrix results in reduced YM, with the rate of reduction increasing with increasing loading. In filled systems, the addition of a maleated SEBS to an HDPE/organic-modified montmorillonite was found to first result in an increase in stiffness, followed by a decrease with increasing loading [56,57]. This suggests a compatibilising/exfoliating action [57] that reaches saturation before the usual softening effects take over. This exfoliating effect, of course, is absent from the materials being studied here. It may be reasoned that the addition of the softening elastomer will have a stronger effect on the stiffer materials, and this may be seen in the antagonism between CB and CC. This effect may be calculated to far outstrip the effects of the proportional increase of SEBS:HDPE at the higher filler loadings (Supplementary Materials, Figure S2).

**Figure 5:**
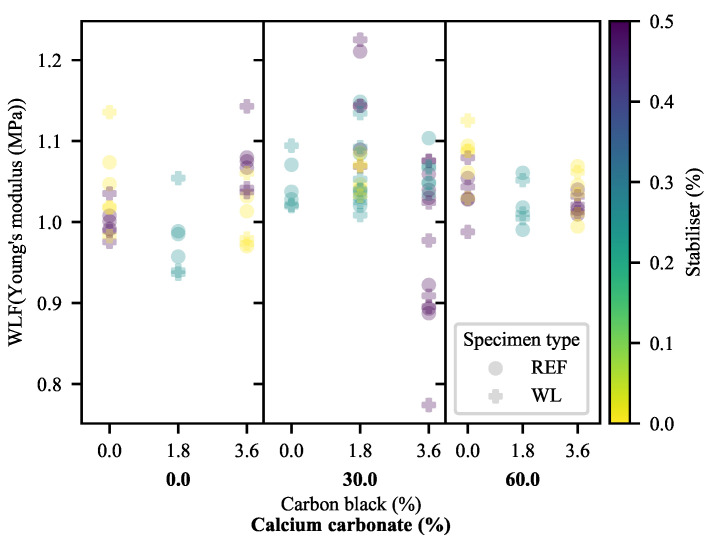
WLF(YM) as a function of the loadings of calcium carbonate, carbon black and the stabiliser package. Of particular interest, here, is the trend for the factors to be slightly above 1, virtually regardless of formulation.

The effects of weld lines on the stiffness of the system may be better investigated through the determination of the WLFs for each material (Figure 5). This confirms that the weld lines nearly universally increase the stiffness of a specimen. Only two exceptions may be identified: those of the 1.8-CB/0-CC/0.25-SP and 3.6-CB/30-CC/0-SP specimens. On the other hand, 1.8-CB/30-CC/0.5-SP specimens exhibited a larger increase in stiffness than that of comparable formulations in the presence of weld lines. In general, a slight curvature can be seen, suggesting moderate antagonism between CB and CC; no other definitive effects can be seen. No significant change was seen as a function of the feed point between Formulation 1 and Formulation 2.

The WLF(YM) of polyolefin specimens reported in the literature varies widely, with some precedent for WL specimens having higher YM than their REF counterparts—when appropriate materials and processing conditions are selected [58]. In that case, it was attributed to the HDPE having a bimodal molecular weight distribution, in addition to a high average molecular weight. In the present work, the HDPE used is believed to share the high molecular weight [59] of the material in the work of Tjäder, Seppälä and Jääskeläinen [58], but its molecular weight distribution is unimodal. The HDPE at hand was specifically developed for injection-moulding applications, with a focus on mechanical properties and ready processability [60]. The compatibility of the materials is known to have an impact on the WLF(YM) of composites, with increasing compatibility resulting in increased WLF(YM) (although no cases where found where these exceeded one).

The consistently higher stiffness of the WL specimens, however, suggest a fundamental change. This is hypothesised to stem from the the morphological changes that result from the effectively halved linear injection speed—but similar injection time—of the two-sided flow, coupled to the reduction in distance from the gate [61,62,63].

#### 3.1.2. Yield Strength

**Figure 6:**
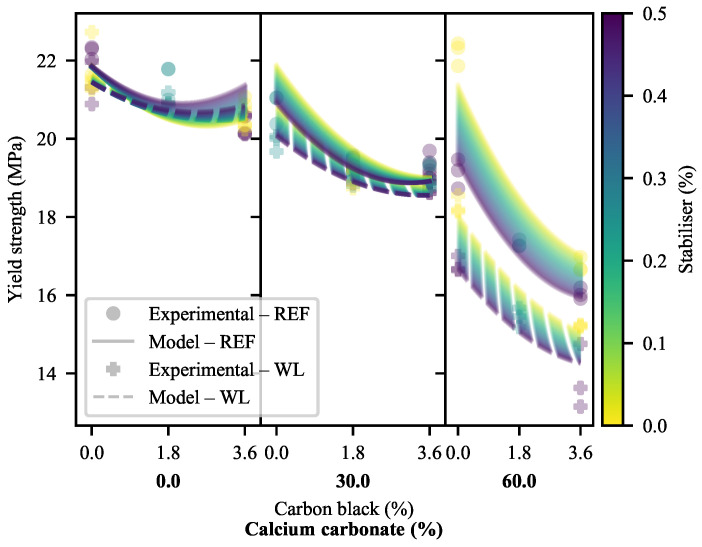
Influence of the specimen type and loadings of calcium carbonate, carbon black and the stabiliser package on the yield strength of the system. Specific attention must be paid to the similarity of the curves, with the distinct exception for those at the 60-CC level. Furthermore, note the exceptional performance of the 0-CB/60-CC/0-SP material.

The effects of the additives on the yield strength ($\sigma {}_{Y}$) of the materials (Figure 6) offer no great surprises at lower loadings of CC. An increase in CC results in a reduction in $\sigma {}_{Y}$, as does an increase in CB (albeit with a slope decreasing with increasing CB loading). Then, clearly, a substantial change occurs in the behaviour of the system at a 60% loading of CC, with a much sharper decline in $\sigma {}_{Y}$ with increasing CB and an increased sensitivity to weld lines compared to that seen at other loadings. This is accompanied by transition of failure from ductile to brittle. Here, too, can be seen a dramatic increase in $\sigma {}_{Y}$ for a 60% CC/0% CB compound without SP as compared to an equivalent with SP. It must be noted, here, that the ultimate tensile strengths of the 60-CC materials were used as their yield strengths for the purposes of comparison and modelling, due to their brittle failure.

Through the use of a quadratic model, again in the linear space, these findings may be clarified. The negative primary effects of CB and CC are confirmed, in addition to the minimal primary effect of the stabiliser package. An increasing level of change with increasing CC is found in the WL model, as suggested in Figure 6. The interactive effects are uniform across the WL and REF models, with synergism between CB and SP, contrasted by antagonism between CB/CC and CC/SP.

It must be noted that—although the model adequately describes the experimental data points—it is unlikely that the change between 0-CB/60-CC/0-SP and 0-CB/60-CC/0.5-SP will be smooth. Instead, once a certain value of SP (less than 0.5%, likely less than 0.25%) is exceeded, behaviour will revert to what is seen elsewhere—with minimal effects brought about by changes in SP regardless of the levels of other additives. This is expected, as the increase in $\sigma {}_{Y}$ seen is ascribed to the effects of degradation-driven crosslinking in the matrix [42,59,64,65]. The given level of SP will mitigate this effect and offer little further change [66].

A decrease in $\sigma {}_{Y}$ with increasing filler loading (in the absence of other additives) is not atypical, but the extent of this change is very much dependent on the material properties, ranging from particle size/surface area to the compatibility of the matrix with the fillers. A decrease in a fairly strong matrix seems natural, as the presence of fillers result in the decrease of the effective cross-sectional area of a specimen, although this is offset by interfacial interactions stemming from compatibility of an interphase [38]. The network of filler particles with interconnected shells [40] discussed in the introduction is likely to play some role here. It was found that local maxima for performance may be achieved at around 50% CC, with a sharp drop-off at higher loadings. This loading results in the optimal ligament thickness parameters, with the local maxima resulting. Owing to the fairly fine particle size and compatibilisation of the CC used in the present study, the reduction in yield strength is present but quite linear from 0% CC to 60% CC.

Furthermore, typical is the reduction in yield strength of the 0-CC formulations with increasing CB, as a result of the softening effect of the SEBS [56,57]. Notable, however, is the strength of the apparent antagonism between CB and CC. Typically, the addition of an elastomer results in a much smaller reduction of the yield strength of a composite than it would in that of the virgin matrix [57]. This finding is usually based on much lower loadings of fillers than seen here, with those fillers frequently not highly compatibilised with the matrix. Here, the causes of the effect are believed to be multi-fold. The primary cause is the increase in the proportion of the elastomer relative to the matrix (Supplementary Materials, Figure S3), resulting in a more prominent reduction in strength. This, however, can be calculated to exceed by some margin the apparent degree of antagonism. Thereby, the addition of the elastomer is expected to enhance the mobility of the matrix chains, somewhat countering the proportional increase in CB loading and its accompanying negative primary effect. In such a reference framework based on the amount of matrix rather than total material, the reduction in rate of decline seen in the 30-CC materials in Figure 6 is emphasised—suggesting the effects of an otherwise unseen change in deformation mechanics. This stands in contrast with the curvature found by Sahnoune, Lopez-Cuesta and Crespy [67], despite their finding that the compatibilisation of CC in HDPE with SEBS had a predominantly negative effect on the yield strength of the materials. The curvature of their findings, however, is opposite to that in the present work, with their reported yield strengths decreasing faster with increasing SEBS loading.

**Figure 7:**
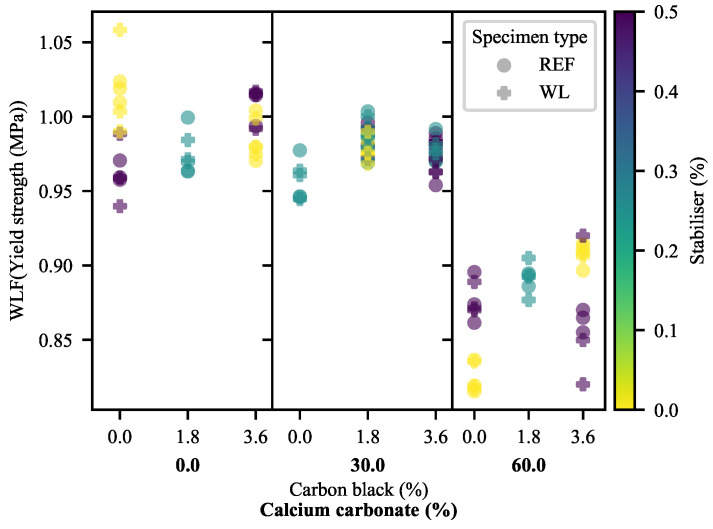
WLF($\sigma {}_{Y}$) as a function of the loadings of calcium carbonate, carbon black and the stabiliser package. Specific attention must be paid to the step down to the 60-CC level and the curvature of the trends as a function of CB.

If the WLFs of this measure are interrogated (Figure 7), it is clear that weld lines have a near uniformly negative effect on the $\sigma {}_{Y}$ of the material, worsening with higher loadings of CC. CB appears to have a positive (albeit small) primary effect. SP has variable effects, with a strong dependency on the presence of CB and/or CC. In 0-CB/0-CC and 3.6-CB/60-CC materials, it has a weakening effect. This is inverted for 3.6-CB/0-CC and 0-CB/60-CC materials. This appears to be a ternary antagonism, but, unfortunately, ternary interactions exceed what can be reasonably modelled using the data at hand. As a result, modelling was omitted here. It does, however, appear as though the diverting behaviour of 0-CB/60-CC/0-SP continues, with it likely that the cross-linked structure discussed earlier is resulting in the reduced performance. This, likely, is due to the localisation of the cross-linked species on either side of the weld line, with minimal bridging of cross-linked chains. Given that these species have lower mobility than their cross-link-free equivalents, interdiffusion is likely to be compromised [68].

The literature would suggest that higher filler loadings result in a decrease of the WLF$\left(\sigma {}_{Y}\right)$ of materials [10,38,69,70,71]. It must be noted, however, that a large portion of the results cited in the literature are solely focused on anisotropic fillers. Even outside of these, the reduced chain mobility brought about by the presence of the filler particles will invariably reduce the rate (and overall level) of interdiffusion, thereby compromising the integrity of the material—clearly seen here. The extreme case can be seen in the 60-CC cases, where the REF specimens already exhibit brittle fracture but the WL specimens have notably decreased yield strengths in comparison.

The improvement in WLF$\left(\sigma {}_{Y}\right)$ brought about by the addition of an elastomer to a polymer matrix has substantial precedent in the literature, albeit typically in polycarbonate or polyethylene terephthalate systems [72]. While the step-down in WLF$\left(\sigma {}_{Y}\right)$ at the 60% CC level may be explained by the reduced interdiffusion/entanglement caused by the high CC loading, the upward trend seen even here with increasing CB loading is evidence of the positive effects of the addition of SEBS on the WLFs of these materials. This, likely, stems from enhanced interdiffusion, owing to the reductions in viscosity that accompany the addition of SEBS to a system of this type [73]. Furthermore, notable is the curvature in behaviour, suggesting that the softening effect of the elastomer becomes dominant in the vicinity of a 1.8% CB loading in filled materials. The appearance of this is to be expected, as it has been established that “less ideal” components (and even sections of polymer chains) are rejected from the more ordered and preferentially crystallised regions [55]. Given that the core of the material is less ordered than the skin, and that a v-notch typically bisects the skin, the softening effect of the SEBS may be more apparent. It is also likely that some complex interactions are present between CB and SP, particularly at 0% and 60% CC loadings.

Mielewski et al. [74] found that polypropylene samples with low loadings of antioxidants (in their study, phenolic), exhibited the concentration of these antioxidants at weld lines. This, naturally, was found to hinder the entanglement of the polymers from the two sides of the weld lines, resulting in locally decreased mechanical properties. As the antioxidants used in this work are similarly incompatible with the matrix, similar effects may be expected to be present, at least in some of the cases.

#### 3.1.3. Elongation at Yield

**Figure 8:**
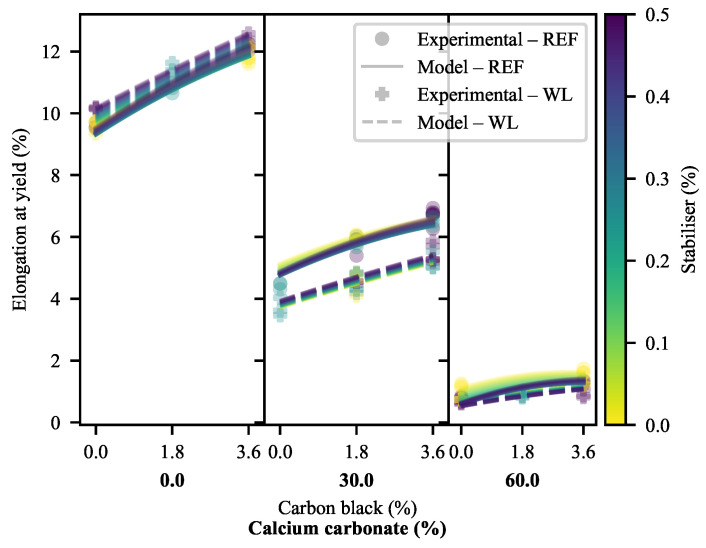
Elongation at yield as a function of specimen type and the loadings of calcium carbonate, carbon black and the stabiliser package. Note the similarity in curvature between the WL and REF cases and the step at the 30-CC level.

**Figure 9:**
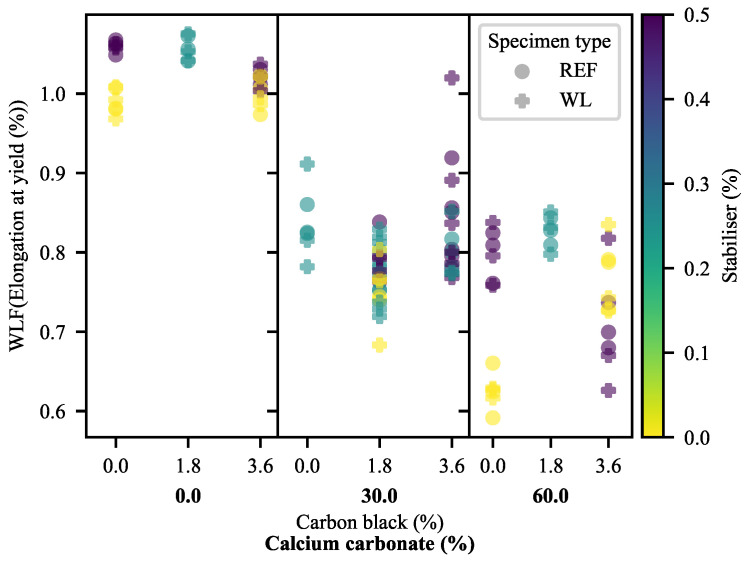
WLF($\epsilon {}_{Y}$) as a function of the loadings of calcium carbonate, carbon black and the stabiliser package. Distinct changes in curvature with changing CB can be seen with the steps in CC, as well as the step-down from the 0-CC level.

The first evidence of substantial detrimental effects of weld lines on the 30-CC materials may be seen in the $\epsilon {}_{Y}$ of the materials (Figure 8 and Figure 9). As may be anticipated, $\epsilon {}_{Y}$ mirrors the results of YM in most of the formulations, albeit with some deviation owing to the variable $\sigma {}_{Y}$ of the materials. That is to say that the materials exhibiting higher values for YM typically exhibit less $\epsilon {}_{Y}$ than materials with lower values for YM. In fact, materials without calcium carbonate exhibit (generally) greater $\epsilon {}_{Y}$ in the presence of weld lines. At the same time, 60-CC materials exhibit a step-down that may be anticipated based on their reduced $\sigma {}_{Y}$. Counter to this, the materials filled with 30% CC exhibit more extreme reductions in $\epsilon {}_{Y}$ as a result of weld lines than can be ascribed to the effects of YM and $\sigma {}_{Y}$. This suggests that the mid-to-late-stage pre-yield behaviour of WL specimens differs from that of REF specimens in the 30-CC formulations. In the deformation mechanism proposed by the Strobl group [16,17,18,19,20,21], this would suggest that nucleated/localised inter- and intralamellar slip stage has seen some type of change. Further evidence of complex interactions can be seen in Figure 9, through the changes in curvature as a function of CB loading across the range of CC loadings.

**Figure 10:**
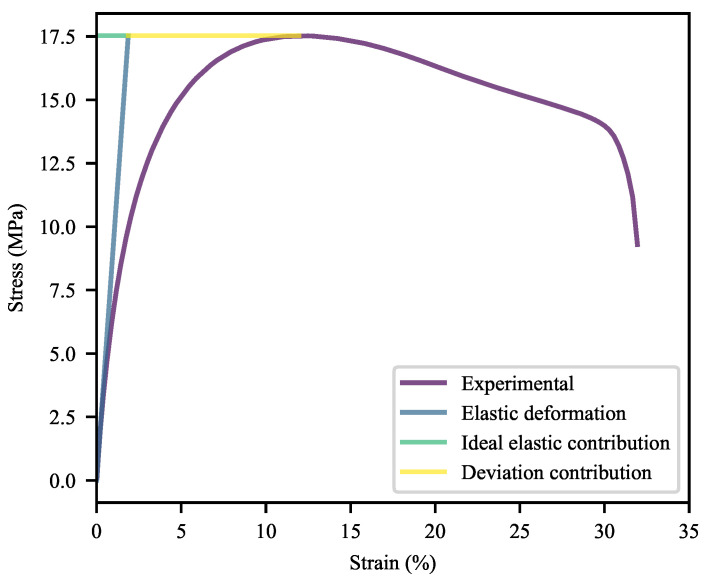
Determination of a deviation contribution, based on a stress-strain curve of a REF specimen of Formulation 1 under the DIC test conditions.

The deviation of the system from ideal elastic deformation may be better investigated through the use of a factor—termed deviation contribution (Equation (18))—and its fractional form (Equation (19)). Equation (18) is used to determine the size of the deviation of $\epsilon {}_{Y}$ from what would be expected assuming ideal elastic deformation and the relevant yield point. This is shown in Figure 10, for example. In terms of observable behaviour, this deviation is a combined effect—stemming from the shape of the inelastic curvature prior to yield and the positioning of the yield point itself. Equation (19) builds on this by calculating the fraction of $\epsilon {}_{Y}$ that results from this deviation. Due to the range of moduli exhibited by the materials, the fractional form allows for much more direct comparability. As with the other measures investigated here, WLFs may be calculated. The shrewd reader will note that the conventional tensile tests were conducted at two rates: 1 mm/min for the determination of YM and 50 mm/min for the other measures. To circumvent this, a power-law equation was fitted to the initial sections of the data acquired at 50 mm/min (with excellent fits achieved). Given that purely elastic deformation is virtually absent from the results, a power-law fit was deemed most appropriate and has a basis in the established literature [75]. Alternative moduli were calculated from these fits for use in this part of the investigation. These results were compared to the results obtained from the single-rate tensile tests performed for the DIC study, with excellent correlation found.

(18) $$\mathrm{DC}=\epsilon {}_{Y}-\frac{\sigma {}_{Y}}{YM}$$

(19) $$\mathrm{DF}=\frac{\mathrm{DC}}{\epsilon_{Y}}$$

where DC is the deviation contribution and DF is the deviation contribution factor.

**Figure 11:**
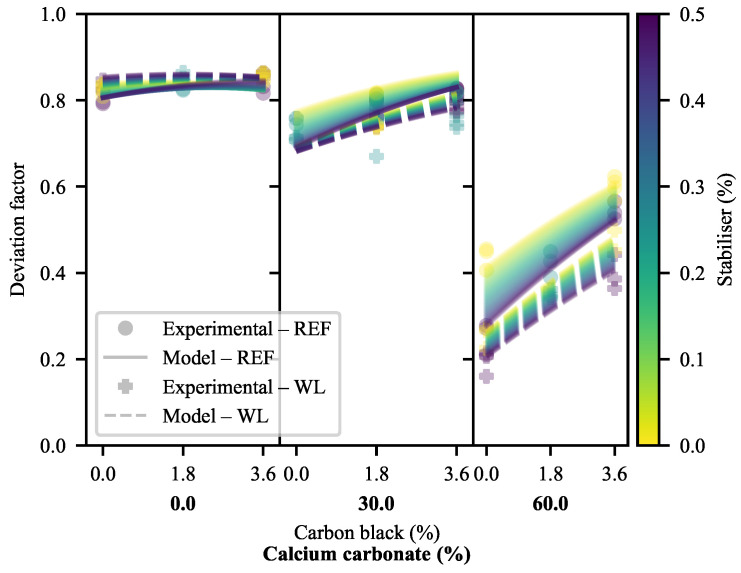
Influence of the loadings of calcium carbonate, carbon black and the stabiliser package on the DFs of the system, with fitted models. Note the sharper slopes of the REF cases and the switch-over from 0-CC to 60-CC.

Based on Figure 11, it is clear that the majority of $\epsilon {}_{Y}$ stems from deviation behaviour in most cases. It may also be seen that the greater $\epsilon {}_{Y}$ of 0-CC WL specimens compared to that of their REF equivalents stems from this region, while CB has a much lesser effect on the behaviour of WL specimens in this region than it has on equivalent REF specimens. This serves as further evidence that there are complex interactions between the additives and weld lines, particularly at higher loadings of CC. The positive impact of CB is clearly visible, as is the negative impact of CC. At the same time, SP has a more pronounced detrimental effect on the REF specimens than it has on the WL specimens (worsening with increasing CC).

**Figure 12:**
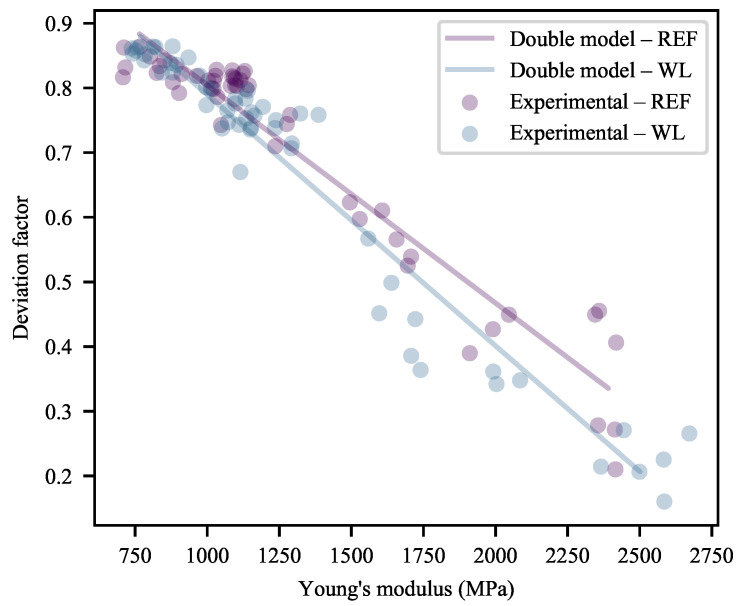
Linear correlation between DF and YM.

Keeping in mind that these data have already been partially corrected for YM through Equation (18), it is notable that there still exists substantial correlation between DF and YM (Figure 12). There is a distinct difference in the slopes of the correlations—depending on whether weld lines are present or not—suggesting a shift in material behaviour as a function of the weld line presence prior to the yield point. This difference in slope may be calculated to be statistically significant, with a *p*-value of 0.0035 if the model is built as shown—using the experimental YM data—and a *p*-value of <0.0001 when using the calculated YM data for a 50 mm/min extension rate.

Referring back to Figure 12, it is appears as though the majority of the experimental correlations are well described by linear fits. However, it stands to reason that the maximum value of DF is 1. Based only on the trends seen here, this asymptote will be reached at a YM of 700 MPa. It may, therefore, be reasoned that as the line approaches the asymptote it will decrease in slope, eventually meeting it at the hypothetical YM of 0 MPa. By the same logic, the minimum value of DF is 0 at some maximum YM. Therefore, sigmoidal behaviour is expected. Physically, the behaviour as DF approaches 1 tends to that of a ideal viscous liquid. Conversely, as DF approaches 0, the behaviour of the material approaches that of an ideal elastic solid. Indeed, some traces of what appears to be sigmoidal behaviour can be seen in Figure 12, particularly for the WL specimens.

**Figure 13:**
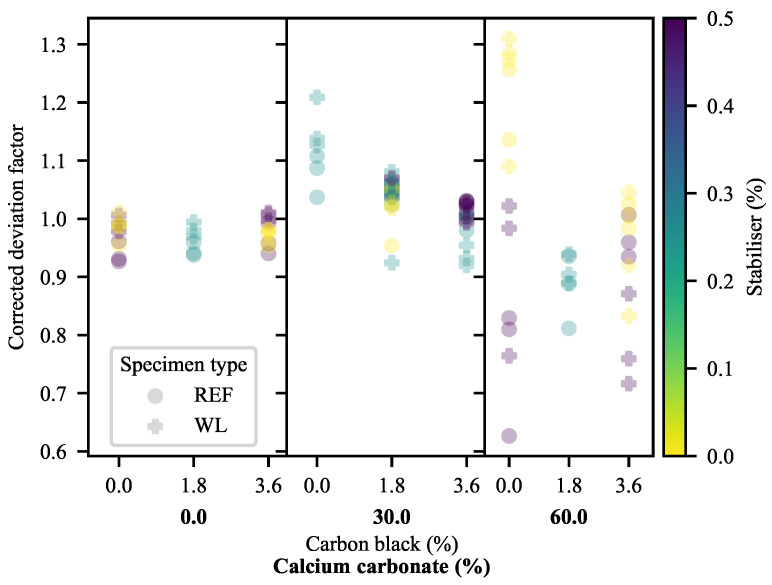
Corrected deviation factor as a function of the loadings of calcium carbonate, carbon black and the stabiliser package. Note the near-independence of the results on additive loading.

If, within this linear region of study, the DF is corrected to eliminate the remaining effects of YM by division according to Equations (20) and (21), Figure 13 results. This correction is done according to the linear equation fitted to each specimen type in Figure 12, to allow better comparison of the relative effects.

(20) $$\mathrm{cDF}=\frac{\mathrm{DF}}{{\hat{\mathrm{DF}}}_{YM}}$$

(21) $${\hat{\mathrm{DF}}}_{YM}=f\left({\hat{y}}_{YM}\right)$$

where cDF is the corrected form of DF and ${\hat{\mathrm{DF}}}_{YM}$ is the model-predicted DF based on the linear functions shown in Figure 12 and the equations used to fit YM in Figure 2. These values were calculated separately for WL and REF specimens.

What is learned from the cDF in Figure 13 (and the weak quality-of-fit metrics of the models of this measure in Supplementary Materials, Tables S4 and S5) is that the effects of the additives are fairly well described by the linear correlations. It appears as though some behaviour related to CB is left unaccounted for at a 30% CC loading in REF specimens and that the strange behaviour of 0-CB/60-CC/0-SP relative to 0-CB/60-CC/0.5-SP persists. This suggests a slight deviation in the deformation behaviour at the 30% CC loading, while re-emphasising the odd behaviour of the 0-CB/60-CC/0-SP specimens. This, also, suggests that the trend for the divergence of the WL and REF specimens seen in Figure 12 holds true across the full range of CC loadings.

#### 3.1.4. Elongation at Break

**Figure 14:**
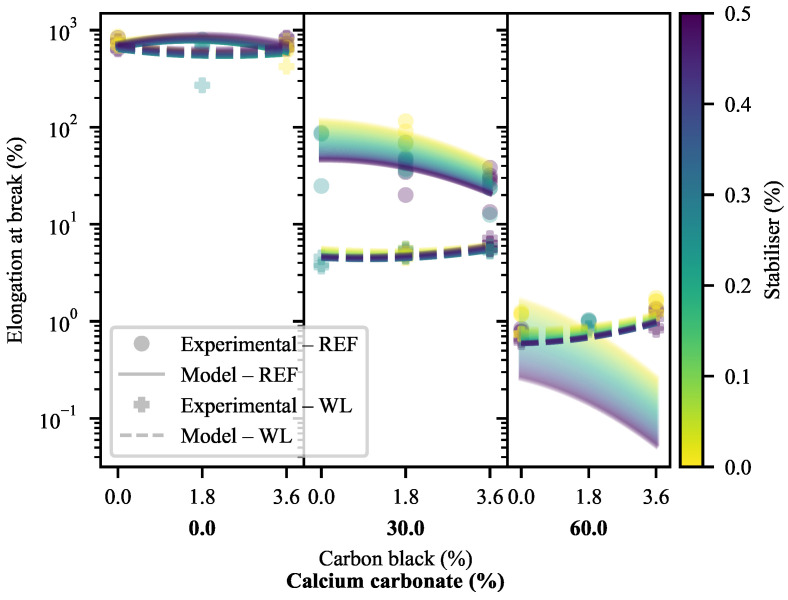
Change in elongation at break, as a function of the loadings of calcium carbonate, carbon black and the stabiliser package. Note the quality of fit of the models, with the clear exception of the REF model at the 60-CC level. Note also the step and contrasting slopes between the WL and REF cases at the 30-CC level.

The $\epsilon {}_{B}$ of the materials exhibits some interesting deviations from the results found for $\epsilon {}_{Y}$, particularly in the 30-CC region (Figure 14). It must be noted that a log-scale is used here. The expected reductions in $\epsilon {}_{B}$ coinciding with increasing CC are clearly visible. The same can be said of the increase in $\epsilon {}_{B}$ with increasing CB, albeit only in the WL 30-CC specimens and the 60-CC materials. The decrease in $\epsilon {}_{B}$ in 30-CC REF specimens with increasing CB is notable, as is the near absence of change in the 0-CC specimens. In the latter case, much of the change may be thought to be hidden by the change to log-scale, but there exists little change even in the linear scale. The exception is the on WL specimen of 1.8-CB/0-CC, but this is thought to be an outlier. In terms of the model predictions, it can be seen that the exponential model fitted in the linear space offers a near-perfect representation of the behaviour of the WL specimens. On the other hand, it cannot handle the inversion of the trend with increasing CB across the CC loadings in the REF specimens, as this would require a more complex model (with, at least, ternary interactions). As a result of the fitting method, precedence is given to the 30-CC points due to their larger linear-space variability—resulting in the compromised 60-CC fit.

It must be kept in mind that the 30-CC WL specimens and the 60-CC formulations failed at or very shortly after their yield points, with much of their behaviour explained by the factors discussed in the prior paragraphs and the DIC section. As discussed in the DIC section, the 30-CC specimens failed shortly after showing stress whitening on the weld lines, suggesting that the final disentanglement/pull-out stage set on and concluded very quickly. At the same time, the 60-CC specimens exhibited brittle failure. Here, it is worth noting that some of the 60-CC WL specimens did not fail on their weld lines during conventional tensile testing, but failure consistently occurred there during the DIC tests.

The decrease in $\epsilon {}_{B}$ with increasing CB in the 30-CC REF specimens is particularly interesting, as it goes against much of the present literature [56,57,76] concerning the effects of the addition of SEBS on the $\epsilon {}_{B}$ of virgin matrices, blends and composites. Indeed, the effects of CB on $\epsilon {}_{B}$ in HDPE vary based on particle size, compatibility and the nature of the matrix, among others, with the result that some studies report increases in $\epsilon {}_{B}$ with increasing CB [77,78] while others report the opposite [44]. An increase in $\epsilon {}_{B}$ with increasing CB may also be seen in a blend composite of linear low-density polyethylene with a polyolefin elastomer, CC and compatibilisers (including stearic acid) [79]. Thereby, this effect must be the result of some otherwise unseen interaction of CB affecting the onset of fibrillation and/or chain disentanglement/pull-out, as it cannot be seen in any of the measures wherein the other steps reveal themselves. This is discussed in more depth in the DIC section.

Strain hardening may be seen at high elongations in the WL and REF specimens of 0-CC materials, with stress sometimes exceeding $\sigma {}_{Y}$, but this behaviour is very inconsistent—with the result that this is omitted from further discussion.

#### 3.1.5. Summary: Conventional Tensile Testing

The primary and interactive effects present as a result of formulation variables and the presence/absence of weld lines may be summarised as in Table 2. Complex effects may be seen in numerous cases, frequently as a result of CC loading and/or the presence of weld lines.

### 3.2. Digital Image Correlation

To better understand the complex behaviour seen in several of the conventional measures, it is informative to investigate and compare materials in terms of their local deformation as a function of position and time. Two stages in the deformation of the materials are used as baseline for comparison: the stage at which the maximum force is applied (equivalent to the yield point) and the last stage at which usable data were collected.

#### 3.2.1. Deformation at Yield

**Figure 15:**
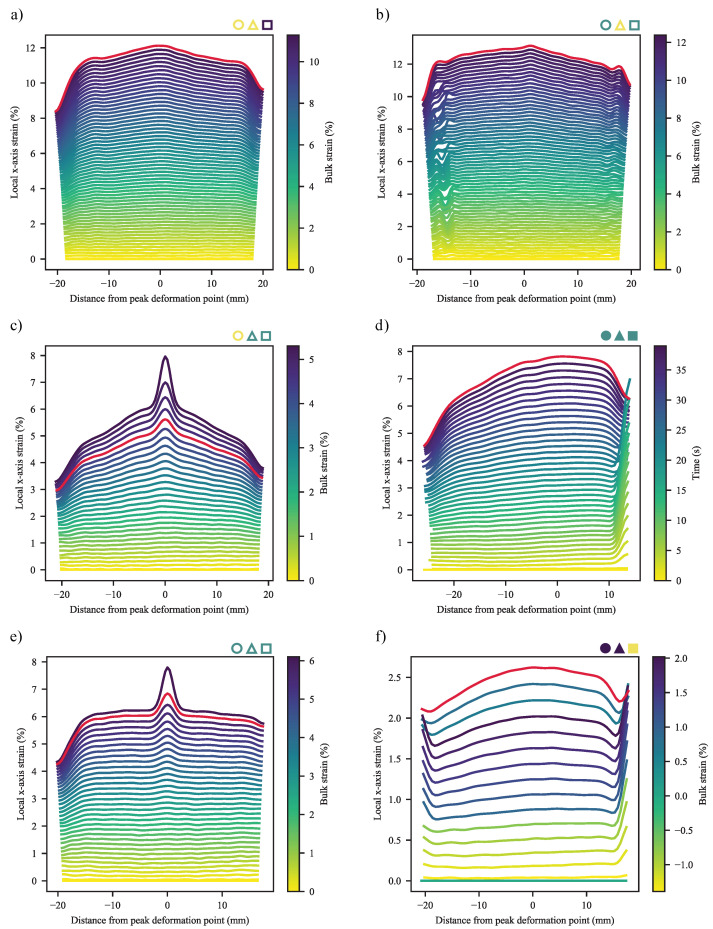
Time-series deformation profiles: yield. (**a**) 0-CB/0-CC/0.5-SP, WL; (**b**) 1.8-CB/0-CC/0.25-SP, WL; (**c**) 0-CB/30-CC/0.25-SP, WL; (**d**) 1.8-CB/30-CC/0.25-SP, REF; (**e**) 1.8-CB/30-CC/0.25-SP, WL; (**f**) 3.6-CB/60-CC/0-SP, REF. For the sake of brevity, complete profiles are shown in suitable cases. The yield profiles are highlighted in red.

Given the widely different deformation behaviours seen across the materials and specimen types, it is ideal to start with the behaviour of the materials up to their yield point. Exemplary deformation profiles are shown in Figure 15, ranging from the smooth, bulk deformation of mid/zero-CC REF specimens; broad-peak deformation of zero-CB, zero-CC WL specimens; gradual narrow-peak deformation of CB, zero-CC WL specimens; narrow-peak deformation of mid-CC WL specimens to the varied profiles of high-CC specimens. In some cases, that of WL 1.8-CB/0-CC/0.25-SP and 0-CB/30-CC/0.25-SP, combinations of broad- and narrow-peak deformations may be seen. For the sake of brevity, the complete time-series of deformation profiles are shown in figures where failure followed soon after yield, allowing clear interpretation of the profile at yield, with the yield profile highlighted in red. Further complete profiles are shown in the relevant section. In the plot for the REF specimen of 1.8-CB/30-CC/0.25-SP, the colour-bar axis was changed to time, as the inconsistent extremities (visible from the jagged edges of the plot) resulted in inconsistent measures for bulk strain.

**Figure 16:**
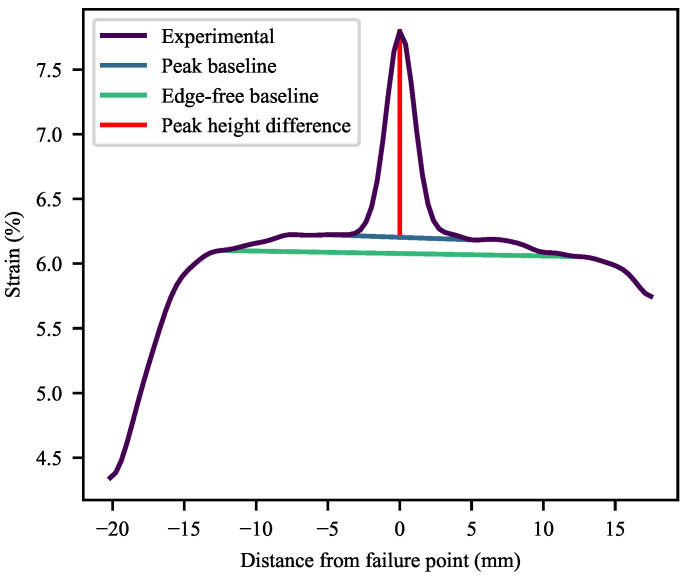
Example fitting, 1.8-CB/30-CC/0.25-SP WL. Fit on the yield time-step. The fitted parameters are used in Equations (22) and (23).

To quantitatively compare the deformation profiles, a number of measures were created. These make use of extensive data processing, based on the raw data and a numerical second derivative, to determine inflection points (Figure 16). Due to a typical symmetry, these inflection points may be linked through the fitting of linear equations to create different regions in the deformation plots. Based on the same technique, the edge-effects of the specimens may be identified and removed from certain calculations.

**Figure 17:**
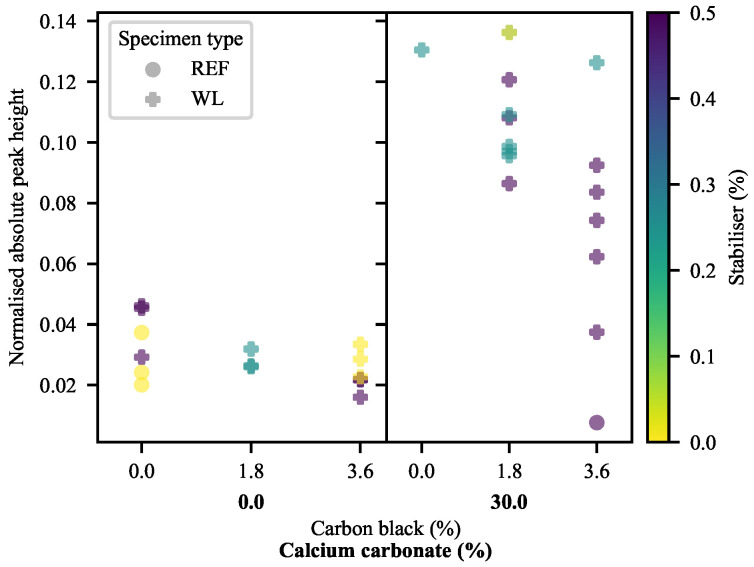
Peak strains relative to the mean strain of the base of the peak as a function of specimen type and CC, CB and SP loadings.

The simplest of the measures is a comparison of the maximum difference between the height of a peak and that of its base, divided by the mean strain of linear equation fitted to the base (Equation (22) and Figure 16). The results of this are shown in Figure 17, from where it is clear that the peaks become proportionally higher with an increase in CC, while an increase in CB typically results in the decrease of this measure. It was not possible to achieve repeatable DIC results for the 60-CC materials in this work, possibly due to insufficient time and spatial resolution.

(22) $$\mathrm{nAPH}=\frac{\Delta h_{p}}{{\bar{h}}_{pb}}$$

where nAPH is the normalised absolute peak height, $\Delta h_{p}$ is the peak height difference and ${\bar{h}}_{pb}$ is the mean height of the peak baseline.

**Figure 18:**
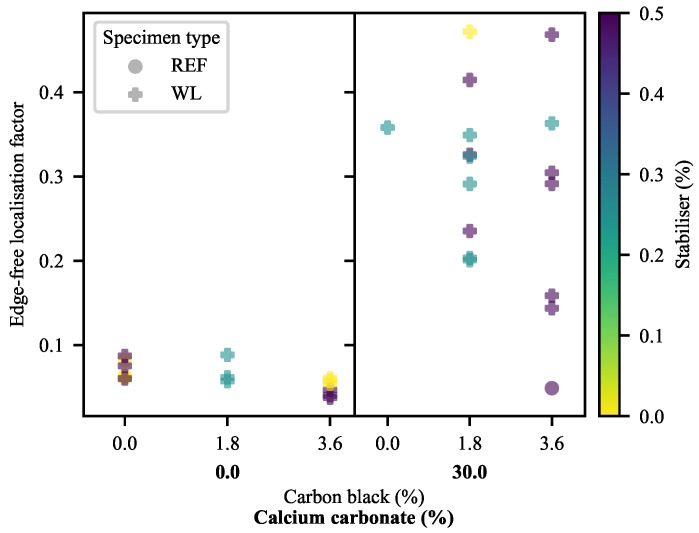
Extent of peak localisation as a function of specimen type and CC, CB and SP loadings.

This, however, only serves to indicate the extent of the local deformation, not how localised it is. To address that, the second measure is an edge-free localisation factor (EFLF) (Equation (23)). It is important to minimise or eliminate the edge-effects here, as these may muddle the effects seen. The larger the EFLF, the more localised the deformation. Thereby, it comes as no surprise (Figure 18) that the WL specimens exhibit larger EFLFs than their REF equivalents (given that the majority of these do not exhibit a noticeable peak). Of these, the 30-CC materials exhibit generally greater EFLFs than the zero-CC materials. This was noted qualitatively in Figure 15, where a distinct difference in behaviour can be seen between the broad peaks of the zero-CC WL specimens and the narrow peaks of their 30-CC equivalents.

(23) $$\mathrm{EFLF}=\mathrm{nAPH}\frac{w_{efb}}{w_{pb}}=\frac{\Delta h_{p}}{{\bar{h}}_{pb}}\frac{w_{efb}}{w_{pb}}$$

where $w_{efb}$ is the width of the edge-free baseline and $w_{pb}$ is the width of the peak baseline.

**Figure 19:**
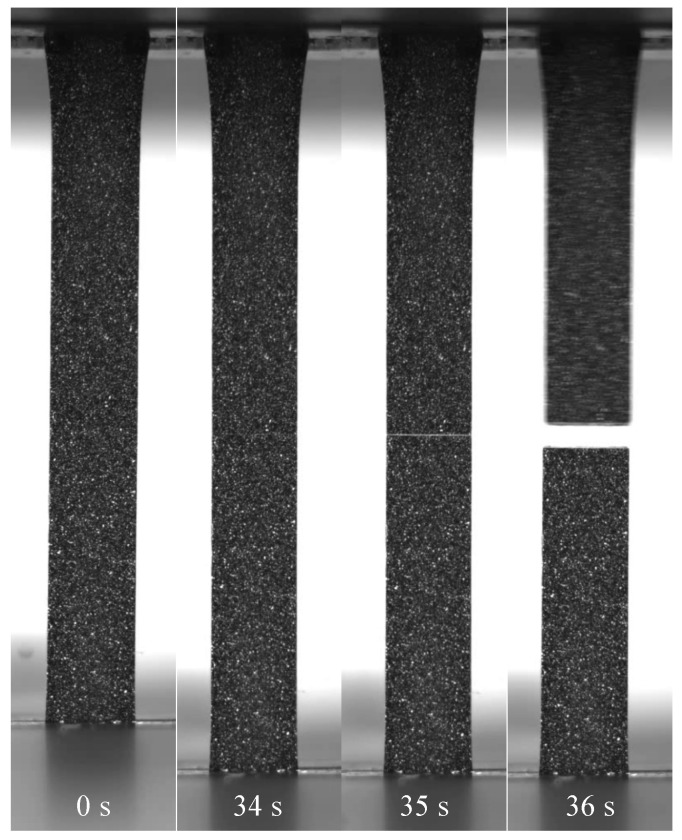
Deformation progression of a WL specimen of 1.8-CB/30-CC/0.25-SP under DIC.

**Figure 20:**
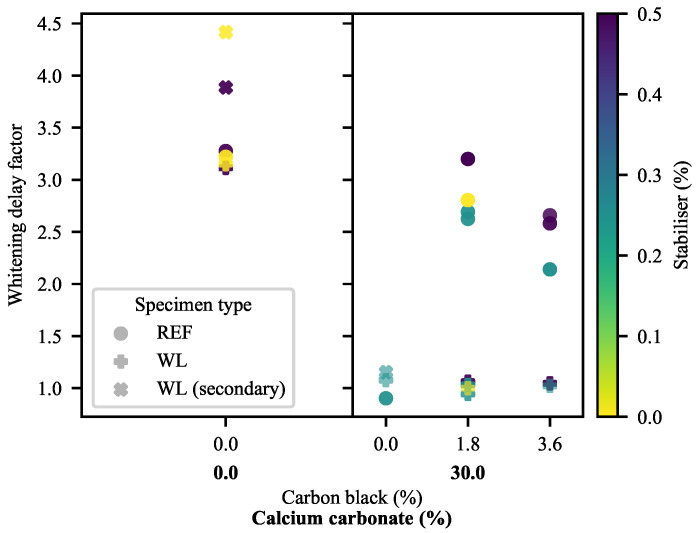
Delay factor of whitening from yield as a function of specimen type and CC, CB and SP loadings.

To better understand the deformation of the specimens, particularly the 30-CC specimens, it is useful to investigate the time-stamped still-images taken during the DIC deformation. In Figure 19, notable steps are laid out based on a WL specimen of 1.8-CB/30-CC/0.25-SP. These progress from the unextended specimen (0 s), to the onset of whitening (34 s), to the presence of substantial whitening (35 s), to failure (36 s). Of great use to the current discussion is the comparison of the times to the onset of whitening in the samples, shown comparatively in Figure 20. The whitening delay factor was calculated as the time to the onset of whitening divided by the time to yield. It is important to note that whitening is absent from a great many of the samples. In materials containing CB, initial whitening may be suppressed as a result of the darkness of the material not offering sufficient contrast and/or the suppression of some of the causes of whitening through the great ductility of the materials (particularly 0-CC), while the failures of the 60-CC materials are too abrupt for any whitening to be seen. In the CB-free materials, the white/off-white colour of the specimens adds a confounding factor, as the materials appear lighter as the paint droplets are moved apart. However, a clear but gradual change in gray level may be noted, albeit slight. This is frequently followed by a second and more localised/distinct whitening perpendicular to the direction of extension at the weld line in WL specimens.

In the 0-CC specimens, the similarity in the onset of the whitening of the REF specimens and the first stage of the whitening of the WL specimens is clear, with a distinct step to the second whitening stage of the WL specimens.

There exists a clear difference in the time to the onset of whitening between the WL and REF 30-CC specimens with CB, with little dependence on formulation in the WL specimens. However, the REF specimens appear to exhibit a negative correlation between the onset of whitening and the CB loading, contrasted with a positive correlation to SP loading. Conversely, in the 0-CB/30-CC material, the whitening stage of the REF specimen lines up nearly perfectly with the first whitening stage of the WL specimen.

It is anticipated that much of the whitening is driven by cavitation [38,40,80], with this effectively acting to initiate the second step after yielding in the typical deformation of polyolefins: fibrillation.

Debonding is also believed to be the cause of the second whitening stage of the 0-CB/30-CC WL specimen and the latter part of the whitening of its REF equivalent. Crazing on the weld lines of the 0-CB/0-CC WL specimens is believed to account for their second whitening stage. The first whitening shown by 0-CB specimens is believed to stem from the formation of surface roughness owing to a surface-relief stress-relaxation process. This is based on the comparison of the results presented here with the findings of Tanniru and Misra [37].

#### 3.2.2. Deformation at Last Viable Instance

**Figure 21:**
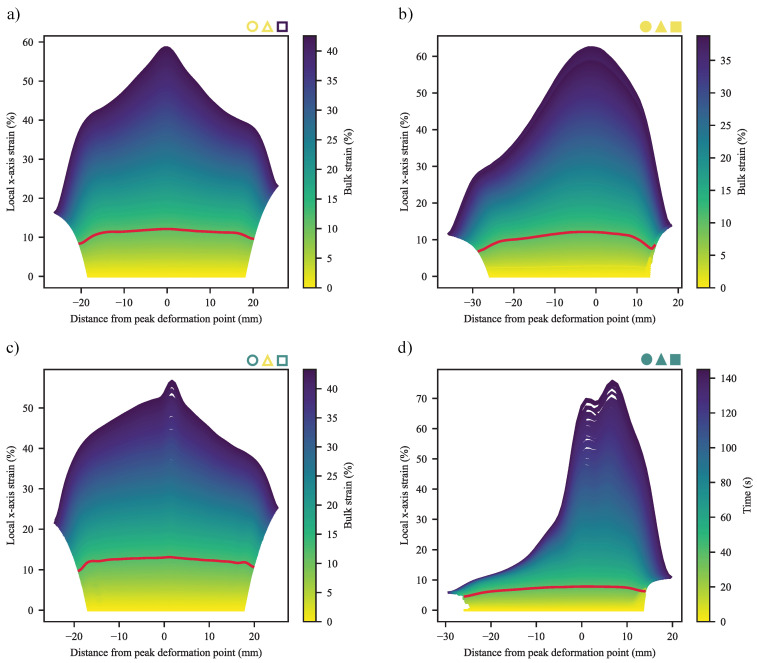
Time-series deformation profiles: last viable instance. (**a**) 0-CB/0-CC/0.5-SP, WL; (**b**) 0-CB/0-CC/0-SP, REF; (**c**) 1.8-CB/0-CC/0.25-SP, WL; (**d**) 1.8-CB/30-CC/0.25-SP, REF. The yield profiles are highlighted in red.

In general, the deformation at last viable instance mirrors that at yield, albeit with peaks amplified (Figure 21 and referring back to Figure 15). It is clear that deformation is initiated in—and remains centred on—the v-notch region in the WL specimens. At the same time, the broad, frequently singular peaks of the REF specimens may be seen. It must be kept in mind, here, that the 0-CC materials weren’t deformed to failure, due to the deformation regions falling outside the recording frame. In addition, the responses were centred on their peak deformation points at yield—to allow for ready comparison between Figure 15 and Figure 21.

In some cases, notably those of the REF specimens of 3.6-CB/30-CC/0.5-SP, 1.8-CB/30-CC/0.25-SP (shown in Figure 21d) and 1.8-CB/30-CC/0-SP, dual-peak behaviour is seen. Here, localised deformation sets in nearly simultaneously at two close-but-distinct locations, having first exhibited “broad bump” behaviour of the type shown in Figure 21b, with eventual failure one of the localised peaks. In these cases, consistently on the side of the moving clamp.

**Figure 22:**
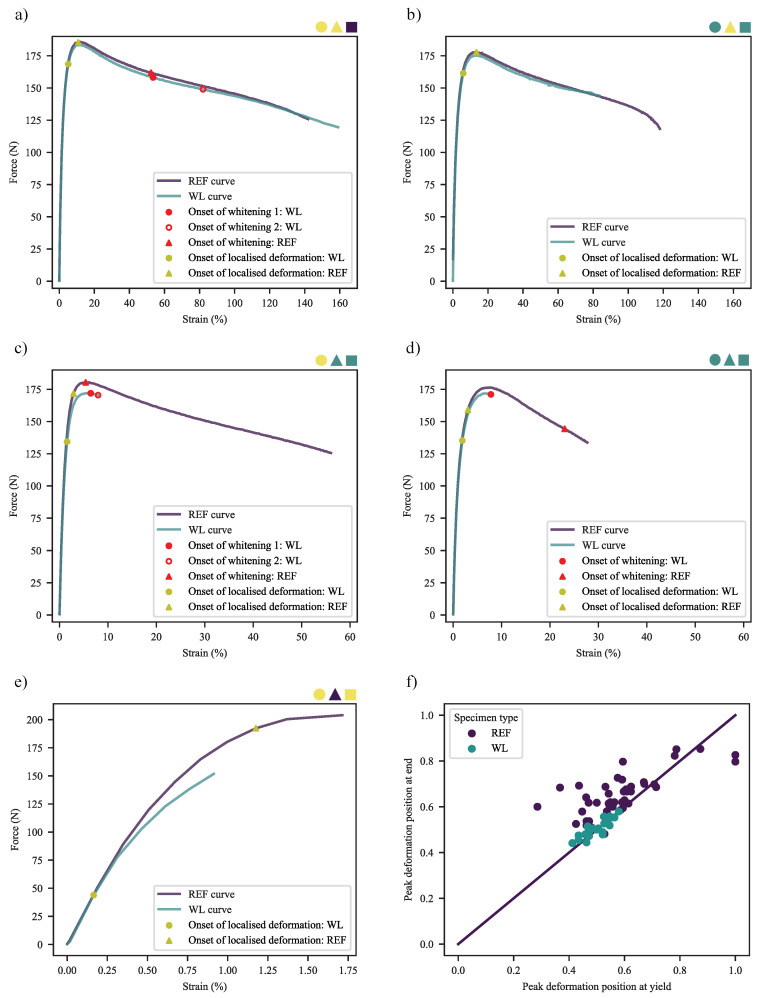
(**a**–**e**) Comparison of the peak-deformation-point force-strain curves for WL and REF specimens of (**a**) 0-CB/0-CC/0.5-SP, (**b**) 1.8-CB/0-CC/0.25-SP, (**c**) 0-CB/30-CC/0.25-SP, (**d**) 1.8-CB/30-CC/0.25-SP and (**e**) 0-CB/60-CC/0-SP, with markers for the onset of localised deformation and whitening. (**f**) Comparison of the maximum deformation positions for the yielding and last viable steps.

If, now, formulations are compared in terms of the force-strain responses (Figure 22a–e) of their peak deformation points (determined at the yield point), further findings may be made. First, it is clear that the behaviour discussed in the prior paragraphs is present, including that found for conventional tensile tests. This is notable, as the tests were performed at substantially different elongation rates. Next, the curves appear close to identical from zero strain to some point prior to yielding, with the WL specimens consistently deviating lower than their REF counterparts. Now, consider the markers placed on the figures, titled “Onset of localised deformation”, specifically those for the WL curves. The reader may be surprised to learn that these points were not placed conveniently where the curves deviate from one another, but they were determined from figures of the form of Figure 15 and Figure 16—with the points being indicative of the first time-step where a localised deformation peak could be seen. The accompanying points for REF curves were determined in the same way, based on the first appearance of the broader peaks that accompany specimens of this type. The time-steps used in the calculation of Figure 20 were used for the whitening points in these figures.

Referring first to the figures for 0-CC materials (Figure 22a,b), a key insight may be gleaned. The localised deformation of a REF specimen without CC commences at—or very close to—the yield point. In unfilled semi-crystalline polymers, specifically HDPE and other polyolefins [16,17,18,19,20,21,26], this is known to be the point where collective inter- and intralamellar slip sets in. Curiously, no new peak formation occurs at the yield points of the WL specimens of the same type. Instead, they exhibit their sole peak formation much earlier, as pointed out in the prior paragraph. Even in the case exemplified by the WL specimen of 1.8-CB/0-CC/0.25-SP in Figure 15, the bulk and localised peaks have their onsets at approximately the same time. Given that skin regions are typically completely bisected by the v-notches that accompany weld lines, it is believed that this is the result of these collective slips occurring in the core, which is know to be more ductile [81,82]. This, of course, is likely to be exasperated by the effect of v-notches acting as local stress concentrators [54].

Extending this analysis to the 30-CC materials (Figure 22c,d), the onset of localised deformation occurs somewhat prior to yielding in the REF specimens. This suggests that either collective slip has been supplanted as deformation mechanism, likely by debonding-induced cavitation/fibrillation [32]; that the more numerous nucleation sites, reduced continuity and increased force concentration [38] brought about by the particulate matter has resulted in collective slip being favoured as deformation mechanism, and, thereby, occurring at lower stress and strain than it normally would; or the combination of these. This is magnified, as in the prior paragraph, by the discontinuity in the skin region in the WL specimens. Based on the inelastic curvature seen prior to this localised deformation—indicating that nucleated inter- and intralamellar slip has already been taking place—and the much-later onset of stress-whitening (which is well-established as the result of cavitation [38,40,80]), it is believed to be more likely that continuous slip has been brought forward, with debonding-based cavitation only occurring slightly before stress-whitening makes its appearance.

While it is certain that the whitening of the REF specimen of 0-CB/30-CC/0.25-SP has an earlier onset than that of its CB equivalents, this only appears some time after the onset of localised deformation. The absence of the SEBS contained in CB is believed to result in this early cavitation [32,34,35]. It is likely that the debonding-based cavitation and surface relaxation processes commence some time before they can be noted, lent credence by the smooth progression visible in Figure 15 but it is considered unlikely that this is sufficient to supplant continuous slip.

0-CB/60-CC/0-SP (Figure 22e) was the only of the 60-CC materials to be suitable for analysis by this technique, owing to substantial noise and/or off-centre deformation encountered in the other cases. Nonetheless, it is interesting that it exhibits the same behaviour seen in the lower-CC formulations, albeit terminating in brittle failure with no noticeable whitening.

With reference to the abnormal $\epsilon {}_{B}$ trend seen in Figure 14 for the REF specimens of the 30-CC materials, the trend seen in Figure 20, Figure 22c,d may illuminate some of what is underlying. It must also be noted, at this point, that failure only occurs three seconds after the onset of whitening in Figure 22d, despite appearances. This is the unfortunate result of the strong whitening effect reducing contrast to the degree that the DIC system could no longer resolve the points in this region, making further data capture in this region impossible. Based on the available data, it appears as though the whitening points of the materials with CB are being left-shifted relative to yield in a fairly consistent manner with increasing CB. This means that, proportionally to the yield point, the onset of whitening becomes increasingly favourable with increasing CB in these specimens.

If the reader may tolerate some speculation, it is known that SEBS exists within HDPE as a soft and highly compatible dispersed phase [83] with debonding a distinct possibility, even in compatibilised form [57]. Based on this and the fact that CB is a particulate filler, both may act as a nucleation points for cavitation [54]. In the specific combination encountered here, it is possible that the stress concentration (and potential cavitation) brought about by the presence of the CC acts on the SEBS/CB domains, triggering one or both of them as nucleation points for the cavitation (and, thereby, whitening) that accompanies fibrillation. If the onset becomes more favourable with increasing CB, keeping in mind the just-discussed effects of CB, it stands to reason that the whitening process itself is favoured by increasing CB loading. Thereby, the now-more-concentrated stresses in the nodes and fibrils would act upon the CB and SEBS domains, encouraging further nucleation up to the end of the fibrillation step. At this stage, disentanglement may or not be favoured over normal levels. This, then, would result in the acceleration of the whitening processes in materials with higher CB loadings relative to that of materials with lower CB loadings—likely hastening the onset of chain disentanglement and, thereby, failure.

In the 0-CB specimen, the onset of whitening occurs very early in comparison with that of the CB specimens as a result of the ready debonding. However, the fibrils that result have not been “compromised” by the addition of CB discussed in the prior paragraph, and may be expected to better stand up to the drawing process as a result of the greatly reduced number of initiation sites for eventual disentanglement. This will increase their resistance to failure by disentanglement, despite the early onset of debonding, eventually extending their $\epsilon {}_{B}$. This topic requires further investigation, much more than can be accommodated in this work.

The localisation effects of weld lines can clearly be shown by tracking the DIC point of maximum deformation, correcting it for the number of points in the measurement and plotting for the yield and last viable steps (Figure 22f). Here, the 60-CC materials were excluded due to their brittle fracture not allowing consistent measure of the points at hand. The scale is calculated such that 0 is nearest the injection-moulding gate in the REF materials, while 0 and 1 are closest to the gates of the WL specimens. This may be viewed in greater context with reference to the injection-moulding forms presented in Supplementary Materials, Figure S1. The much tighter grouping of the WL results compared to that of the REF results is indicative of the localisation effects of weld lines, with the WL specimens typically failing on their weld lines. The REF samples clearly see their deformation points shifted away from the gate, consistent with prior findings for filled and unfilled materials [84,85,86]. In filled materials, it has been established that an uneven distribution of filler particles may occur along the length of a part, with a “bump” in concentration frequently in the latter half of tensile specimens [84,85]. Further, as a result of less time under flow conditions, the skin layer may become thinner further away from the gate. This results in somewhat compromised mechanical properties, with reductions in stiffness and yield strength [86]. It is interesting to note, further, that the deformation point at last viable measure is frequently shifted somewhat further away from the gate than the deformation point at yield in the REF specimens.

### 3.3. Scanning Electron Microscopy

The changes in failure mode mentioned in the prior paragraphs may be clearly seen through the analysis of the failure surfaces by scanning electron microscopy (SEM). The 0-CC materials could not be analysed using this technique, however, as a result of the extreme changes in topography brought about by their highly ductile deformation and eventual failure.

**Figure 23:**
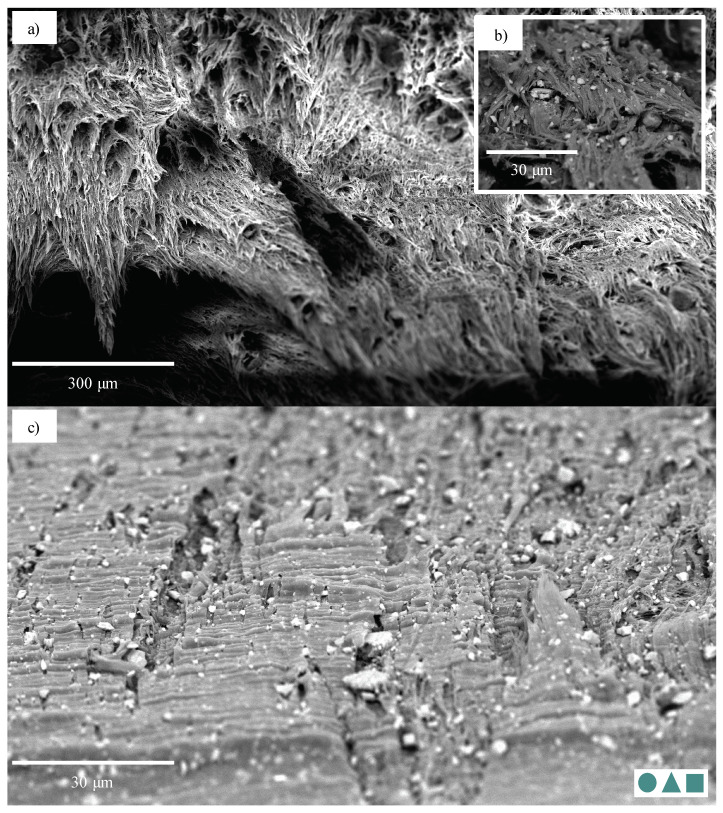
SEM micrographs of the failure regions of a 1.8-CB/30-CC/0.25-SP REF specimen: (**a**) general morphology showing extensive fibrillation; (**b**) detail of fibrillation and particle distribution; (**c**) specimen surface exhibiting wedge-tearing.

While the 30-CC REF specimens also exhibited ductile failure, this was to a lesser degree than that seen in the 0-CC specimens, enabling analysis. The 1.8-CB/30-CC/0.25-SP material is used as an example. Its ductile failure, characterised by extensive fibrillation, can clearly be seen in Figure 23a. Upon closer inspection (Figure 23b), it may be seen that the particles—over a range of sizes—are fairly evenly distributed and that clear debonding took place (with this likely resulting in fairly evenly distributed cavitation). This fibrillation does not appear to be layered, nor does that behaviour manifest in any of the other analysed specimens, likely due to the relatively low rate of extension [37]. The specimen surface near the failure region could be investigated (Figure 23c). Here, the extensive wedge-tearing behaviour that is characteristic of stress-whitening in HDPE reinforced with CC [37] may be seen, in many cases with nucleation on CC particles, and 30 μm agglomerates may be seen, albeit comparatively rarely.

**Figure 24:**
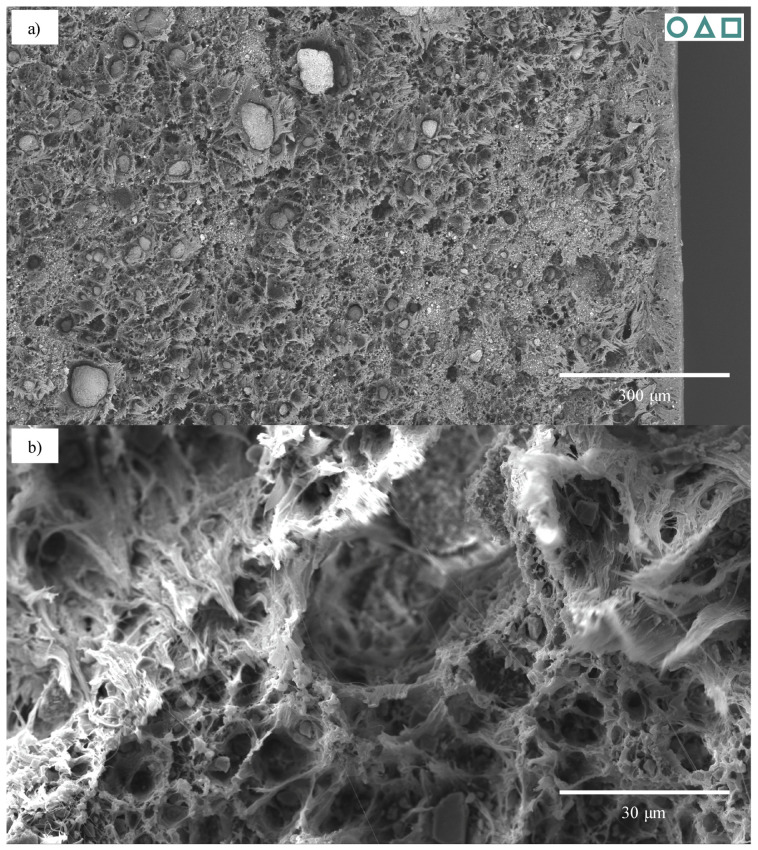
SEM micrographs of the failure regions of a 1.8-CB/30-CC/0.25-SP WL specimen: (**a**) general morphology showing a progression of mechanism moving away from the perimeter; (**b**) detail of fibrillation and particle effects, with much-reduced fibrillar extension.

In contrast, the failure of the 1.8-CB/30-CC/0.25-SP WL specimen exhibits far less fibrillation than that shown by its REF counterpart (Figure 24a,b). Substantial areas of fibrillation may be seen, particularly away from the surface of the specimen (pictured on the right-hand side of Figure 24a). These fibrils are notably less extended (Figure 24b) than those of the REF specimen, despite having otherwise similar dimensions, and have an seemingly greater proclivity for nucleation around larger particles (here primarily agglomerates). This may suggest an increased prevalence of these larger particles at the interface. Based on closer inspection of the agglomerates in Figure 24a, it appears as though at least a few of them fractured—based on their flat surfaces perpendicular to the direction of extension and the extensive ductile deformation that surrounds them. Interesting, here, is the smooth surface resulting from the v-notch in Figure 24a (more clearly visible in the secondary-electron-detector image of the same area in Supplementary Materials, Figure S26), flowing into “river hackles”. These hackles typically emanate from the failure origin [36], and suggest that failure did, indeed, originate from the v-notch. Furthermore, notable are the areas that exhibit even further reduced fibrillation, slightly to the right of centre in Figure 24a and Supplementary Materials, Figure S26, with these areas characterised by their distinctly flat profiles and numerous speckles of filler particles. This morphology suggests a more brittle failure than seen further away from the surface of the material.

While it is tempting, based on these flat sections, to conclude that the more brittle failure may result from a weak interface between the impinging melt fronts, value may be gained from looking at another example. Similar flat areas may be seen in the 0-CB/30-CC/0.25-SP WL specimen (Supplementary Materials, Figure S27a)—and may even be more prevalent—but attention must be paid to the areas with stepped topology near the perimeter of the specimen. This would suggest that, at least, some of the flat areas are not the remnants of ill-joined interfaces, but rather the result of unstable crack propagation owing to the merging of microvoids [37]. This is lent credence by the nanofibrils that may be seen at higher magnifications in these flat areas (Figure S27b). The increased prevalence of these flat areas likely stems from increased stiffness resulting the absence of the CB-accompanying SEBS. Elsewhere on the failure surface of this specimen, orifices left by filler pull-out with clear debonding may be seen (Supplementary Materials, Figure S27c).

**Figure 25:**
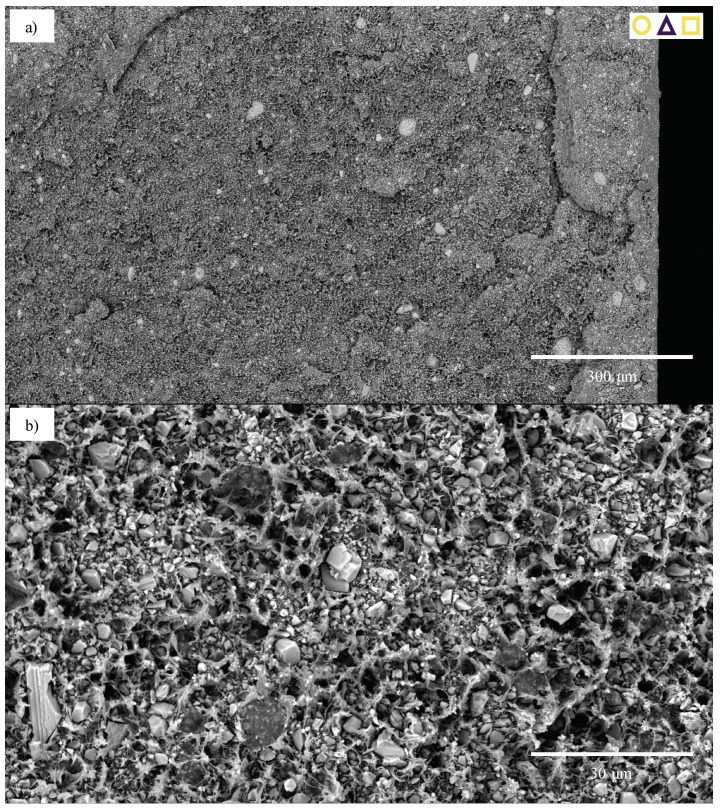
SEM micrographs of the failure regions of a 0-CB/60-CC/0-SP WL specimen: (**a**) general morphology showing dramatically reduced fibrillation and some changes in mechanism moving away from the perimeter; (**b**) detail of fibrillation and particle effects, with debonding and further-reduced fibrillar extension.

With the step up to a 60% CC loading, fibrillation is further suppressed, as can be seen in Figure 25 for a 0-CB/60-CC/0-SP WL specimen. First, from Figure 25b, it is clear that this material is very highly filled with CC, with substantial debonding having occurred during failure. From this stems the short fibrillar structures that surround the particles. A range of particle sizes can be seen, up to about 20 μm. Clear evidence may also be seen of the clean debonding and pull-out of the larger particles. Here, too, some large agglomerates may be seen, up to about 50 μm in diameter. Other than slightly more extensive fibrillation and fewer stepped changes in topography, the fracture surface of a 3.6-CB/60-CC/0.5-SP WL specimen is consistent with that discussed here. This morphology appears to be surprisingly consistent across the failure surfaces, excepting the smooth edges on the perimeters of the surfaces and the much-reduced flatter transition ranges between these and the fibrillated areas (Figure 24a). The smooth edges, here, are not thought to stem from the v-notches, but rather from the flattening of surface material that was extended during deformation and failure—as a result of their irregular shape. This, in large part, is influenced by the investigation of the REF specimens of the 60-CC materials, where these same unevenly shaped flat areas present themselves.

**Figure 26:**
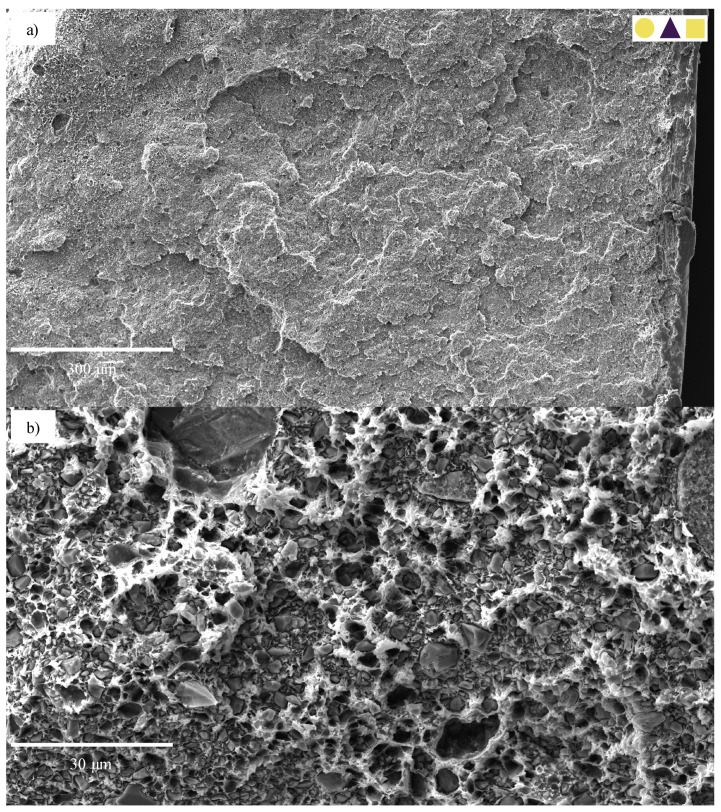
SEM micrographs of the failure regions of a 0-CB/60-CC/0-SP REF specimen: (**a**) general near-perimeter morphology showing shift towards unstable crack propagation; (**b**) detail of fibrillation and particle effects further away from the perimeter, with increased fibrillar extension compared to that in Figure 25.

The failure surfaces of the REF specimens of the 60-CC materials are also fairly consistent with that described in the prior paragraph, with the major deviation that the transitional areas (those with further suppressed fibrillation) between the perimeter and fibrillated areas are much larger (Figure 26a). Marginally increased fibrillation may be seen towards the centre of the specimens (Figure 26b), while the step changes in topography in the transitional areas are at their most prominent in the 0-CB/60-CC/0-SP specimen. These, again, are absent from the 3.6-CB/60-CC/0.5-SP specimen. The increase in the size of these flatter areas suggests that the more brittle failure mechanic played a larger role in these specimens than it did in the WL specimens, despite the greater $\epsilon {}_{B}$ of the REF specimens. This is the result of the material still exhibiting brittle failure, but not having the compromising weld-line section as an early initiation point for it. Thereby, more energy is available to allow for this more dramatic failure.

Based on the progression laid out above, it is clear that the failure of the specimens becomes less ductile in the presence of weld lines and with increasing CC loading. Further, the specimens—especially those without weld lines—that do not contain CB exhibit areas of unstable crack propagation. This has been tied to the the coalescence of numerous small (ductile) microvoids, with the extended areas shrinking back to a less-deformed state, resulting in the smoother surface appearance and the nanofibrils that are visible under high magnification [37]. Debonding-driven cavitation was shown to play a substantial role in the deformation and subsequent failure of the specimens, with the fracture of agglomerates thought to play a similar role. Particularly, these effects are believed to come to the fore approximately at the onset of whitening near the failure zone. In suitable materials, the formation of wedge tears is also believed to contribute to whitening—although this believed to be a more distributed phenomenon—occurring somewhat prior to the more localised whitening discussed prior.

## 4. Conclusions

The study of the tensile deformation of injection-moulded composites of HDPE with CB, CC and SP, with and without weld lines, has revealed the presence of many effects, some common between formulations and specimen types, and others not. Most notable are the findings, based on DIC, that localised deformation—thought to be based on continuous slip processes—may not only set in prior to the yielding of the material, but that point of onset and shape of the localised deformation are functions (sometimes complex) of the formulation and the presence/absence of weld lines. This was studied through the creation of novel factors quantifying the strength and localisation of these localised deformation peaks. Generally, the presence of weld lines resulted in early onset and much more localised deformation in these areas, while the REF specimens exhibited broad peaks with their onsets much closer to the yielding point than those of WL specimens. The presence of CB and/or CC was found to result in the sharpening of the WL peaks. Related to this is the finding that the pre-yield behaviours of WL and REF specimens are virtually indistinguishable, up to the point where localised deformation of the WL specimens sets in.

The next stage in the generally accepted mechanism of the deformation of HDPE—fibrillation—was investigated first through the onset of stress-whitening in these same specimens. Similar findings to those on continuous-slip processes were made here, with formulation and specimen-type dependencies found for the onset, type and number of stages of whitening seen. Distinct changes in the duration of the whitening regime prior to failure may also be seen, particularly in the 30% CC materials. In some cases, multiple stages of whitening may be seen: starting with a broad but slight whitening followed by a narrow and sharp whitening on a weld line. The two-stage whitening observed in some cases likely stems from first the onset of surface relaxation processes, followed by the cavitation of the bulk material.

Mechanistically, the cavitation-based effects leading into fibrillation could be better explained using the analysis of the failure surfaces of the samples containing CC using SEM. Here, it was found that increasing CC loading reduced the extent and prevalence of fibrillation, with failure becoming much more brittle. Further, it was clearly shown that the presence of weld lines further reduced the extension of the fibrils in these specimens, suggesting the sub-optimal interdiffusion of melt fronts in these cases—likely as a partial result of the fillers present in these materials. Some large agglomerates may be seen—and appear to have fractured during deformation—and are frequently surrounded by areas that suffered highly ductile deformation. In the absence of CB, crack propagation was found to be notably more unstable—resulting in a much more stepped topography than might otherwise be seen.

Statistical techniques were used, in addition to advanced visualisations, to investigate not only the above effects, but also impact of formulation and specimen type on the Young’s modulus, yield strength, elongation at yield and elongation at break of the specimens. Typically, CB and CC were found to have strong primary effects, with much of the effect of CB the result of its masterbatch resin—SEBS—rather than of the carbon black particles contained therein, in addition to notable interactive effects. Unsurprisingly, given their very wide range, the loadings of CC resulted in the most substantial changes—including a shift from ductile to brittle failure. As alluded to in the prior paragraph, however, the 30% loading of CC offered the most interesting results: consistent behaviour between the specimen types during the early stages of deformation, but a distinct change in the later stages owing to the combined effects of the continuous slip and cavitation deformation mechanisms. Divergent effects of weld lines were also seen, ranging from the ostensibly contradictory increases in Young’s modulus and elongation at yield seen in 0% CC materials to the sharp declines in yield strength, elongation at yield and elongation at break in the materials with CC. The effects of the additives and the weld lines are summarised in Table 2.

A deviation factor was calculated to study the correlation between the elastic and inelastic strain contributions seen in the materials prior to yielding, with strong correlation found. Unsurprisingly, stiffer materials exhibited much less proportional deviation than more ductile materials. Weld lines were found to affect this, with their presence resulting in a steeper decline in deviation factor with an increase in Young’s modulus than that seen in REF specimens. Weld lines were also found to result in a more consistent form of deformation, with much less change occurring in the points of maximum deformation of WL specimens than in those of REF specimens.

Further investigation is needed to determine the proportions of the change brought about by the compromised/discontinuous weld lines and the difference in “mixing effects” between the interdiffusion between the WL interfaces and the melt-mixing of a homogeneous material. In situ structural analysis is required for the deeper study of the shifts in behaviour reported here, with particular reference to the micromechanical deformation mechanisms thought to underlie these shifts. These mechanisms span those for pure polymers and for composites, and the comprehensive study of this topic will likely require the use of a number of in situ technologies.

## Figures and Tables

**Table 1:** Experimental design: formulations and feed points. Reproduced with permission from Viljoen and Labuschagné, Polymer Testing; published by Elsevier, 2020 [42].

	Component (%)					CC Feed	
**Formulation**	**HDPE**	**CC**	**CB**	**C944**	**I168**	**1**	**2**
01	65.90	30	3.60	0.30	0.20		•
02	65.90	30	3.60	0.30	0.20	•	
03	99.50	0	0	0.30	0.20		
04	100.00	0	0	0	0		
05	97.95	0	1.80	0.15	0.10		
06	95.90	0	3.60	0.30	0.20		
07	96.40	0	3.60	0	0		
08	69.75	30	0	0.15	0.10		•
09	67.70	30	1.80	0.30	0.20		•
10	67.95	30	1.80	0.15	0.10		•
11	67.95	30	1.80	0.15	0.10		•
12	68.20	30	1.80	0	0		•
13	66.15	30	3.60	0.15	0.10		•
14	39.50	60	0.00	0.30	0.20	•	•
15	40.00	60	0	0	0	•	•
16	37.95	60	1.80	0.15	0.10	•	•
17	35.90	60	3.60	0.30	0.20	•	•
18	36.40	60	3.60	0.00	0.00	•	•

**Table 2:** Summary of formulation and weld-line effects. ^1^ At a 60% CC loading, a much stronger decrease in WLF occurs than at the other loadings. ^2^ At a 0% CC loading, WLs have a positive effect, but this is offset by a strong negative effects at the other loadings. ^3^ In WL specimens, no notable interactive effect is seen, but a fairly strong synergism appears in REF specimens. ^4^ At a 0% CC loading, WLs have a slightly positive effect, but this is countered by negative effects at the other loadings. At these higher loadings, CB increases the difference between WL and REF specimens. ^5^ Minimal effects, but models suggest the presence of weak effects with substantial curvature due to the complexity of the data. ^6^ In the WL case, as the interactive effects from the REF case are believed to be erroneous. ^7^ Strong negative effect and strong CB dependency at 60% CC, moderate negative effect elsewhere.

	CB	CC	SP	CBxCC	CBxSP	CCxSP	WL
YM	↗	↑	•	↓	↗	•	↗
$\sigma {}_{Y}$	↘	↘	•	↓	↗	↘	${↘}^{1}$
$\epsilon {}_{Y}$	↗	↓	•	↓	•	↘	${↘}^{2}$
DF	•	↓	•	↑	•/ ${↗}^{3}$	↘	${•}^{4}$
$\epsilon {}_{B}$	•	↓	${•}^{5}$	${↑}^{6}$	↑	↗	↘/ ${↓}^{7}$

## Data Availability

The compact data presented in this study are available in the Supplementary Materials. Full data, for example stress-strain and DIC data, are available from the authors upon request.

## Supplementary Materials

Supplemental data and figures are available online at https://www.mdpi.com/2073-4360/13/4/527/s1, ranging from the fitted parameters of the models, their quality of fit and their plots, to various statistical investigations and parts-per-hundred-resin plots.
