# Supplementary material for: The Tensile Behaviour of Highly Filled High-Density Polyethylene Quaternary Composites: Weld-Line Effects, DIC Curiosities and Shifted Deformation Mechanisms
Source: Polymers (Basel). 2021 Feb 10;13(4):527. doi: 10.3390/polym13040527 (PMC7916787; doi:10.3390/polym13040527)
Supplement: Supplementary file 1 [file polymers-13-00527-s001.pdf]

polymers

MDPI

Article

The Tensile Behaviour of Highly Filled High-Density Polyethylene Quaternary Composites: Weld-Line Effects, DIC Curiosities and Shifted Deformation Mechanisms—Supplementary Materials

David Viljoen [1,*], Matthieu Fischer [2], Ines Kühnert [2] and Johan Labuschagné [1]

[1] Institute of Applied Materials, Department of Chemical Engineering, University of Pretoria, Hatfield, Pretoria, 0002, South Africa; johan.labuschagne@up.ac.za

[2] Polymer Processing Department, Leibniz-Institut für Polymerforschung Dresden e.V., Dresden, 01069, Germany; fischer-matthieu@ipfdd.de (M.F.); kuehnert@ipfdd.de (I.K.)

* Correspondence: wd.viljoen@tuks.co.za

Received: 30 November 2020; Accepted: 22 January 2021; Published: 10 February 2021

Abstract: The interactive effects between additives and weld lines, which are frequent injection-moulding defects, were studied in high-density polyethylene (HDPE) and compared to weld-line-free reference samples. These materials were formulated around a D- and I-optimal experimental design, based on a quadratic Scheffé polynomial model, with up to 60 wt% calcium carbonate, masterbatched carbon black and a stabiliser package. Where reasonable and appropriate, the behaviours of the systems were modelled using statistical techniques, for a better understanding of the underlying trends. The characterisations were performed through the use of conventional tensile testing, digital image correlation (DIC) and scanning electron microscopy (SEM). A range of complex interactive effects were found during conventional tensile testing, with DIC used to better understand and explain these effects. SEM is used to better understand the failure mechanics of some of these systems through fractography, particularly regarding particle effects. A measure is introduced to quantify the deviation of the pre-yield deformation curve from the ideal elastic case. Novel analysis of DIC results is proposed, through the use of combined time-series plots and measures quantifying the extent and localisation of peak deformation. Through this, it could be found that strong shifts in the deformation mechanisms occur as a function of formulation and the presence/absence of weld lines. Primarily, changes are noted in the onset of continuous inter- and intralamellar slip and cavitation/fibrillation, seen through the onset of localised deformation and stress-whitening.

Keywords: experimental design; high-density polyethylene; composite; digital image correlation; tensile testing; mixture model; deformation mechanics; calcium carbonate; carbon black; stabiliser; weld line

S1. Filler data

Table S1. Particle size data for OMYA Hydrocarb® 95 T - OG. Adapted from [1]

Property	Value	Unit
Residue on a 45 µm sieve (ISO 787/7)	0.02	%
d98	5	µm
d50	0.8	µm
Particles <2 µm	84	%
BET surface area (ISO 9277)	10	m^2/g

Table S2. Particle size data for Orion Engineered Carbons Printex® F 80. Adapted from [2]

Property	Value	Unit
Average particle size (ASTM D3849)	16	nm
BET surface area (ASTM S6556)	220	m^2/g

S2. Mould designs

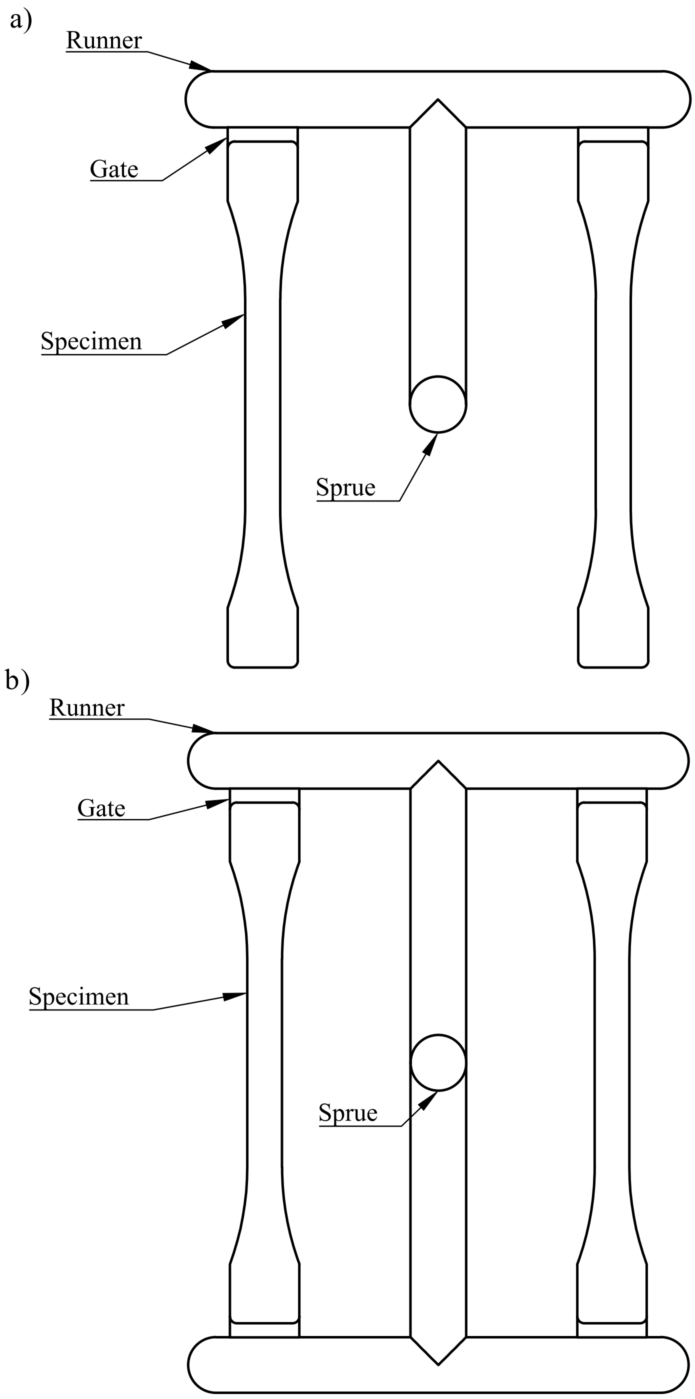

Figure S1. Drawings of the mould forms used in the production of the tensile specimens. Here, a) is the form used for the reference specimens and b) is the form used for the weld-line specimens.

S3. Experimental data

Table S3. Experimental data, conventional tensile testing

Form.	Weld line	Sample	YM (MPa)	σ_Y (MPa)	ϵ_Y (%)	ϵ_B (%)	Deviation contribution
1	No	1	1122.99	19.69	6.27	13.39	$5.08x10^{-2}$
1	No	2	1087.50	19.33	6.73	38.24	$5.50x10^{-2}$
1	No	3	1130.28	18.99	6.77	28.63	$5.54x10^{-2}$
2	No	1	1022.75	19.39	6.73	31.87	$5.40x10^{-2}$
2	No	2	1032.53	19.10	6.93	26.13	$5.67x10^{-2}$
2	No	3	1003.91	19.19	6.80	29.92	$5.46x10^{-2}$
3	No	1	902.44	22.30	9.52	833.47	$7.52x10^{-2}$
3	No	2	910.95	22.34	9.56	645.58	$7.80x10^{-2}$
3	No	3	896.13	22.04	9.69		$7.69x10^{-2}$
4	No	1	880.81	21.34	9.48	859.96	$7.64x10^{-2}$
4	No	2	834.84	21.44	9.75	704.82	$8.06x10^{-2}$
4	No	3	856.21	21.64	9.73		$8.07x10^{-2}$
5	No	1	827.58	21.78	10.65		$8.75x10^{-2}$
5	No	2	851.68	21.79	10.82	799.65	$9.05x10^{-2}$
5	No	3	824.70	21.00	10.97	733.86	$9.01x10^{-2}$
6	No	1	707.69	20.57	12.02	727.05	$9.80x10^{-2}$
6	No	2	710.80	20.15	12.14	679.88	$1.04x10^{-1}$
6	No	3	716.04	20.13	12.25	827.08	$1.02x10^{-1}$
7	No	1	767.17	20.36	12.19	648.89	$1.05x10^{-1}$
7	No	2	801.39	21.07	11.79	612.22	$9.95x10^{-2}$
7	No	3	753.37	20.87	11.61		$9.72x10^{-2}$
8	No	1	1276.00	21.05	4.49	24.82	$3.29x10^{-2}$
8	No	2	1287.56	21.04	4.50	87.96	$3.35x10^{-2}$
8	No	3	1236.28	20.38	4.31	85.23	$3.03x10^{-2}$
9	No	1	1135.56	19.51	5.40	20.04	$4.24x10^{-2}$
9	No	2	1081.63	19.21	5.90	46.64	$4.67x10^{-2}$
9	No	3	1021.81	19.51	5.93	34.47	$4.66x10^{-2}$
10	No	1	1035.27	19.34	6.02	35.33	$4.68x10^{-2}$
10	No	2	1096.57	19.36	5.92	43.96	$4.70x10^{-2}$
10	No	3	1144.02	19.05	5.80	48.55	$4.60x10^{-2}$
11	No	1	1103.07	19.57	5.67	42.00	$4.48x10^{-2}$
11	No	2	1115.10	19.20	5.89	69.66	$4.71x10^{-2}$
11	No	3	1098.25	19.32	5.89	37.25	$4.72x10^{-2}$
12	No	1	1047.76	19.25	6.04	90.57	$4.48x10^{-2}$
12	No	2	1092.73	19.41	6.08	115.05	$4.88x10^{-2}$
12	No	3	1086.90	19.39	5.87	65.90	$4.71x10^{-2}$
13	No	1	1028.56	19.29	6.48	23.57	$5.23x10^{-2}$
13	No	2	1015.27	19.37	6.36	12.51	$5.03x10^{-2}$
13	No	3	964.37	18.94	6.63	23.66	$5.36x10^{-2}$
14	No	1	2416.09	19.19	0.79	0.79	$1.56x10^{-3}$
14	No	2	2413.80	18.73	0.78	0.78	$1.87x10^{-3}$
14	No	3	2355.38	19.47	0.84	0.84	$2.13x10^{-3}$
15	No	1	2418.39	22.32	1.12	1.17	$4.24x10^{-3}$
15	No	2	2345.46	21.86	1.19	1.19	$5.02x10^{-3}$
15	No	3	2359.30	22.43	1.25	1.25	$5.35x10^{-3}$
16	No	1	2046.33	17.27	0.99	0.99	$3.93x10^{-3}$

16	No	2	1910.71	17.25	1.03	1.03	$3.71 x 10^{-3}$
16	No	3	1990.91	17.42	1.01	1.01	$3.86 x 10^{-3}$
17	No	1	1695.61	16.01	1.25	1.25	$5.98 x 10^{-3}$
17	No	2	1706.89	16.19	1.32	1.32	$6.48 x 10^{-3}$
17	No	3	1656.70	15.91	1.35	1.35	$7.02 x 10^{-3}$
18	No	1	1606.85	16.97	1.61	1.61	$9.03 x 10^{-3}$
18	No	2	1494.92	16.67	1.74	1.74	$1.01 x 10^{-2}$
18	No	3	1528.99	16.64	1.61	1.61	$8.92 x 10^{-3}$
1	Yes	1	1012.23	19.00	5.87	6.37	$4.61 x 10^{-2}$
1	Yes	2	1140.50	18.62	5.18	5.47	$4.04 x 10^{-2}$
1	Yes	3	862.05	18.89	6.72	7.18	
1	Yes	4	997.29	18.62	5.27	5.57	$4.02 x 10^{-2}$
2	Yes	1	996.60	18.88	5.71	6.34	$4.49 x 10^{-2}$
2	Yes	2	1096.47	18.69	5.24	5.54	$4.05 x 10^{-2}$
2	Yes	3	1097.13	18.90	5.27	5.57	$4.04 x 10^{-2}$
3	Yes	2	934.99	21.97	10.14	758.74	$8.53 x 10^{-2}$
3	Yes	3	881.15	20.89	10.16	773.10	$8.34 x 10^{-2}$
3	Yes	4	893.52	21.31	10.19	633.25	$8.47 x 10^{-2}$
4	Yes	1	973.76	22.73	9.35	730.48	$7.61 x 10^{-2}$
4	Yes	2	842.48	21.26	9.58	812.74	$7.83 x 10^{-2}$
4	Yes	3	873.02	21.55	9.74	685.63	$8.09 x 10^{-2}$
5	Yes	1	880.04	21.18	11.26	270.25	$9.66 x 10^{-2}$
5	Yes	2	784.57	20.86	11.39	692.86	$9.63 x 10^{-2}$
5	Yes	3	781.49	20.90	11.63	650.46	$9.75 x 10^{-2}$
6	Yes	1	741.29	20.58	12.40	642.06	$1.05 x 10^{-1}$
6	Yes	2	813.26	20.62	12.18	841.85	$1.05 x 10^{-1}$
6	Yes	3	737.95	20.12	12.59	647.60	$1.08 x 10^{-1}$
7	Yes	1	820.67	20.75	11.80	780.01	$1.01 x 10^{-1}$
7	Yes	2	758.31	20.24	11.70	420.91	$1.00 x 10^{-1}$
7	Yes	3	753.27	20.35	12.12	661.25	$1.03 x 10^{-1}$
8	Yes	1	1291.66	20.07	3.46	3.66	$2.39 x 10^{-2}$
8	Yes	2	1386.21	20.01	4.04	4.57	$3.01 x 10^{-2}$
8	Yes	3	1293.52	19.67	3.61	3.87	$2.52 x 10^{-2}$
9	Yes	1	1153.75	18.86	4.46	4.88	$3.34 x 10^{-2}$
9	Yes	2	1322.86	19.25	4.56	5.20	$3.43 x 10^{-2}$
9	Yes	3	1234.66	19.26	4.55	5.05	$3.33 x 10^{-2}$
10	Yes	1	1135.50	18.99	4.80	5.38	$3.57 x 10^{-2}$
10	Yes	2	1193.14	19.25	4.85	5.63	$3.68 x 10^{-2}$
10	Yes	3	1238.77	19.10	4.31	4.66	$3.18 x 10^{-2}$
11	Yes	1	1133.00	19.14	4.82	5.45	$3.70 x 10^{-2}$
11	Yes	2	1164.09	18.96	4.29	4.71	$3.18 x 10^{-2}$
11	Yes	3	1115.05	18.80	4.18	4.60	$2.79 x 10^{-2}$
12	Yes	1	1152.08	19.15	4.10	4.45	$2.96 x 10^{-2}$
12	Yes	2	1149.95	18.88	4.82	5.60	$3.52 x 10^{-2}$
12	Yes	3	1110.59	18.74	4.58	5.00	$3.36 x 10^{-2}$
13	Yes	1	1071.79	18.84	5.53	5.90	$4.10 x 10^{-2}$
13	Yes	2	1051.38	18.73	5.03	5.23	$3.68 x 10^{-2}$
13	Yes	3	1069.86	18.77	5.02	5.26	$3.79 x 10^{-2}$
14	Yes	1	2585.49	16.67	0.61	0.61	$8.66 x 10^{-4}$

14	Yes	2	2365.95	17.01	0.67	0.67	$1.28 x 10^{-3}$
14	Yes	3	2499.12	16.64	0.64	0.64	$1.14 x 10^{-3}$
15	Yes	1	2672.19	18.55	0.73	0.73	$1.78 x 10^{-3}$
15	Yes	2	2583.20	18.18	0.74	0.74	$1.60 x 10^{-3}$
15	Yes	3	2444.79	18.14	0.75	0.75	$1.79 x 10^{-3}$
16	Yes	1	2003.39	15.18	0.84	0.84	$2.65 x 10^{-3}$
16	Yes	2	1992.11	15.67	0.86	0.86	$2.79 x 10^{-3}$
16	Yes	3	2085.27	15.45	0.81	0.81	$2.46 x 10^{-3}$
17	Yes	1	1740.17	13.63	0.88	0.88	$2.86 x 10^{-3}$
17	Yes	2	1721.79	14.75	1.07	1.07	$4.37 x 10^{-3}$
17	Yes	3	1707.60	13.15	0.82	0.82	$2.71 x 10^{-3}$
18	Yes	1	1596.70	15.25	1.23	1.23	$5.22 x 10^{-3}$
18	Yes	2	1638.76	15.22	1.20	1.20	$5.40 x 10^{-3}$
18	Yes	3	1558.87	15.19	1.38	1.38	$7.17 x 10^{-3}$

S4. Model fitting

Table S4. Quality-of-fit parameters of chosen models.

Measure	Type	R^2	Adj. R^2	nMAE	SST-SSE-SSR	n(SST-SSE-SSR)	F-statistic	p-value	n(Bias)	Sig. parameters
Young's modulus (MPa)	REF	0.987	0.984	2.725×10^{-2}	3.512×10^{-3}	2.545×10^{-10}	3.642×10^{2}	1.110×10^{-16}	-6.525×10^{-9}	6
Young's modulus (MPa)	WL	0.983	0.980	2.886×10^{-2}	1.533×10^{-1}	9.702×10^{-9}	2.908×10^{2}	1.110×10^{-16}	1.291×10^{-8}	3
Yield strength (MPa)	REF	0.905	0.885	6.353×10^{-2}	1.648×10^{-5}	1.060×10^{-7}	4.641×10^{1}	1.110×10^{-16}	2.602×10^{-7}	4
Yield strength (MPa)	WL	0.967	0.960	3.164×10^{-2}	1.904×10^{-5}	7.733×10^{-8}	1.445×10^{2}	1.110×10^{-16}	-1.840×10^{-8}	6
Elongation at yield (%)	REF	0.997	0.997	1.282×10^{-2}	-1.986×10^{-6}	-2.740×10^{-9}	1.907×10^{3}	1.110×10^{-16}	2.482×10^{-8}	8
Elongation at yield (%)	WL	0.995	0.994	1.713×10^{-2}	-1.626×10^{-6}	-1.960×10^{-9}	9.387×10^{2}	1.110×10^{-16}	-1.864×10^{-8}	3
Elongation at break (%)	REF	0.989	0.986	2.158×10^{-2}	-7.065×10^{3}	-1.612×10^{-3}	3.877×10^{2}	1.110×10^{-16}	-2.078×10^{-2}	3
Elongation at break (%)	WL	0.957	0.948	3.302×10^{-2}	-3.018×10^{2}	-5.910×10^{-5}	1.104×10^{2}	1.110×10^{-16}	-5.356×10^{-4}	7
Deviation factor	REF	0.924	0.909	4.338×10^{-2}	-4.499×10^{-11}	-4.363×10^{-11}	5.985×10^{1}	1.110×10^{-16}	-1.026×10^{-8}	3
Deviation factor	WL	0.950	0.939	4.894×10^{-2}	-1.303×10^{-8}	-9.274×10^{-9}	9.207×10^{1}	1.110×10^{-16}	-4.918×10^{-9}	3
Corrected deviation factor	REF	0.636	0.561	6.820×10^{-2}	-4.007×10^{-8}	-7.886×10^{-8}	8.528×10^{0}	3.046×10^{-7}	3.541×10^{-8}	1
Corrected deviation factor	WL	0.698	0.636	7.303×10^{-2}	9.434×10^{-8}	1.598×10^{-7}	1.129×10^{1}	6.751×10^{-9}	-2.102×10^{-8}	1
DF(YM)	N/A	0.937	0.935	-	0	0	5.137×10^{2}	$<1.000 \times 10^{-4}$	-	4

Table S5. Fitted parameters and their significance levels

Measure	Type	Term	Exponential fit	Estimate	Standard error	Null hypothesis	T-statistic	p-value
Young's modulus (MPa)	REF	HDPE	Not exponential	$8.743x10^{2}$	$3.319x10^{1}$	$1.268x10^{3}$	$-1.185x10^{1}$	0.000
Young's modulus (MPa)	REF	CC	Not exponential	$4.454x10^{3}$	$1.088x10^{2}$	$1.268x10^{3}$	$2.928x10^{1}$	0.000
Young's modulus (MPa)	REF	CB	Not exponential	$1.474x10^{5}$	$6.599x10^{4}$	$1.268x10^{3}$	$2.214x10^{0}$	0.032
Young's modulus (MPa)	REF	SP	Not exponential	$-1.069x10^{6}$	$3.530x10^{6}$	$1.268x10^{3}$	$-3.032x10^{1}$	0.763
Young's modulus (MPa)	REF	HDPExCC	Not exponential	$-2.903x10^{3}$	$2.232x10^{2}$	0	$-1.300x10^{1}$	0.000
Young's modulus (MPa)	REF	HDPExCB	Not exponential	$-1.551x10^{5}$	$6.843x10^{4}$	0	$-2.267x10^{0}$	0.028
Young's modulus (MPa)	REF	HDPExSP	Not exponential	$1.070x10^{6}$	$3.547x10^{6}$	0	$3.017x10^{1}$	0.764
Young's modulus (MPa)	REF	CCxCB	Not exponential	$-1.873x10^{5}$	$6.842x10^{4}$	0	$-2.737x10^{0}$	0.009
Young's modulus (MPa)	REF	CCxSP	Not exponential	$1.097x10^{6}$	$3.547x10^{6}$	0	$3.093x10^{-1}$	0.759
Young's modulus (MPa)	REF	CBxSP	Not exponential	$1.303x10^{6}$	$3.556x10^{6}$	0	$3.665x10^{-1}$	0.716
Young's modulus (MPa)	WL	HDPE	Not exponential	$8.972x10^{2}$	$3.967x10^{1}$	$1.312x10^{3}$	$-1.045x10^{1}$	0.000
Young's modulus (MPa)	WL	CC	Not exponential	$4.688x10^{3}$	$1.292x10^{2}$	$1.312x10^{3}$	$2.613x10^{1}$	0.000
Young's modulus (MPa)	WL	CB	Not exponential	$7.654x10^{4}$	$7.870x10^{4}$	$1.312x10^{3}$	$9.558x10^{-1}$	0.344
Young's modulus (MPa)	WL	SP	Not exponential	$3.157x10^{6}$	$4.210x10^{6}$	$1.312x10^{3}$	$7.497x10^{-1}$	0.457
Young's modulus (MPa)	WL	HDPExCC	Not exponential	$-2.795x10^{3}$	$2.643x10^{2}$	0	$-1.058x10^{1}$	0.000
Young's modulus (MPa)	WL	HDPExCB	Not exponential	$-8.218x10^{4}$	$8.161x10^{4}$	0	$-1.007x10^{0}$	0.319
Young's modulus (MPa)	WL	HDPExSP	Not exponential	$-3.179x10^{6}$	$4.231x10^{6}$	0	$-7.514x10^{-1}$	0.456
Young's modulus (MPa)	WL	CCxCB	Not exponential	$-1.190x10^{5}$	$8.160x10^{4}$	0	$-1.459x10^{0}$	0.152
Young's modulus (MPa)	WL	CCxSP	Not exponential	$-3.174x10^{6}$	$4.231x10^{6}$	0	$-7.502x10^{-1}$	0.457
Young's modulus (MPa)	WL	CBxSP	Not exponential	$-2.726x10^{6}$	$4.242x10^{6}$	0	$-6.426x10^{-1}$	0.524
Yield strength (MPa)	REF	HDPE	Not exponential	$2.187x10^{1}$	$2.988x10^{-1}$	$1.965x10^{1}$	$7.450x10^{0}$	0.000
Yield strength (MPa)	REF	CC	Not exponential	$1.992x10^{1}$	$9.797x10^{-1}$	$1.965x10^{1}$	$2.782x10^{-1}$	0.782
Yield strength (MPa)	REF	CB	Not exponential	$2.031x10^{3}$	$5.941x10^{2}$	$1.965x10^{1}$	$3.386x10^{0}$	0.002
Yield strength (MPa)	REF	SP	Not exponential	$9.282x10^{3}$	$3.178x10^{4}$	$1.965x10^{1}$	$2.915x10^{-1}$	0.772
Yield strength (MPa)	REF	HDPExCC	Not exponential	$2.900x10^{0}$	$2.009x10^{0}$	0	$1.443x10^{0}$	0.156
Yield strength (MPa)	REF	HDPExCB	Not exponential	$-2.125x10^{3}$	$6.160x10^{2}$	0	$-3.449x10^{0}$	0.001
Yield strength (MPa)	REF	HDPExSP	Not exponential	$-9.306x10^{3}$	$3.193x10^{4}$	0	$-2.914x10^{-1}$	0.772
Yield strength (MPa)	REF	CCxCB	Not exponential	$-2.258x10^{3}$	$6.159x10^{2}$	0	$-3.666x10^{0}$	0.001
Yield strength (MPa)	REF	CCxSP	Not exponential	$-9.980x10^{3}$	$3.193x10^{4}$	0	$-3.125x10^{-1}$	0.756
Yield strength (MPa)	REF	CBxSP	Not exponential	$-6.340x10^{3}$	$3.201x10^{4}$	0	$-1.981x10^{-1}$	0.844

Yield strength (MPa)	WL	HDPE	Not exponential	2.166×10^{1}	2.203×10^{-1}	1.873×10^{1}	1.330×10^{1}	0.000
Yield strength (MPa)	WL	CC	Not exponential	1.123×10^{1}	7.179×10^{-1}	1.873×10^{1}	-1.044×10^{1}	0.000
Yield strength (MPa)	WL	CB	Not exponential	1.225×10^{3}	4.371×10^{2}	1.873×10^{1}	2.759×10^{0}	0.008
Yield strength (MPa)	WL	SP	Not exponential	1.475×10^{4}	2.338×10^{4}	1.873×10^{1}	6.299×10^{-1}	0.532
Yield strength (MPa)	WL	HDPExCC	Not exponential	1.112×10^{1}	1.468×10^{0}	0	7.573×10^{0}	0.000
Yield strength (MPa)	WL	HDPExCB	Not exponential	-1.281×10^{3}	4.533×10^{2}	0	-2.827×10^{0}	0.007
Yield strength (MPa)	WL	HDPExSP	Not exponential	-1.484×10^{4}	2.350×10^{4}	0	-6.315×10^{-1}	0.531
Yield strength (MPa)	WL	CCxCB	Not exponential	-1.355×10^{3}	4.532×10^{2}	0	-2.989×10^{0}	0.005
Yield strength (MPa)	WL	CCxSP	Not exponential	-1.523×10^{4}	2.350×10^{4}	0	-6.483×10^{-1}	0.520
Yield strength (MPa)	WL	CBxSP	Not exponential	-1.291×10^{4}	2.356×10^{4}	0	-5.479×10^{-1}	0.586
Elongation at yield (%)	REF	HDPE	Not exponential	9.571×10^{0}	1.057×10^{-1}	5.990×10^{0}	3.388×10^{1}	0.000
Elongation at yield (%)	REF	CC	Not exponential	-3.588×10^{0}	3.465×10^{-1}	5.990×10^{0}	-2.764×10^{1}	0.000
Elongation at yield (%)	REF	CB	Not exponential	-4.062×10^{2}	2.101×10^{2}	5.990×10^{0}	-1.962×10^{0}	0.056
Elongation at yield (%)	REF	SP	Not exponential	3.897×10^{4}	1.124×10^{4}	5.990×10^{0}	3.467×10^{0}	0.001
Elongation at yield (%)	REF	HDPExCC	Not exponential	-2.265×10^{0}	7.107×10^{-1}	0	-3.187×10^{0}	0.003
Elongation at yield (%)	REF	HDPExCB	Not exponential	5.005×10^{2}	2.179×10^{2}	0	2.297×10^{0}	0.026
Elongation at yield (%)	REF	HDPExSP	Not exponential	-3.916×10^{4}	1.129×10^{4}	0	-3.467×10^{0}	0.001
Elongation at yield (%)	REF	CCxCB	Not exponential	4.106×10^{2}	2.178×10^{2}	0	1.885×10^{0}	0.066
Elongation at yield (%)	REF	CCxSP	Not exponential	-3.932×10^{4}	1.129×10^{4}	0	-3.481×10^{0}	0.001
Elongation at yield (%)	REF	CBxSP	Not exponential	-3.715×10^{4}	1.132×10^{4}	0	-3.281×10^{0}	0.002
Elongation at yield (%)	WL	HDPE	Not exponential	9.582×10^{0}	1.609×10^{-1}	5.450×10^{0}	2.568×10^{1}	0.000
Elongation at yield (%)	WL	CC	Not exponential	1.093×10^{0}	5.243×10^{-1}	5.450×10^{0}	-8.309×10^{0}	0.000
Elongation at yield (%)	WL	CB	Not exponential	-5.046×10^{1}	3.193×10^{2}	5.450×10^{0}	-1.751×10^{-1}	0.862
Elongation at yield (%)	WL	SP	Not exponential	3.349×10^{3}	1.708×10^{4}	5.450×10^{0}	1.958×10^{-1}	0.846
Elongation at yield (%)	WL	HDPExCC	Not exponential	-1.574×10^{1}	1.072×10^{0}	0	-1.468×10^{1}	0.000
Elongation at yield (%)	WL	HDPExCB	Not exponential	1.304×10^{2}	3.311×10^{2}	0	3.940×10^{-1}	0.695
Elongation at yield (%)	WL	HDPExSP	Not exponential	-3.236×10^{3}	1.716×10^{4}	0	-1.885×10^{-1}	0.851
Elongation at yield (%)	WL	CCxCB	Not exponential	2.822×10^{1}	3.311×10^{2}	0	8.524×10^{-2}	0.932
Elongation at yield (%)	WL	CCxSP	Not exponential	-3.514×10^{3}	1.716×10^{4}	0	-2.047×10^{-1}	0.839
Elongation at yield (%)	WL	CBxSP	Not exponential	-2.814×10^{3}	1.721×10^{4}	0	-1.635×10^{-1}	0.871
Elongation at break (%)	REF	HDPE	Linear-space fit	2.896×10^{0}	2.845×10^{-1}	1.408×10^{0}	5.232×10^{0}	0.000
Elongation at break (%)	REF	CC	Linear-space fit	-3.857×10^{0}	8.009×10^{-1}	1.408×10^{0}	-6.574×10^{0}	0.000

Elongation at break (%)	REF	CB	Linear-space fit	$-2.701x10^{2}$	$4.889x10^{2}$	$1.408x10^{0}$	$-5.553x10^{-1}$	0.582
Elongation at break (%)	REF	SP	Linear-space fit	$1.040x10^{4}$	$2.615x10^{4}$	$1.408x10^{0}$	$3.976x10^{-1}$	0.693
Elongation at break (%)	REF	HDPExCC	Linear-space fit	$5.771x10^{0}$	$1.682x10^{0}$	0	$3.430x10^{0}$	0.001
Elongation at break (%)	REF	HDPExCB	Linear-space fit	$2.810x10^{2}$	$5.070x10^{2}$	0	$5.544x10^{-1}$	0.582
Elongation at break (%)	REF	HDPExSP	Linear-space fit	$-1.043x10^{4}$	$2.628x10^{4}$	0	$-3.970x10^{-1}$	0.694
Elongation at break (%)	REF	CCxCB	Linear-space fit	$2.545x10^{2}$	$5.067x10^{2}$	0	$5.023x10^{-1}$	0.618
Elongation at break (%)	REF	CCxSP	Linear-space fit	$-1.069x10^{4}$	$2.628x10^{4}$	0	$-4.068x10^{-1}$	0.686
Elongation at break (%)	REF	CBxSP	Linear-space fit	$-9.613x10^{3}$	$2.642x10^{4}$	0	$-3.639x10^{-1}$	0.718
Elongation at break (%)	WL	HDPE	Linear-space fit	$2.868x10^{0}$	$4.055x10^{-2}$	$1.073x10^{0}$	$4.427x10^{1}$	0.000
Elongation at break (%)	WL	CC	Linear-space fit	$8.033x10^{-1}$	$1.321x10^{1}$	$1.073x10^{0}$	$-2.042x10^{0}$	0.047
Elongation at break (%)	WL	CB	Linear-space fit	$1.366x10^{2}$	$8.045x10^{1}$	$1.073x10^{0}$	$1.685x10^{0}$	0.099
Elongation at break (%)	WL	SP	Linear-space fit	$1.067x10^{4}$	$4.303x10^{3}$	$1.073x10^{0}$	$2.480x10^{0}$	0.017
Elongation at break (%)	WL	HDPExCC	Linear-space fit	$-7.091x10^{0}$	$2.701x10^{1}$	0	$-2.625x10^{1}$	0.000
Elongation at break (%)	WL	HDPExCB	Linear-space fit	$-1.411x10^{2}$	$8.342x10^{1}$	0	$-1.692x10^{0}$	0.098
Elongation at break (%)	WL	HDPExSP	Linear-space fit	$-1.073x10^{4}$	$4.324x10^{3}$	0	$-2.481x10^{0}$	0.017
Elongation at break (%)	WL	CCxCB	Linear-space fit	$-1.370x10^{2}$	$8.341x10^{1}$	0	$-1.643x10^{0}$	0.107
Elongation at break (%)	WL	CCxSP	Linear-space fit	$-1.077x10^{4}$	$4.324x10^{3}$	0	$-2.492x10^{0}$	0.016
Elongation at break (%)	WL	CBxSP	Linear-space fit	$-1.049x10^{4}$	$4.336x10^{3}$	0	$-2.419x10^{0}$	0.020
Deviation factor	REF	HDPE	Not exponential	$8.49x10^{-1}$	$2.17x10^{-2}$	$7.53x10^{-1}$	$4.474x10^{0}$	$5.363x10^{-5}$
Deviation factor	REF	CC	Not exponential	$-1.68x10^{-1}$	$7.10x10^{-2}$	$7.53x10^{-1}$	$-1.296x10^{1}$	$1.280x10^{-16}$
Deviation factor	REF	CB	Not exponential	$-2.50x10^{1}$	$4.31x10^{1}$	$7.53x10^{-1}$	$-5.972x10^{-1}$	$5.534x10^{-1}$
Deviation factor	REF	SP	Not exponential	$9.73x10^{2}$	$2.30x10^{3}$	$7.53x10^{-1}$	$4.220x10^{-1}$	$6.751x10^{-1}$
Deviation factor	REF	HDPExCC	Not exponential	$1.16x10^{0}$	$1.46x10^{-1}$	0	$7.958x10^{0}$	$4.638x10^{-10}$
Deviation factor	REF	HDPExCB	Not exponential	$2.64x10^{1}$	$4.47x10^{1}$	0	$5.915x10^{-1}$	$5.572x10^{-1}$
Deviation factor	REF	HDPExSP	Not exponential	$-9.86x10^{2}$	$2.32x10^{3}$	0	$-4.257x10^{-1}$	$6.724x10^{-1}$
Deviation factor	REF	CCxCB	Not exponential	$3.70x10^{1}$	$4.47x10^{1}$	0	$8.284x10^{-1}$	$4.119x10^{-1}$
Deviation factor	REF	CCxSP	Not exponential	$-1.01x10^{3}$	$2.32x10^{3}$	0	$-4.372x10^{-1}$	$6.641x10^{-1}$
Deviation factor	REF	CBxSP	Not exponential	$-6.27x10^{2}$	$2.32x10^{3}$	0	$-2.702x10^{-1}$	$7.882x10^{-1}$
Deviation factor	WL	HDPE	Not exponential	$8.46x10^{-1}$	$2.07x10^{-2}$	$7.23x10^{-1}$	$5.983x10^{0}$	$3.306x10^{-7}$
Deviation factor	WL	CC	Not exponential	$-2.89x10^{-1}$	$6.77x10^{-2}$	$7.23x10^{-1}$	$-1.494x10^{1}$	$4.696x10^{-19}$
Deviation factor	WL	CB	Not exponential	$-1.09x10^{1}$	$4.11x10^{1}$	$7.23x10^{-1}$	$-2.826x10^{-1}$	$7.788x10^{-1}$
Deviation factor	WL	SP	Not exponential	$-9.21x10^{1}$	$2.20x10^{3}$	$7.23x10^{-1}$	$-4.227x10^{-2}$	$9.665x10^{-1}$

Deviation factor	WL	HDPExCC	Not exponential	1.04x10^{0}	1.39x10^{-1}	0	7.473x10^{0}	2.041x10^{-9}
Deviation factor	WL	HDPExCB	Not exponential	1.27x10^{1}	4.26x10^{1}	0	2.975x10^{-1}	7.674x10^{-1}
Deviation factor	WL	HDPExSP	Not exponential	9.74x10^{1}	2.21x10^{3}	0	4.410x10^{-2}	9.650x10^{-1}
Deviation factor	WL	CCxCB	Not exponential	2.21x10^{1}	4.26x10^{1}	0	5.194x10^{-1}	6.060x10^{-1}
Deviation factor	WL	CCxSP	Not exponential	7.71x10^{1}	2.21x10^{3}	0	3.493x10^{-2}	9.723x10^{-1}
Deviation factor	WL	CBxSP	Not exponential	4.49x10^{1}	2.21x10^{3}	0	2.030x10^{-2}	9.839x10^{-1}
Corrected deviation factor	REF	HDPE	Not exponential	1.02x10^{0}	3.34x10^{-2}	9.94x10^{-1}	8.59x10^{-1}	3.95x10^{-1}
Corrected deviation factor	REF	CC	Not exponential	8.52x10^{-1}	1.10x10^{-1}	9.94x10^{-1}	-1.30x10^{0}	2.00x10^{-1}
Corrected deviation factor	REF	CB	Not exponential	4.91x10^{1}	6.64x10^{1}	9.94x10^{-1}	7.24x10^{-1}	4.73x10^{-1}
Corrected deviation factor	REF	SP	Not exponential	2.49x10^{3}	3.55x10^{3}	9.94x10^{-1}	7.02x10^{-1}	4.86x10^{-1}
Corrected deviation factor	REF	HDPExCC	Not exponential	9.65x10^{-1}	2.25x10^{-1}	0	4.29x10^{0}	9.51x10^{-5}
Corrected deviation factor	REF	HDPExCB	Not exponential	-5.34x10^{1}	6.89x10^{1}	0	-7.76x10^{-1}	4.42x10^{-1}
Corrected deviation factor	REF	HDPExSP	Not exponential	-2.53x10^{3}	3.57x10^{3}	0	-7.07x10^{-1}	4.83x10^{-1}
Corrected deviation factor	REF	CCxCB	Not exponential	-5.29x10^{1}	6.89x10^{1}	0	-7.68x10^{-1}	4.47x10^{-1}
Corrected deviation factor	REF	CCxSP	Not exponential	-2.60x10^{3}	3.57x10^{3}	0	-7.29x10^{-1}	4.70x10^{-1}
Corrected deviation factor	REF	CBxSP	Not exponential	-1.40x10^{3}	3.58x10^{3}	0	-3.90x10^{-1}	6.98x10^{-1}
Corrected deviation factor	WL	HDPE	Not exponential	1.01x10^{0}	3.28x10^{-2}	1.00x10^{0}	4.15x10^{-1}	6.80x10^{-1}
Corrected deviation factor	WL	CC	Not exponential	9.32x10^{-1}	1.08x10^{-1}	1.00x10^{0}	-6.33x10^{-1}	5.30x10^{-1}
Corrected deviation factor	WL	CB	Not exponential	7.00x10^{1}	6.52x10^{1}	1.00x10^{0}	1.06x10^{0}	2.96x10^{-1}
Corrected deviation factor	WL	SP	Not exponential	2.87x10^{3}	3.49x10^{3}	1.00x10^{0}	8.22x10^{-1}	4.16x10^{-1}
Corrected deviation factor	WL	HDPExCC	Not exponential	1.00x10^{0}	2.21x10^{-1}	0	4.55x10^{0}	4.02x10^{-5}
Corrected deviation factor	WL	HDPExCB	Not exponential	-7.36x10^{1}	6.76x10^{1}	0	-1.09x10^{0}	2.82x10^{-1}
Corrected deviation factor	WL	HDPExSP	Not exponential	-2.88x10^{3}	3.50x10^{3}	0	-8.22x10^{-1}	4.15x10^{-1}
Corrected deviation factor	WL	CCxCB	Not exponential	-8.26x10^{1}	6.76x10^{1}	0	-1.22x10^{0}	2.28x10^{-1}
Corrected deviation factor	WL	CCxSP	Not exponential	-2.96x10^{3}	3.50x10^{3}	0	-8.45x10^{-1}	4.03x10^{-1}
Corrected deviation factor	WL	CBxSP	Not exponential	-2.36x10^{3}	3.51x10^{3}	0	-6.73x10^{-1}	5.05x10^{-1}
DF(YM)	N/A	Intercept	Not exponential	1.16x10^{0}	1.30x10^{-2}	6.88x10^{-1}	8.881x10^{1}	<1.000x10^{-4}
DF(YM)	N/A	ST[WL}	Not exponential	-1.37x10^{-2}	4.87x10^{-3}	0	-2.810x10^{0}	6.000x10^{-3}
DF(YM)	N/A	YM	Not exponential	-3.60x10^{-4}	9.34x10^{-6}	0	-3.860x10^{1}	<1.000x10^{-4}
DF(YM)	N/A	ST[WL}x(YM-$\overline{YM}$)	Not exponential	-2.80x10^{-5}	9.34x10^{-6}	0	-2.990x10^{0}	3.500x10^{-3}

S5. Parts-per-hundred-resin plots

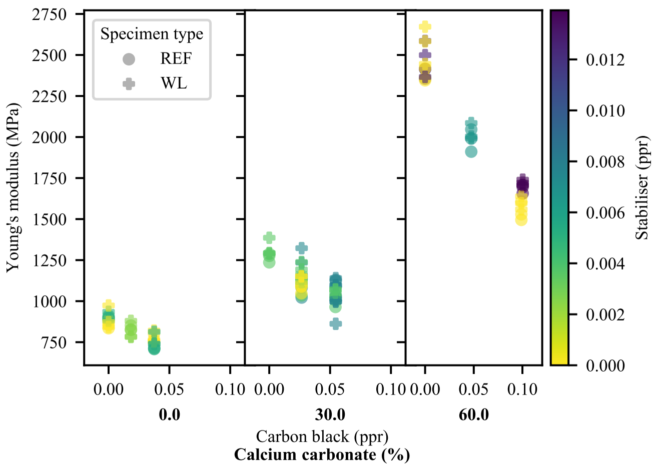

Figure S2. Plot of Young's modulus as a function of additive loadings, with the lesser loadings in ppr.

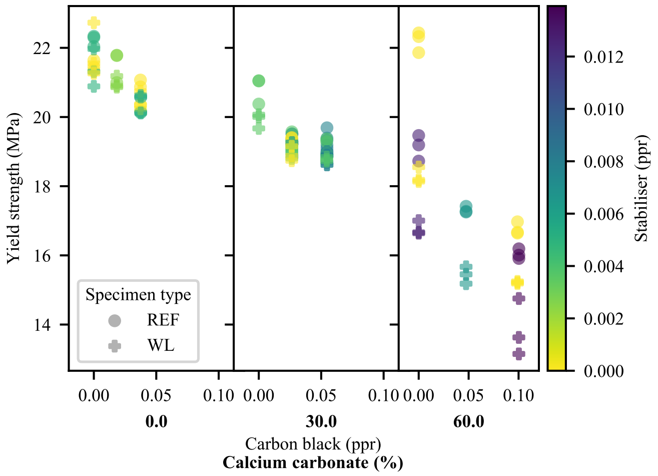

Figure S3. Plot of yield strength as a function of additive loadings, with the lesser loadings in ppr.

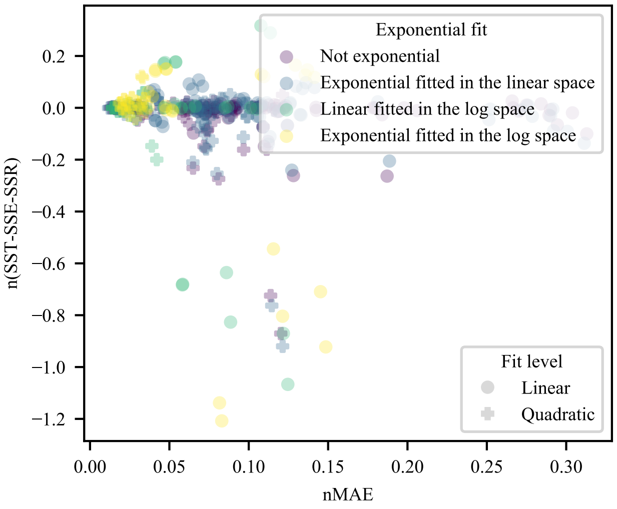

Figure S4. Check on the validity of the assumption of SST=SSE+SSR for the use of R^2 as a measure of quality of fit.

S6. Model deviations

A number of statistical phenomena and assumptions may be investigated, with particular interest towards the quality-of-fit achieved, the validity of certain measures and the presence of bias.

First, the validity of the assumption of SST=SSE+SSR that underpins the use of R^2 (and, thereby, adjusted R^2) can be verified, as shown in Figure S4. As the y-axis here has been normalised on SST, it is clear that the assumption fails in many cases. It is interesting to note, however, that the validity of the assumption improves dramatically as the quality of fit improves, particularly at nMAE values of less than 0.03. As the models with smaller error terms are those of interest, this is a useful finding. Also clear is that the SST term frequently underestimates the true total error, as may be seen from the far much broader range of negative values than that of positive values. It must be noted that only formulation-based models are included here, as the model correlating the DF to YM was fit using standard least-squares regression.

If, next, the correlation between adjusted R^2 and nMAE is investigated (Figure S5), it may be seen that the correlation between adjusted R^2 and nMAE improves dramatically below an nMAE of around 0.05, and certainly below the 0.03 found in the prior paragraph. This suggests that adjusted R^2 has some value in the assessment of these low-error models.

An investigation of the bias that is inherent to many of the model predictions (Figure S6) reveals cause for care in the selection of models, in that even some low-error models have consistent and substantial bias. What is also revealed is that a substantial portion of the models have very low normalised bias, through the distinct line visible in Figure S6 centred on a normalised bias of zero. Of these models, the vast majority are fitted in the linear space, lending credence to the concerns with the use of log-space models noted in the primary paper.

As a matter of curiosity and expectation, it may be seen in Figure S7 that the number of statistically significant parameters (according to the methodology discussed in the primary paper) generally increases with decreasing nMAE. The substantial ranges of nMAE that may accompany a given (lower) number of significant parameters must, however, be noted. Of further interest is the substantial left-shift of exponential and log-space models for a given number of significant parameters.

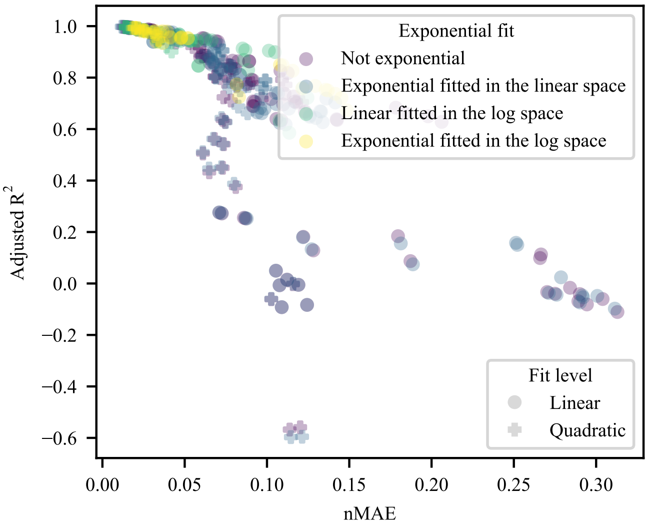

Figure S5. Check on the correlation between nMAE and adjusted R^2. Note the improvement in correlation seen at lower nMAE values.

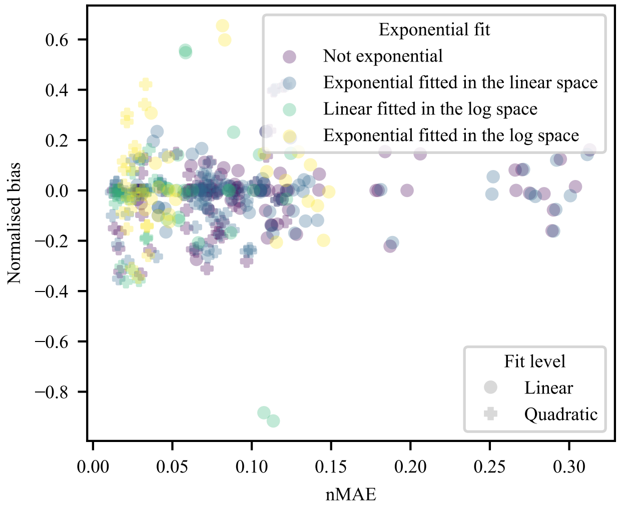

Figure S6. Distribution of normalised bias with nMAE. Note the distinct line centred on a normalised bias of zero.

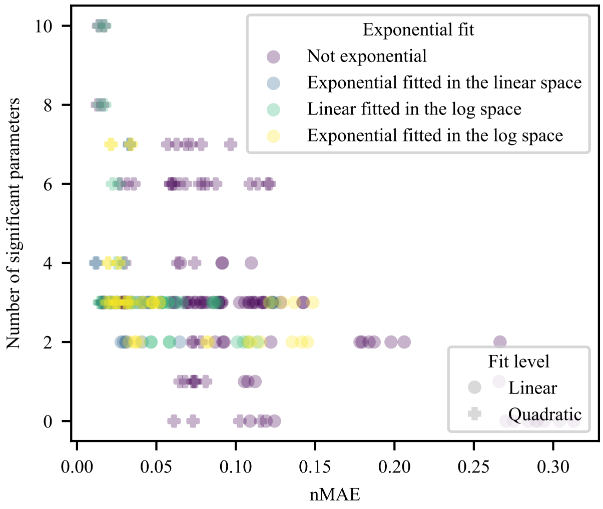

Figure S7. Correlation between nMAE and the number of significant parameters of a model.

S7. Model predictions

S7.1. Young's modulus

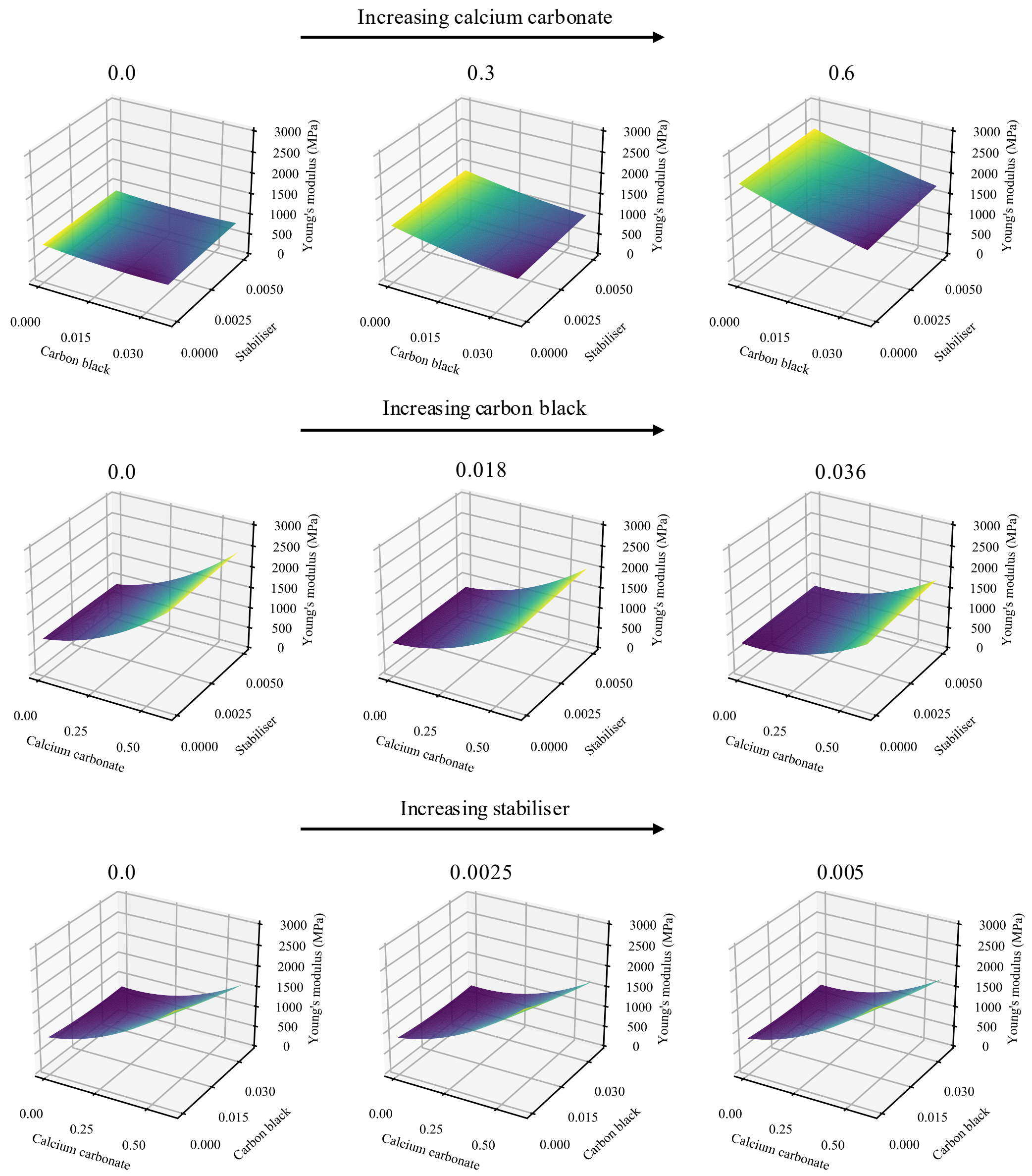

Figure S8. Weld-line-free Young's modulus model predictions. Note the similarity to Figure 3 in the primary paper, as well as the slight differences shown in Figure 2 of the primary paper.

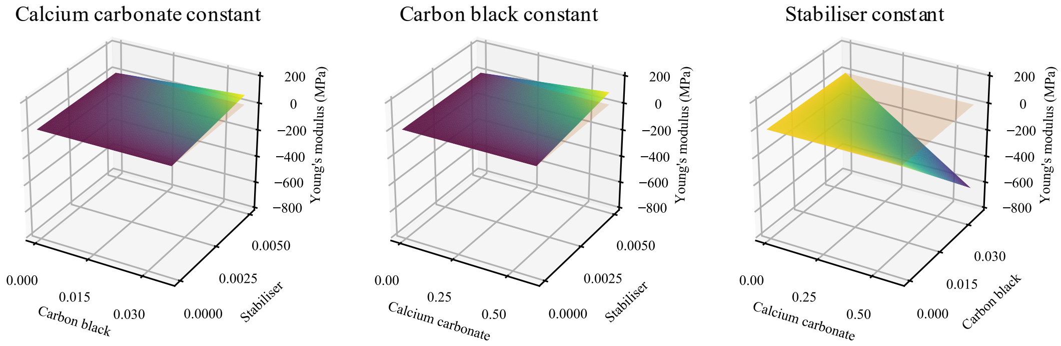

Figure S9. Weld-line-free Young's modulus model predictions for interactive effects. Note the similarity to Figure 3 in the primary paper, as well as the slight differences shown in Figure 2 of the primary paper. The change in interactive effects between CB and SP and CC and SP is also notable, as it shows the uncertainty that is encountered with weaker effects.

S7.2. Yield strength

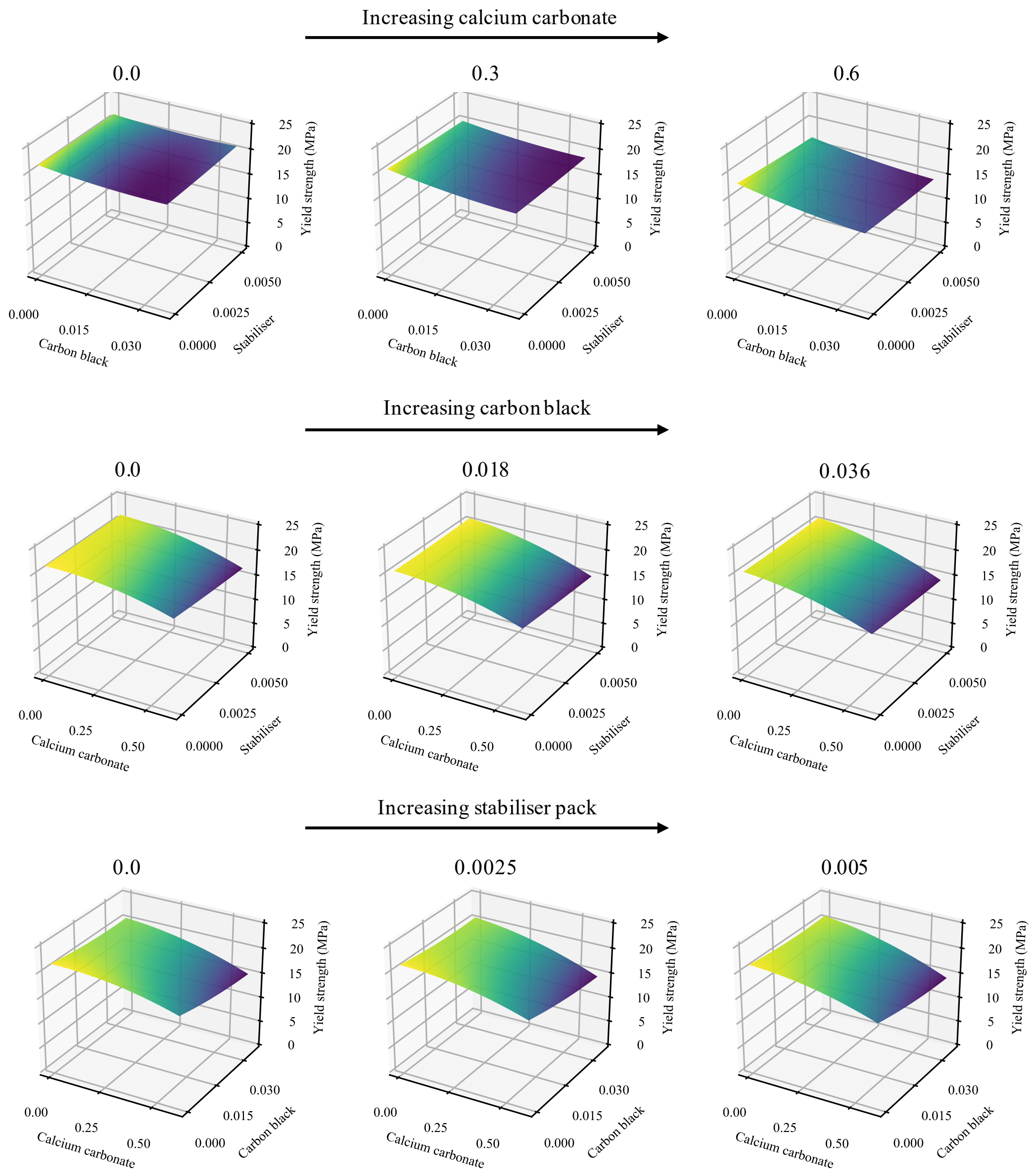

Figure S10. Modelled influence of the formulation variables on the yield strength of WL specimens. Note the increasing severity of the step-down in strength with increasing CC loading, as well as the sharper decline with increasing CBxCC.

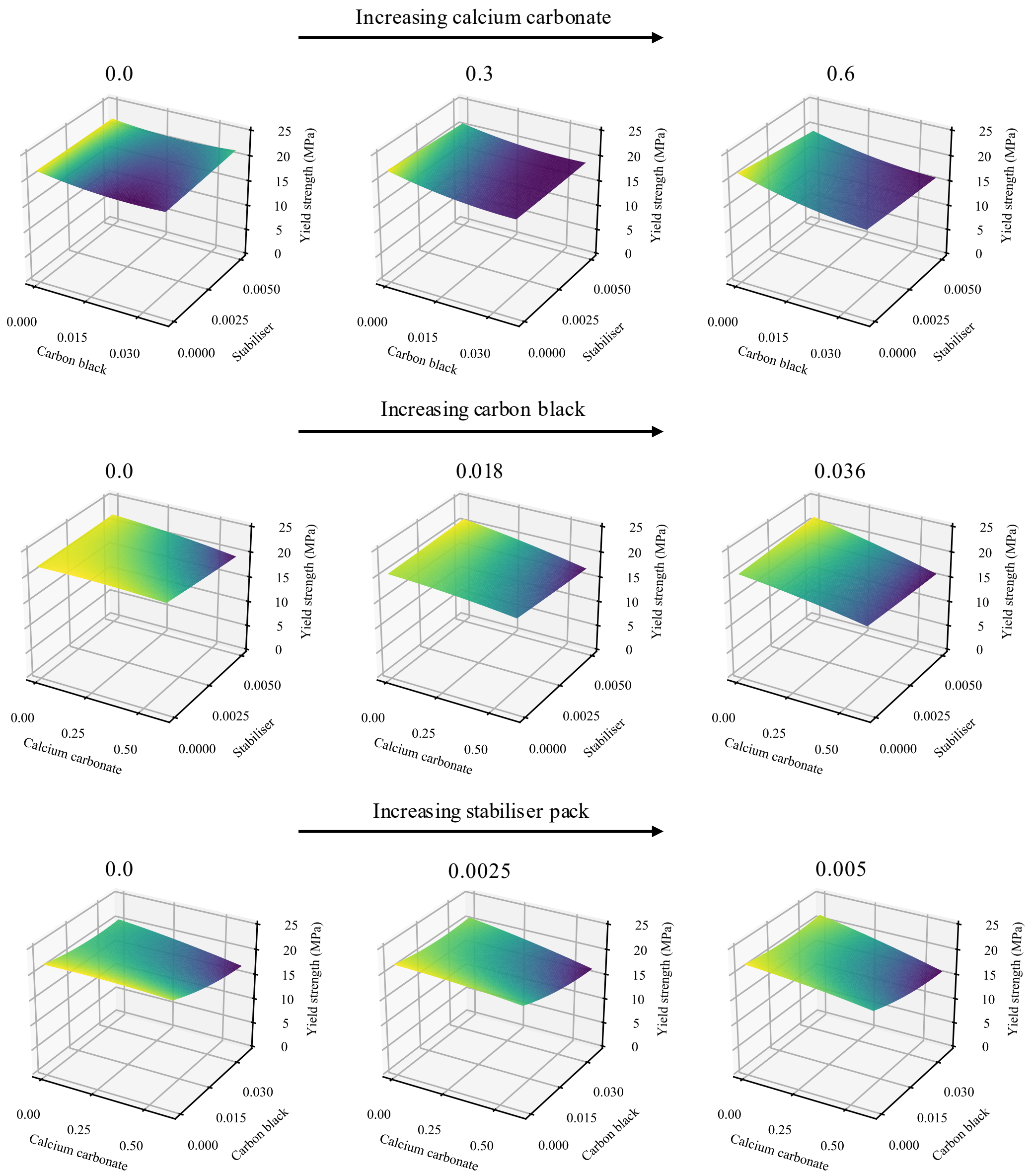

Figure S11. Modelled influence of the formulation variables on the yield strength of REF specimens. Note the increasing severity of the step-down in strength with increasing CC loading, as well as the sharper decline with increasing CBxCC. The similarity in trend to that seen in Figure S10 is also notable, and the reason for the necessity of direct comparison—as, otherwise, the behaviours may have been taken as identical.

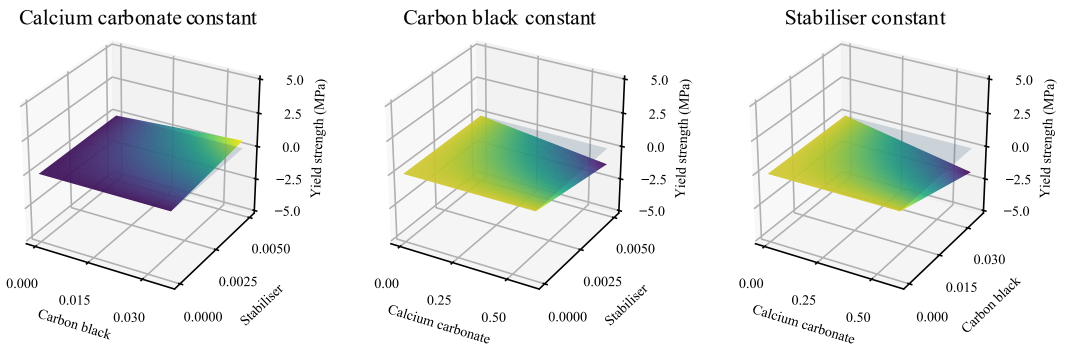

Figure S12. Modelled influence of the interactive effects of the formulation variables on the yield strength of WL specimens. Note the apparent weak synergy between CB and SP, as well as the much stronger antagonisms between CC/SP and CB/CC.

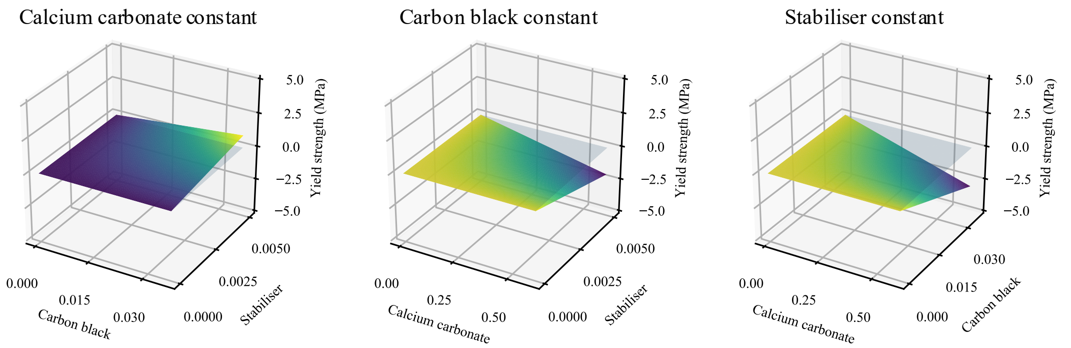

Figure S13. Modelled influence of the interactive effects of the formulation variables on the yield strength of REF specimens. Note the apparent weak synergy between CB and SP, as well as the much stronger antagonisms between CC/SP and CB/CC. There exists great similarity between the behaviour seen here and that in Figure S12.

S7.3. Elongation at yield

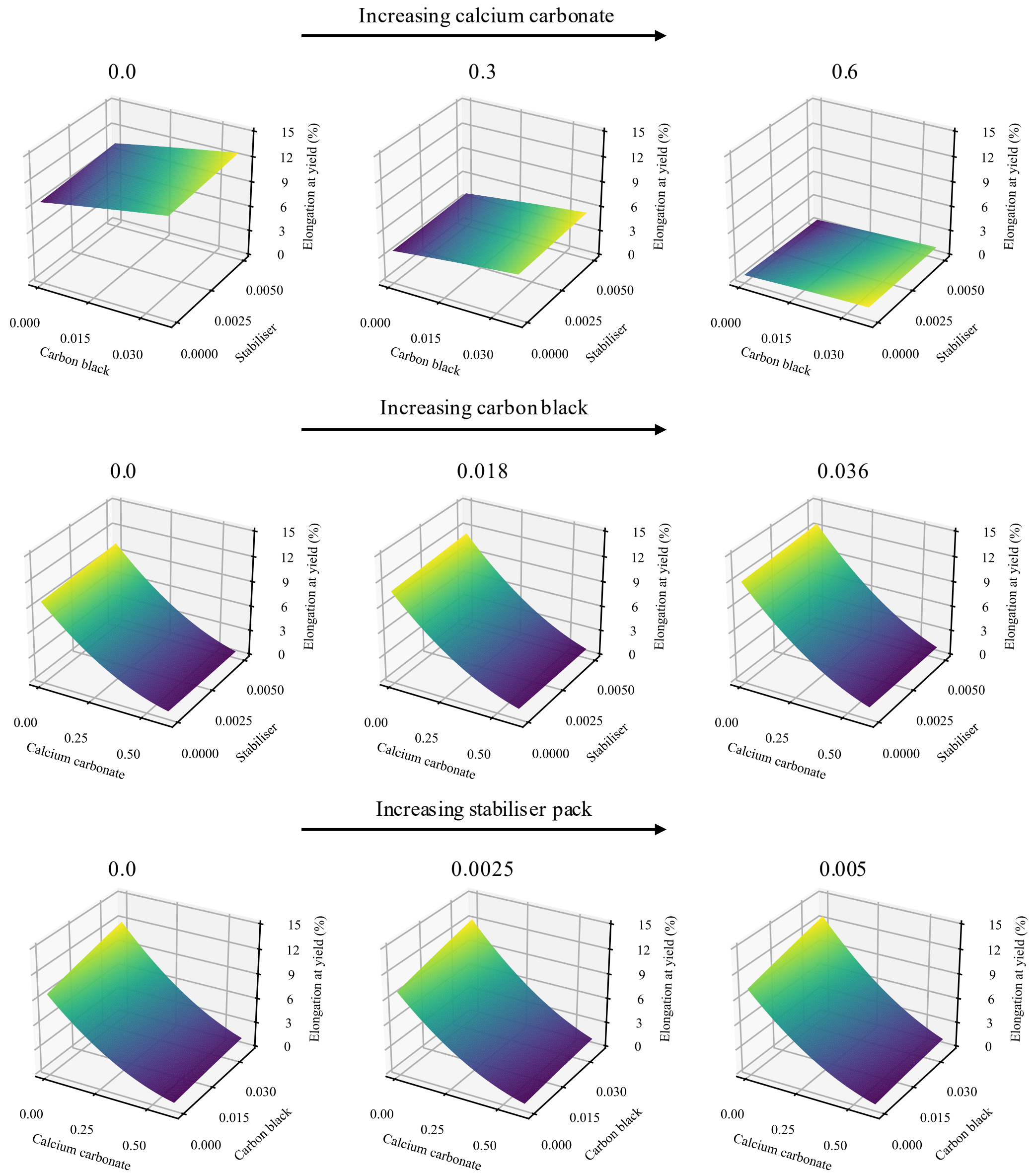

Figure S14. Modelled influence of the formulation variables on the elongation at yield of WL specimens. Note the extreme decrease as a result of increasing CC loading, counteracted by fairly substantial primary increases brought about by CB. SP has a minimal primary effect. Hints of the strong antagonism between CB and CC can be seen.

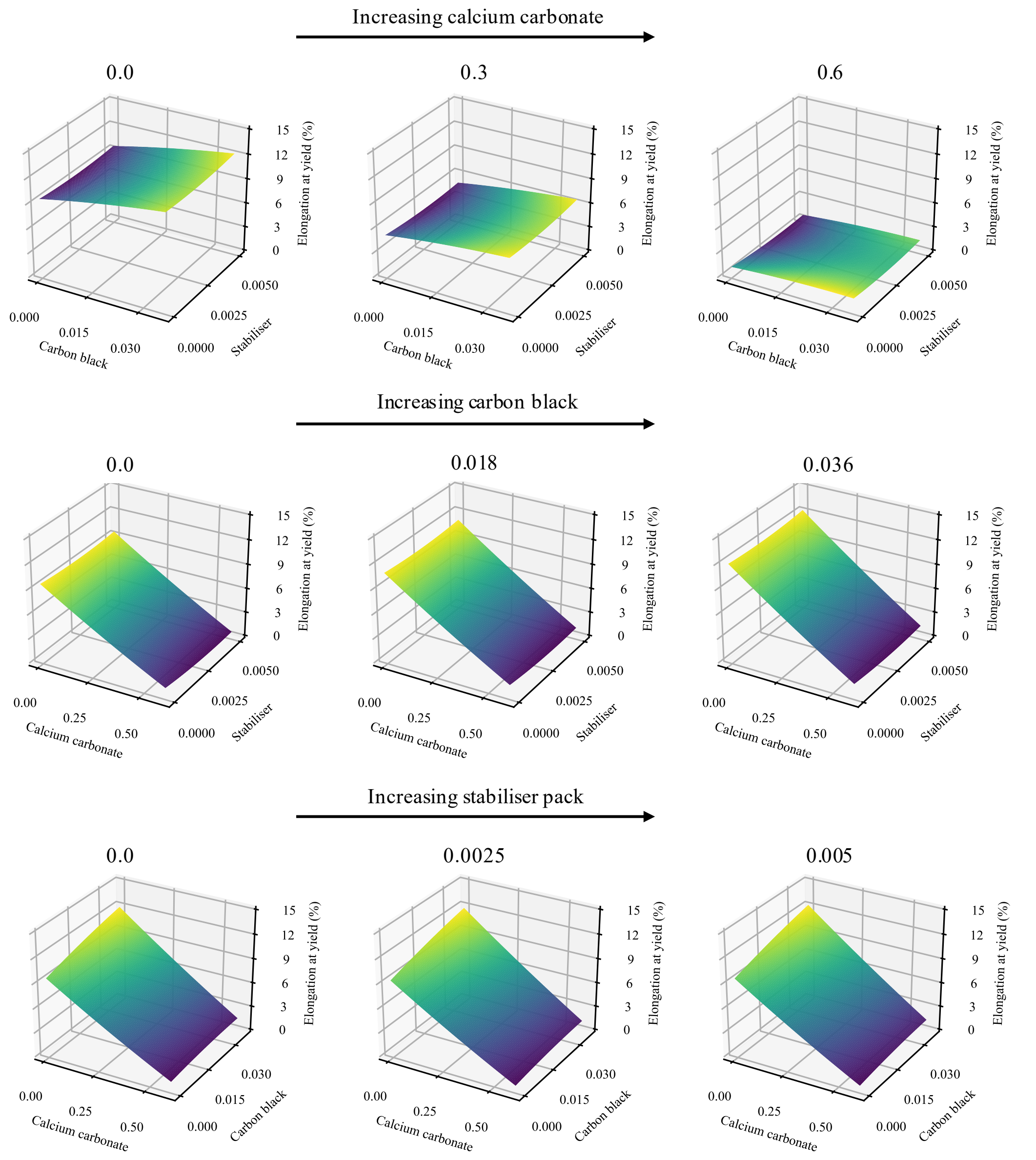

Figure S15. Modelled influence of the formulation variables on the elongation at yield of REF specimens. Note the similarity to Figure S14, albeit with increased elongation at yield at a 30 % CC loading. This results in a model that appears very linear, indicating insubstantial matrix/additive interactive effects.

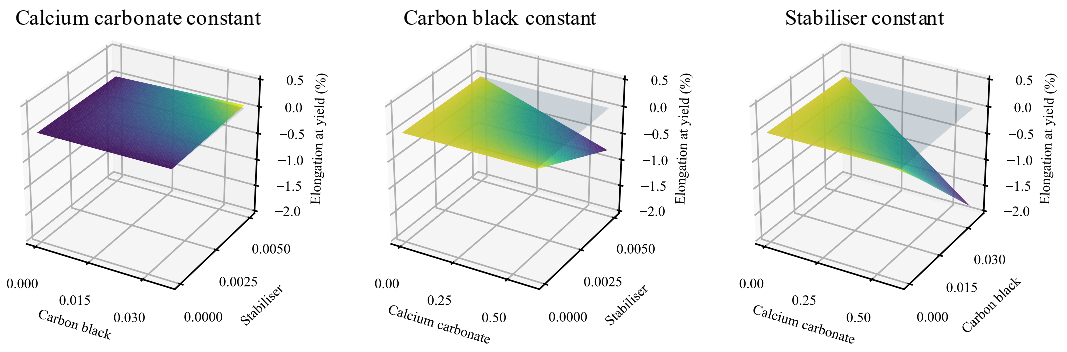

Figure S16. Modelled influence of the interactive effects of the formulation variables on the elongation at yield of WL specimens. Note the very strong antagonism between CB and CC, in addition to the moderate antagonism between CC and SP.

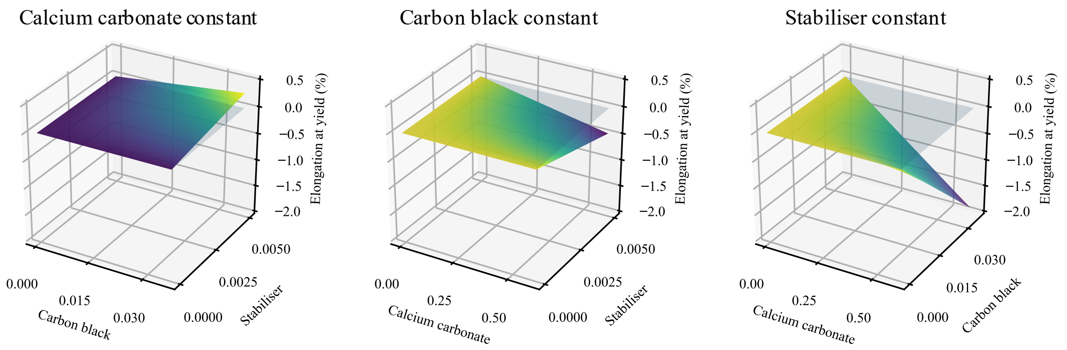

Figure S17. Modelled influence of the interactive effects of the formulation variables on the elongation at yield of REF specimens. Note the similarity to Figure S16, albeit with somewhat reduced antagonism between CC and SP.

S7.4. Deformation factor

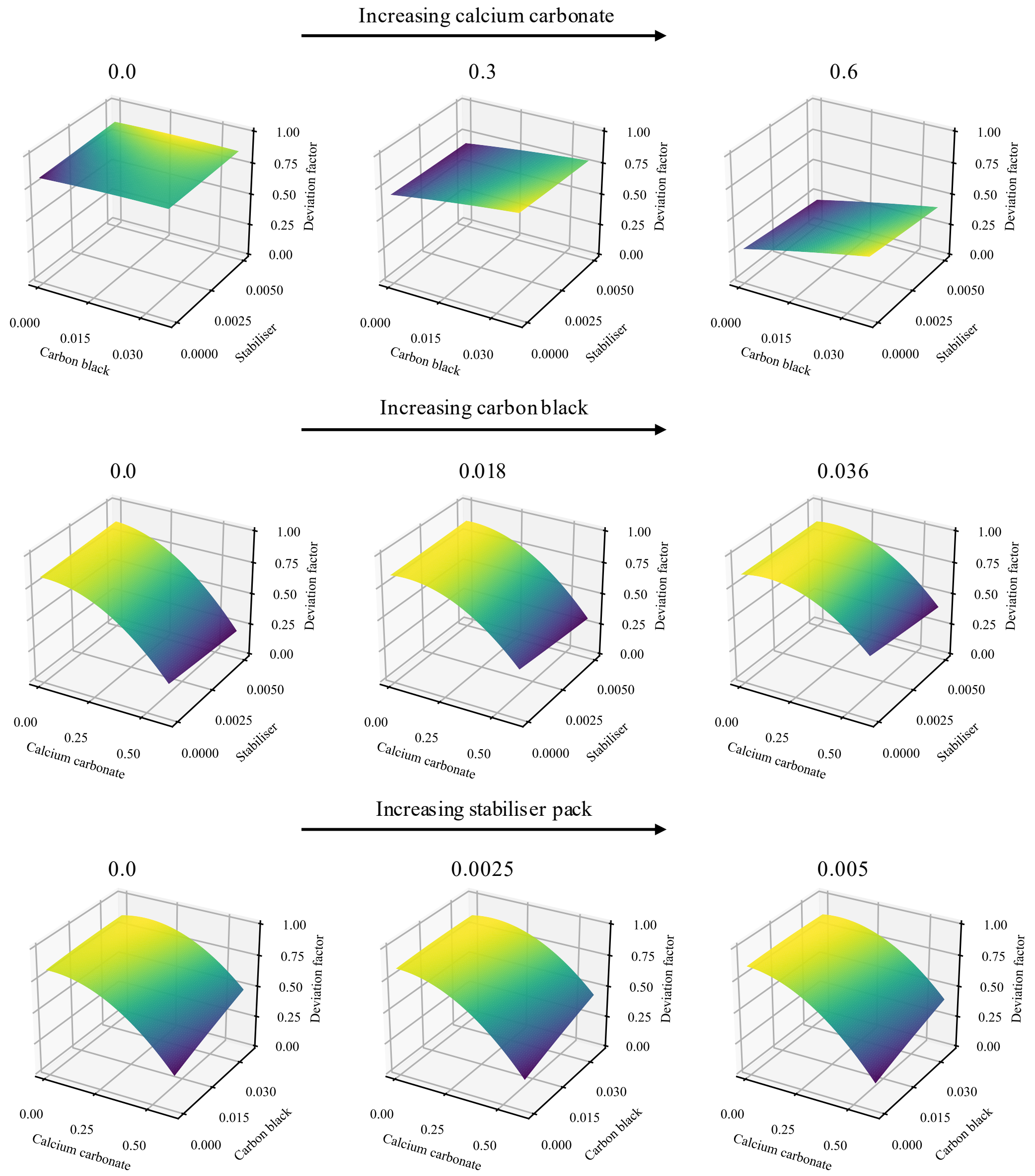

Figure S18. Modelled influence of the formulation variables on the deformation factor of WL specimens. Note the similarity to the inverse of the YM figures (Figure 3 in the main text and Figure S8), with the strong CC-related curvature and the improvements brought about by CB in the presence of CC.

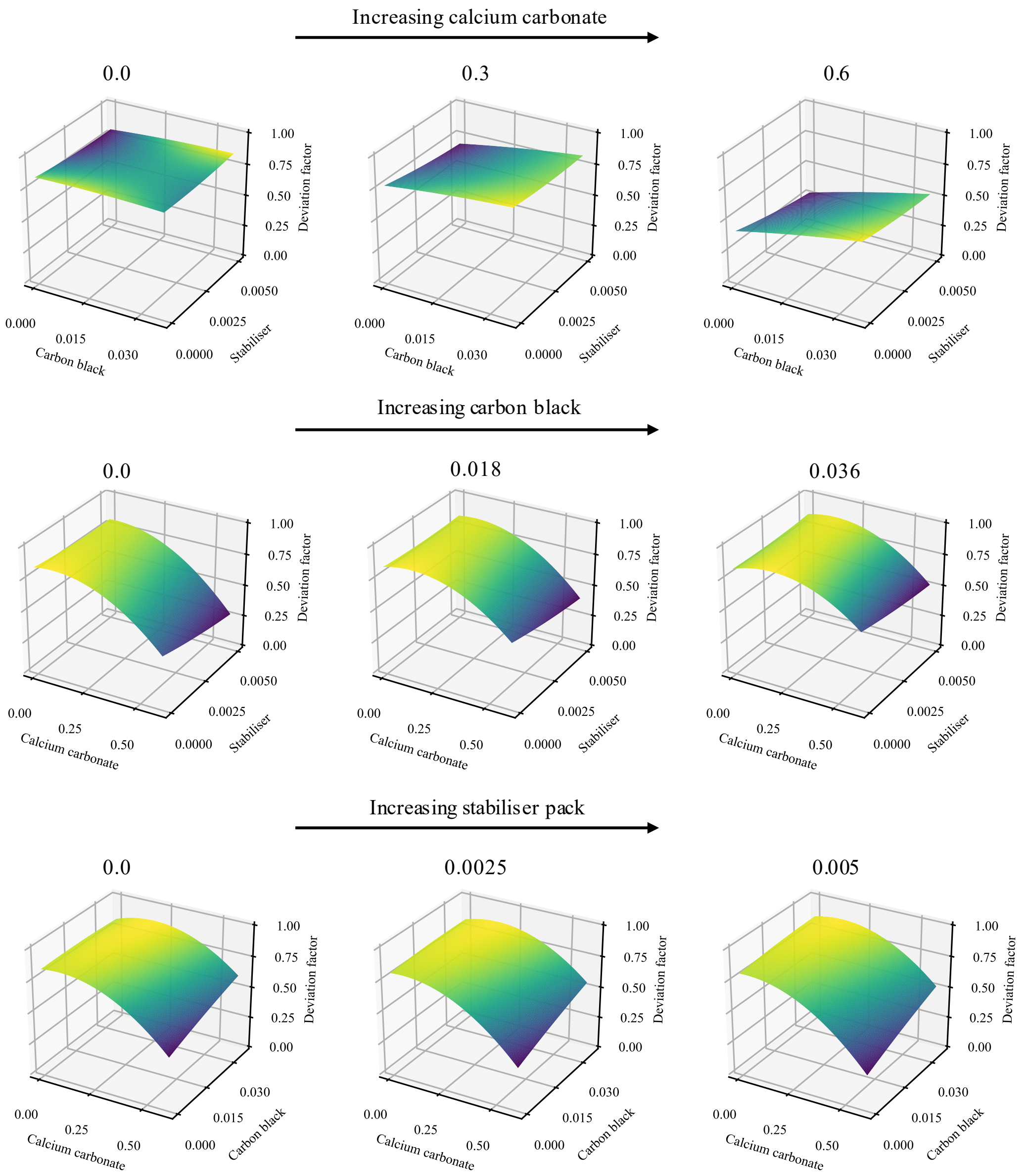

Figure S19. Modelled influence of the formulation variables on the deformation factor of REF specimens. Note the similarity to Figure S18, albeit with increased elongation at yield at a 30 % CC loading. This results in a model that appears very linear, indicating insubstantial matrix/additive interactive effects.

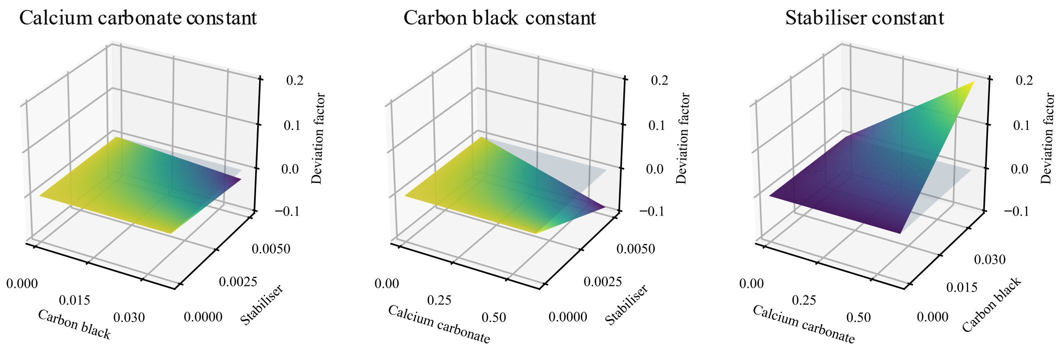

Figure S20. Modelled influence of the interactive effects of the formulation variables on the deformation factor of WL specimens. Note the very strong synergism between CB and CC, in addition to the moderate antagonism between CC and SP.

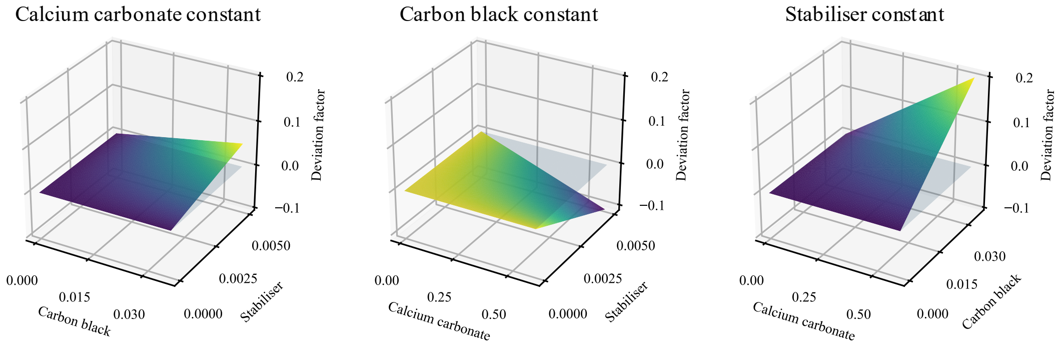

Figure S21. Modelled influence of the interactive effects of the formulation variables on the deformation factor of REF specimens. Note the similarity to Figure S20, albeit with apparent synergism between CB and SP. The latter, though, has very low statistical significance.

S7.5. Elongation at break

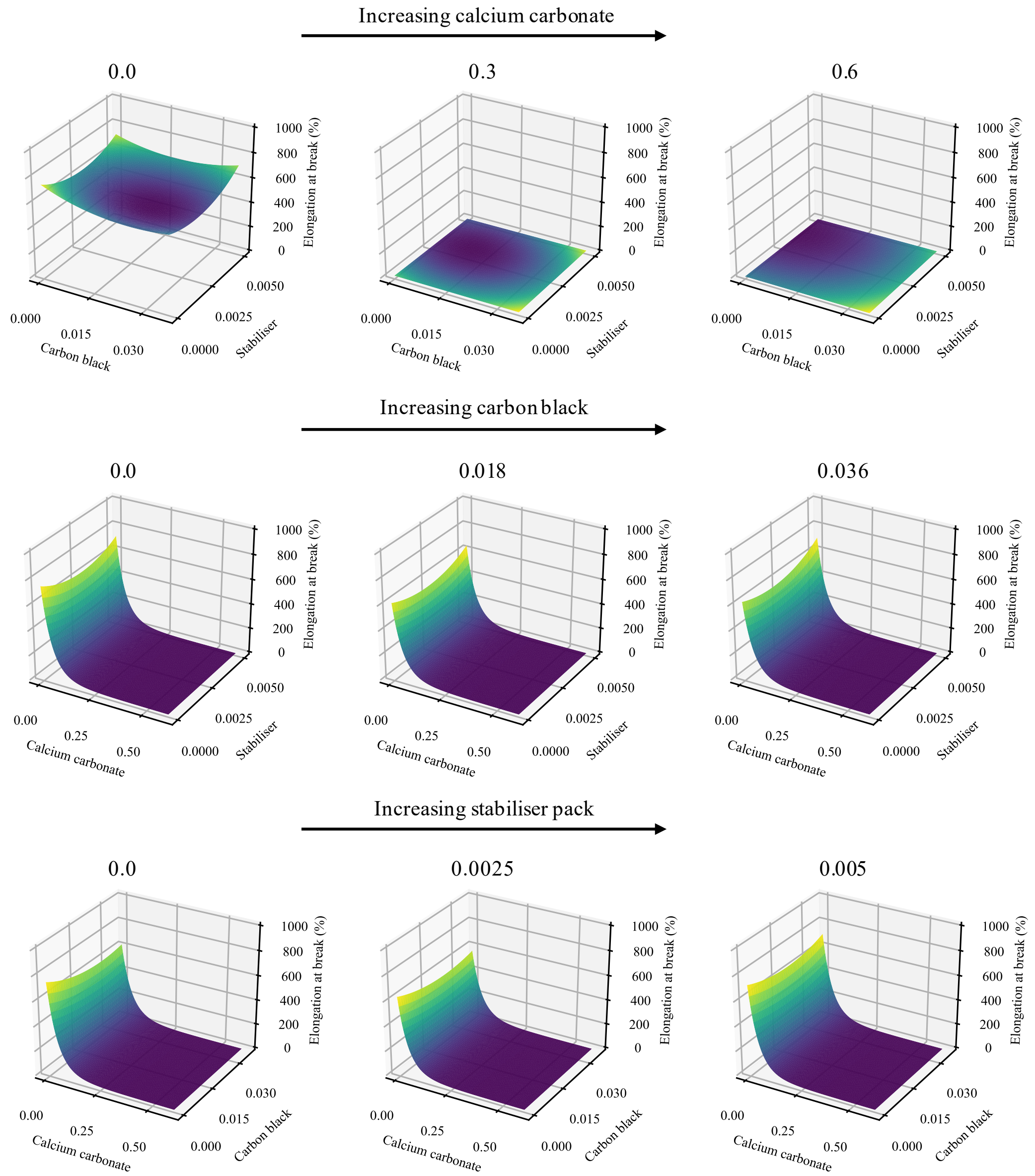

Figure S22. Modelled influence of the formulation variables on the elongation at break of WL specimens. Note the extreme decrease as a result of increasing CC loading. Further, there exist fairly insubstantial effects owing to the other variables, with the most notable the slight increases brought about by CB in the presence of CC.

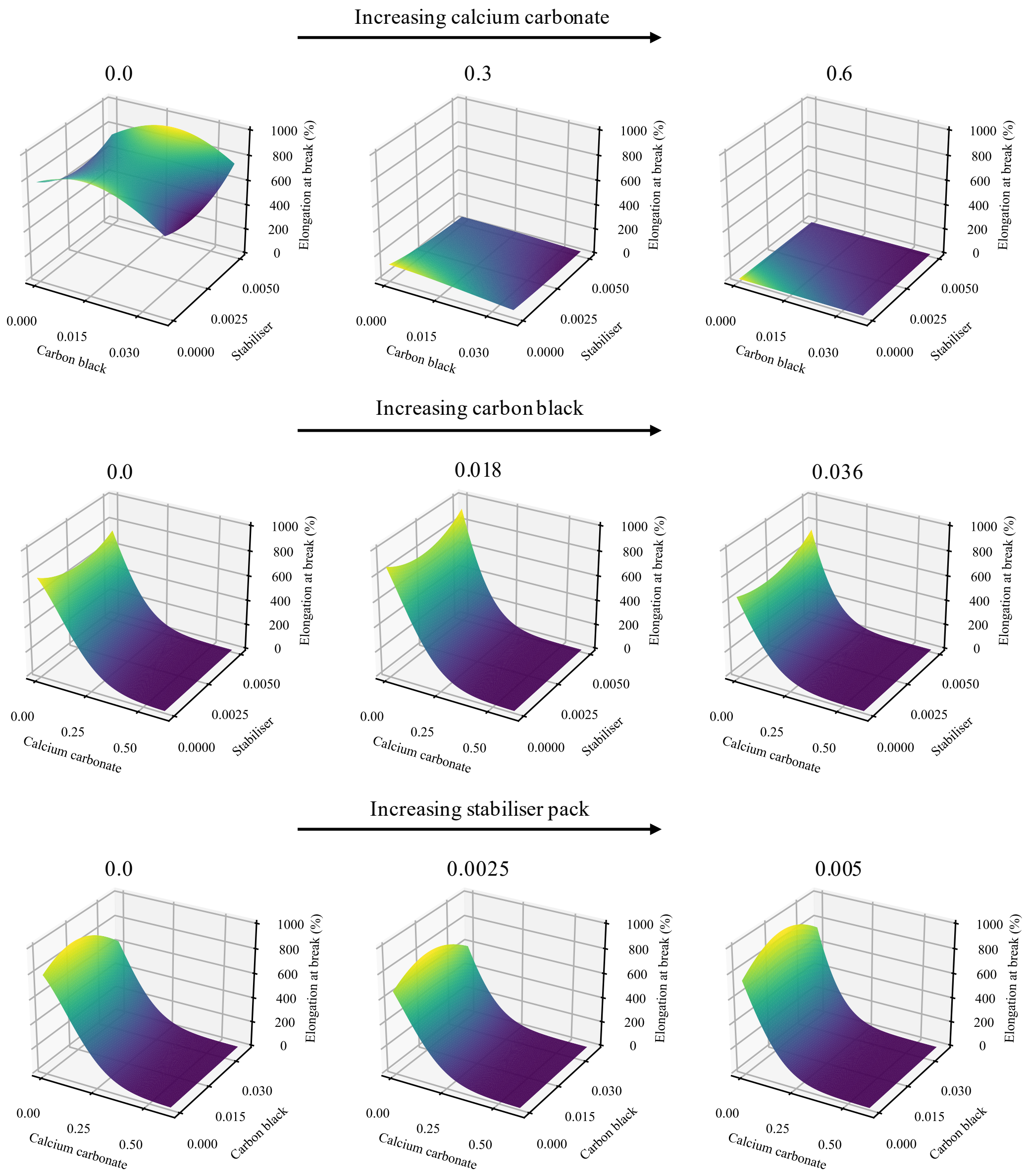

Figure S23. Modelled influence of the formulation variables on the elongation at break of REF specimens. Note the slightly decreased severity of the decrease with increasing CC as compared to that seen in Figure S22. Also note, however, the deviations from the experimental data that may be seen, here most clearly in the 0-CC behaviour.

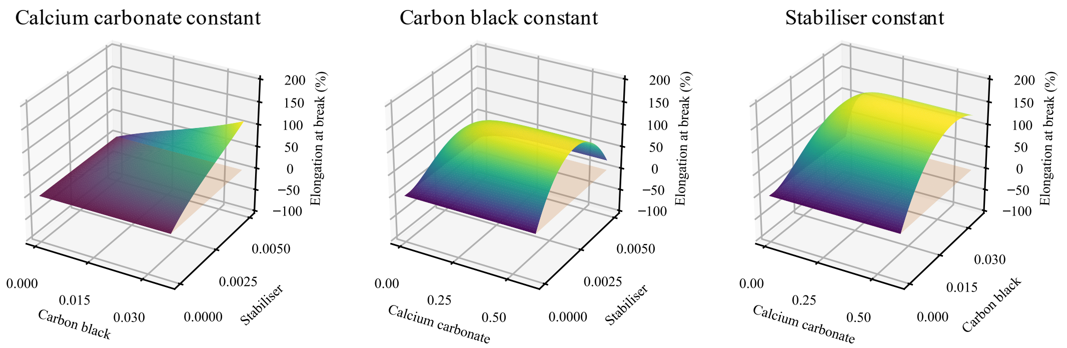

Figure S24. Modelled influence of the interactive effects of the formulation variables on the elongation at break of WL specimens. Note the distinct synergies between the compounds, particularly that between CB and CC. The parabolic behaviour seen between CC and SP is also curious, suggesting an initial synergism rapidly tending towards antagonism at higher loadings than those investigated here.

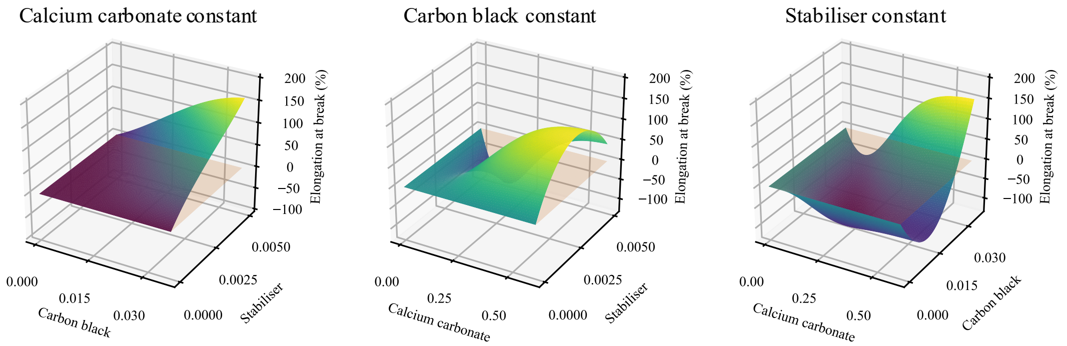

Figure S25. Modelled influence of the interactive effects of the formulation variables on the elongation at break of REF specimens. While the behaviour exhibited here is extremely complex, it is not representative—as can be seen in Figure 14 in the primary paper.

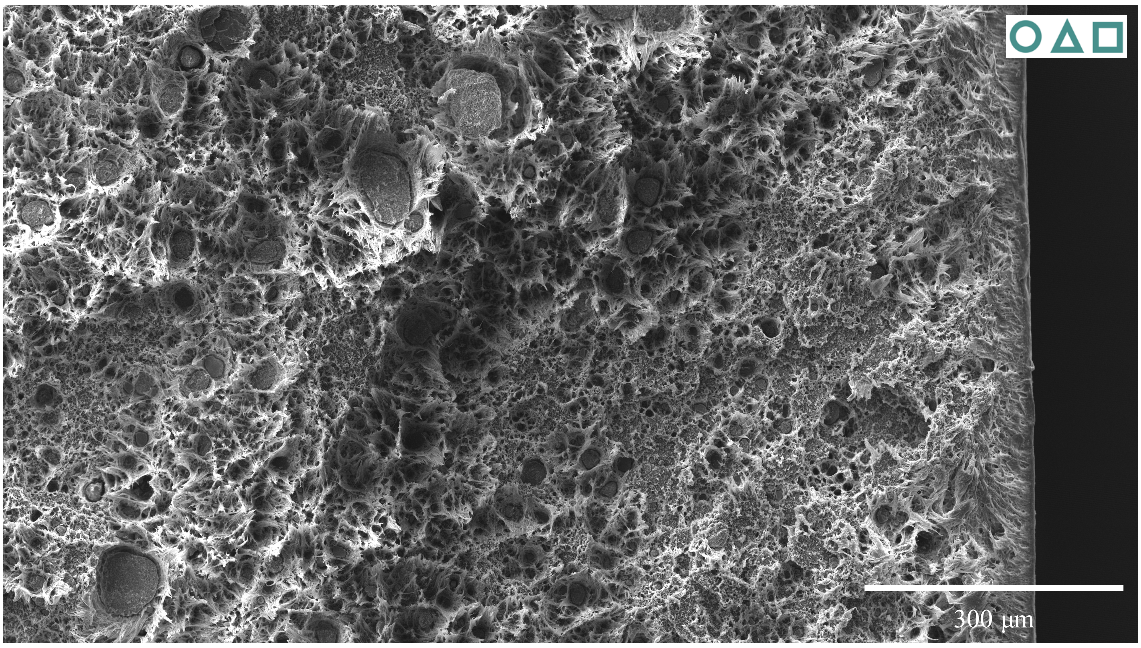

Figure S26. SEM micrograph for a general overview of the failure morphology of a 1.8-CB/30-CC/0.25-SP WL specimen, with a focus on topology. Particularly notable, here, is the smooth section on the right periphery that may be associated with the v-notch, in addition to the progression of failure mechanisms from the periphery of the material inwards.

S8. Additional SEM micrographs

References

1. OMYA Hydrocarb® 95 T - OG. Product information sheet, Omya GmbH, Cologne, Germany, 2009.
2. Carbon Black Pigments for Polymers: Americas. Product information sheet, Orion Engineered Carbons, Texas, 2013.

© 2021 by the authors. Licensee MDPI, Basel, Switzerland. This article is an open access article distributed under the terms and conditions of the Creative Commons Attribution (CC BY) license (http://creativecommons.org/licenses/by/4.0/).

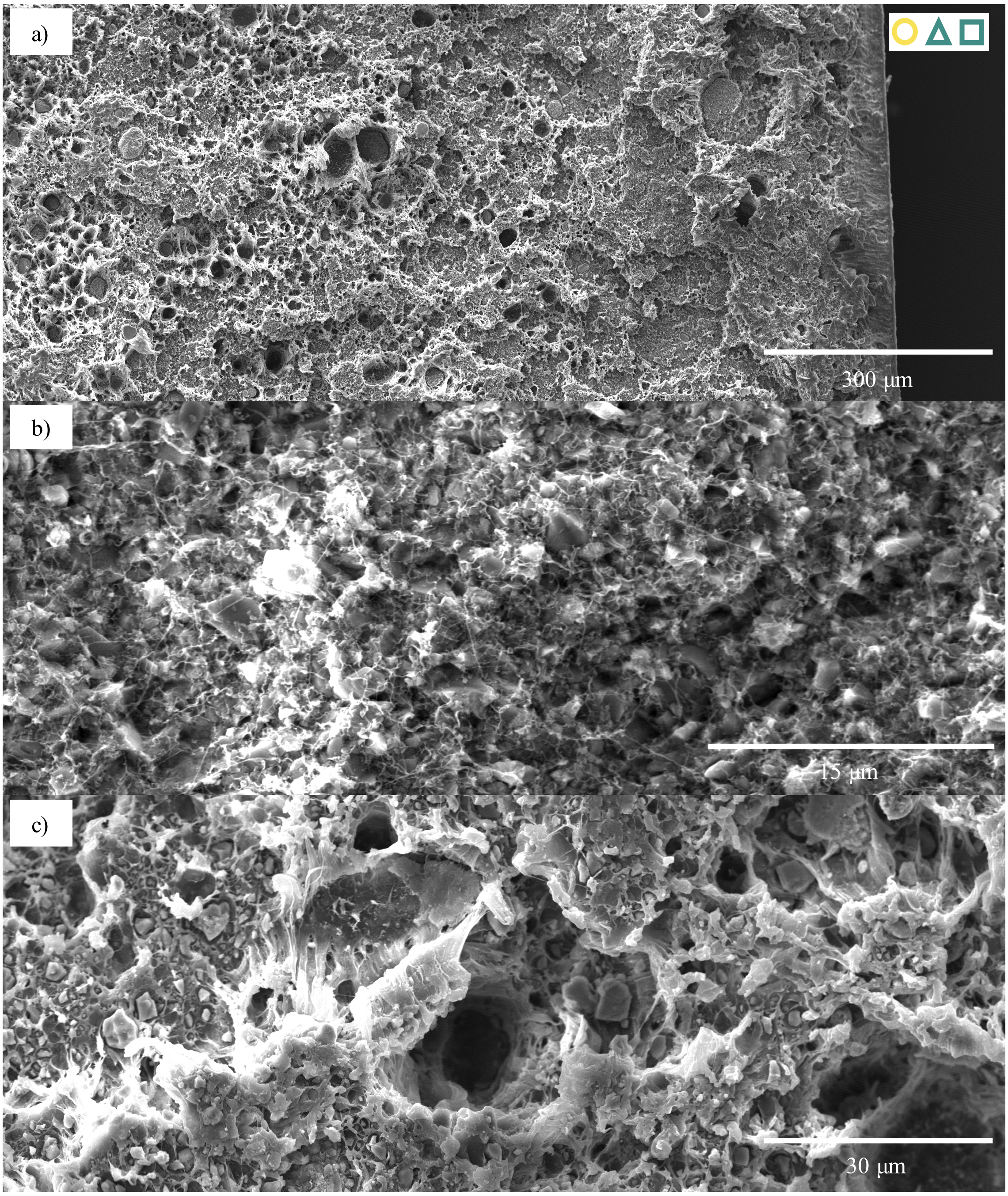

Figure S27. SEM micrographs of the failure regions of a 0-CB/60-CC/0-SP REF specimen: a) general near-perimeter morphology showing shift from unstable crack propagation towards fibrillation; b) detail of nanofibrillation and particle effects in unstable-crack region; c) filler debonding and pull-out orifice.
